# *Cdyl* Deficiency Brakes Neuronal Excitability and Nociception through Promoting *Kcnb1* Transcription in Peripheral Sensory Neurons

**DOI:** 10.1002/advs.202104317

**Published:** 2022-02-04

**Authors:** Zhao‐Wei Sun, Jarod M. Waybright, Serap Beldar, Lu Chen, Caroline A. Foley, Jacqueline L. Norris‐Drouin, Tian‐Jie Lyu, Aiping Dong, Jinrong Min, Yu‐Pu Wang, Lindsey I. James, Yun Wang

**Affiliations:** ^1^ Neuroscience Research Institute and Department of Neurobiology School of Basic Medical Sciences Key Laboratory for Neuroscience Ministry of Education/National Health Commission and State Key Laboratory of Natural and Biomimetic Drugs Peking University Beijing 100083 China; ^2^ Institute of Military Cognitive and Brain Sciences Academy of Military Medical Sciences Beijing 100039 China; ^3^ Center for Integrative Chemical Biology and Drug Discovery Division of Chemical Biology and Medicinal Chemistry UNC Eshelman School of Pharmacy University of North Carolina at Chapel Hill Chapel Hill NC 27599 USA; ^4^ Structural Genomics Consortium University of Toronto 101 College Street Toronto Ontario M5G 1L7 Canada; ^5^ Hubei Key Laboratory of Genetic Regulation and Integrative Biology School of Life Sciences Central China Normal University Wuhan Hubei 430079 China; ^6^ Department of Physiology University of Toronto Toronto Ontario M5S 1A8 Canada; ^7^ PKU‐IDG/McGovern Institute for Brain Research Peking University Beijing 100871 China

**Keywords:** chromodomain Y‐like, epigenetic regulation, K_v_2.1 channel, novel chromodomain Y‐like antagonist, pain sensation

## Abstract

Epigenetic modifications are involved in the onset, development, and maintenance of pain; however, the precise epigenetic mechanism underlying pain regulation remains elusive. Here it is reported that the epigenetic factor chromodomain Y‐like (CDYL) is crucial for pain processing. Selective knockout of CDYL in sensory neurons results in decreased neuronal excitability and nociception. Moreover, CDYL facilitates histone 3 lysine 27 trimethylation (H3K27me3) deposition at the Kcnb1 intron region thus silencing voltage‐gated potassium channel (K_v_) subfamily member K_v_2.1 transcription. Loss function of CDYL enhances total K_v_ and K_v_2.1 current density in dorsal root ganglia and knockdown of K_v_2.1 reverses the pain‐related phenotypes of Cdyl deficiency mice. Furthermore, focal administration of a novel potent CDYL antagonist blunts nociception and attenuates neuropathic pain. These findings reveal that CDYL is a critical regulator of pain sensation and shed light on the development of novel analgesics targeting epigenetic mechanisms.

## Graphical abstract

Chromodomain Y‐like (CDYL) in peripheral sensory neurons is indispensable for pain sensation. Downregulation of peripheral CDYL decreases neuronal excitability and elevates pain threshold. One of the critical mechanisms is that loss of CDYL represses histone 3 lysine 27 trimethylation (H3K27me3) deposition at *Kcnb1* intron region and facilitates its transcription. More importantly, focal delivery of a novel CDYL antagonist successfully attenuates neuropathic pain, providing probability for clinical translation.

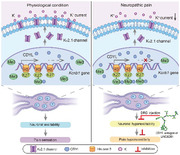

## 1 Introduction

Chronic pain is a major health problem accompanied with emotional disturbance, medicine addiction, and economic burden. The complexity of pain mechanisms and severe side effects of current analgesics lead to a lack of effective strategies for pain management. Accumulating evidence has emerged to support the potential role of epigenetics in diverse pain conditions.^[^ ^1^ ^]^ Epigenetic modifications including histone modifications, DNA methylation and noncoding RNAs are altered by peripheral noxious stimuli, resulting in changes of the expression of pain‐related gene and the occurrence of pain.^[^ ^1^, ^2^ ^]^

Dorsal root ganglia (DRG) contain first‐order somatosensory neurons and are responsible for transmitting peripheral nociceptive signals to central terminals.^[^ ^3^ ^]^ Epigenetic alterations in DRG are critical for the onset, development, and maintenance of pain. For example, spinal nerve ligation (SNL) elevated acetyl‐histone3/4 (H3/4) expression in injured DRG;^[^ ^4^ ^]^ impairment of ion channels and opioid receptors induced by altered epigenome were also observed in DRG under neuropathic pain state.^[^ ^5^, ^6^ ^]^ Since the initial pathological changes in DRG are the main triggers for chronic pain, intervention of DRG epigenetic patterns may effectively prevent the development of chronic pain. For instance, intrathecal injection of suberoylanilide hydroxamic acid (SAHA), a histone deacetylase (HDAC) inhibitor, attenuated mechanical hypersensitivity;^[^ ^7^ ^]^ Decitabine, a DNA methyltransferase (DNMT) inhibitor, ameliorated bone cancer pain by intrathecal administration;^[^ ^8^ ^]^ DRG microinjection of arginine methyltransferase siRNA or inhibitor elicited antinociception.^[^ ^9^ ^]^ Therefore, investigating the involvement of DRG epigenetics in nociceptive processing will be beneficial for chronic pain management.

Chromodomain Y‐like (CDYL) protein is recognized as a transcriptional corepressor, containing an amino‐terminal canonical chromodomain and a carboxy‐terminal enoyl‐coenzyme A (CoA) hydratase/isomerase catalytic domain.^[^ ^10^ ^]^ CDYL acts as a reader protein of repressive histone marks through its chromodomain and has been reported to exhibit histone acetyltransferase activity.^[^ ^11^, ^12^, ^13^, ^14^ ^]^ Although CDYL was first reported to function as a regulator of spermatogenesis,^[^ ^14^, ^15^ ^]^ recent researches have established that CDYL plays a key role in controlling neuronal intrinsic excitability. Our previous study indicated that knockdown of CDYL in cortical neurons caused abnormal spontaneous firing and increased the susceptibility to epilepsy;^[^ ^16^ ^]^ Liu et al. also reported that overexpressing CDYL inhibited voltage‐gated sodium channel (Na_v_) subfamily member Na_v_1.6 channel transcription thus leading to reduced neuronal excitability.^[^ ^17^ ^]^ Since abnormal excitability of sensory neurons is a hallmark of pain states, we speculate that CDYL in peripheral nociceptors may participate in pain processing.

In the present study, we identified a critical role of peripheral CDYL in the pain sensation for the first time. Downregulation of CDYL in DRG neurons enhanced the tolerance to noxious stimuli under basal and pain states. Notably, in contrast to its impact on the central nerve system (CNS), *Cdyl* deficiency suppressed neuronal excitability in peripheral sensory neurons. Furthermore, genome‐wide sequencing indicated that voltage‐gated potassium channel (K_v_) subfamily member K_v_2.1 channel is necessary for the function of CDYL in regulation of pain. CDYL repressed K_v_2.1 transcription by promoting histone 3 lysine 27 trimethylation (H3K27me3) at the *Kcnb1* intron region and knockdown of K_v_2.1 reversed the pain‐related phenotypes of *Cdyl* deficiency mice. Finally, focal delivery of a new CDYL antagonist (UNC6261) to DRG successfully inhibited neuronal excitability and relieved pain hypersensitivity. Taken together, our findings reveal a novel epigenetic mechanism of peripheral CDYL in the pain processing, providing a promising avenue for clinical treatment.

## 2 Results

### 2.1 CDYL is Widely Distributed in DRG

**Figure 1:**
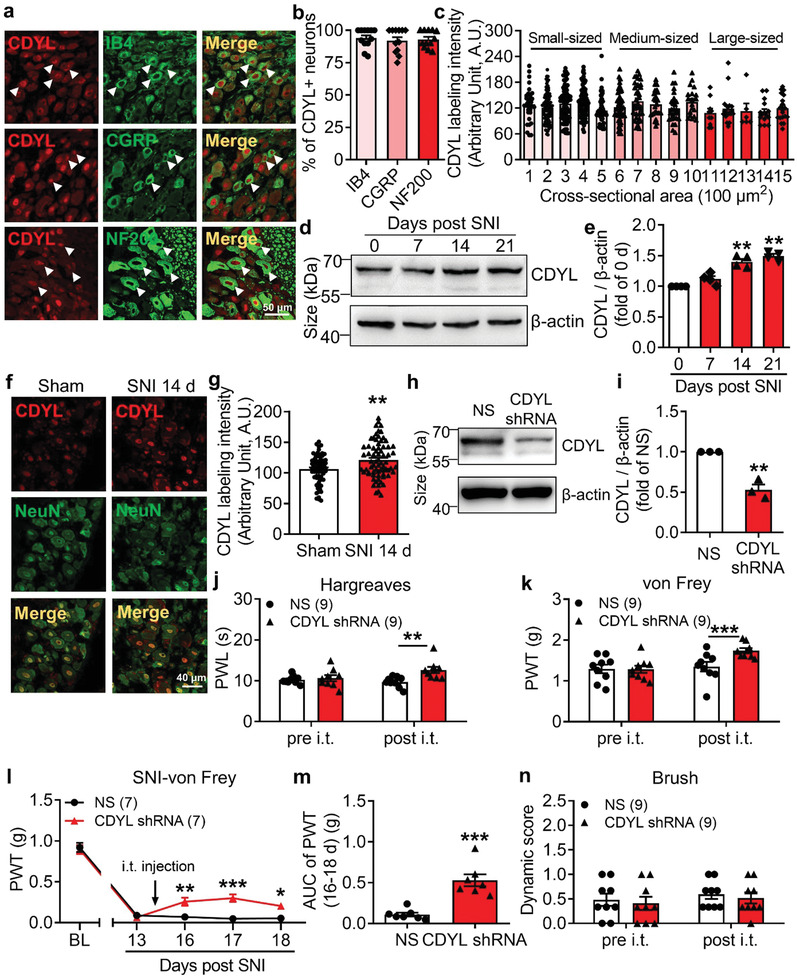
Peripheral CDYL is required for pain processing. a) Representative images of CDYL (red) and CGRP (green), IB4 (green), or NF200 (green) immunostaining in DRG. Scale bar, 50 µm. b) Quantification of the percentages of CDYL‐positive (CDYL+) neurons among those labeled with indicated markers. *n* = 3 biological replicates. One‐way ANOVA with Turkey's post‐hoc, no statistical significance. c) Labeling intensity of CDYL in DRG neurons with different cross‐sectional area. *n* = 4 mice. One‐way ANOVA with Turkey's post‐hoc, no statistical significance. d) Representative images of CDYL expression in ipsilateral L4‐5 DRG on days 0, 7, 14, and 21 after SNI by western blotting. e) Quantification of the CDYL protein level in (d). *n* = 4 biological replicates. One‐way ANOVA with Turkey's post‐hoc, **p < 0.01. f) Representative images of CDYL (red) and NeuN (green) immunostaining in DRG on day 14 after sham and SNI. Scale bar, 40 µm. g) Quantification of the CDYL labeling intensity in (f). *n* = 3 mice. Student's unpaired *t* test, **p < 0.01. h) Representative images of CDYL expression in DRG after transfection with indicated plasmids. i) Quantification of the CDYL protein level in (h). *n* = 4 biological replicates. Student's paired *t* test, **p < 0.01. j) Basal paw withdrawal latency (PWL) to radiant heat stimuli before and after intrathecal (i.t.) injection was assessed by Hargreaves's method. Two‐way ANOVA with Sidak's post‐hoc test, **p < 0.01. k) Basal paw withdrawal threshold (PWT) to mechanical stimuli was assessed by von Frey test. Two‐way ANOVA with Sidak's post‐hoc test, ***p < 0.001. l) Time course of PWT of the ipsilateral hind paw after SNI. Two‐way ANOVA with Sidak's post‐hoc test, *p < 0.05, **p < 0.01, ***p < 0.001. m) Area under the curve (AUC) from days 16 to 18 after SNI was compared. Student's unpaired *t* test, ***p < 0.001. n) Basal touch sensitivity was assessed by brush test. Two‐way ANOVA with Sidak's post‐hoc test, no statistical significance. Data are the mean ± SEM.

DRG sensory neurons exhibit a wide range of morphologies and functional features, which allow the discrimination between various types of sensations. For instance, DRG neurons with large cell bodies are responsible for proprioception and mechanoreception, whereas those with smaller size are nociceptors relaying for painful signals.^[^ ^18^, ^19^ ^]^ To decipher the role of CDYL in primary sensory neurons, we first assessed the cellular distribution of CDYL in mice DRG. The immunostaining result revealed that CDYL was expressed ubiquitously in the nucleus of different‐sized DRG neurons (**Figure** **1**a). The quantification analysis indicated that 93.95% of isolectin B4 (IB4)‐positive neurons, 91.98% of calcitonin gene related peptide (CGRP)‐positive neurons and 93.10% of neurofilament 200 (NF200)‐positive neurons expressed CDYL, respectively (Figure 1b). Additionally, there was no difference among the staining intensity of different subpopulations of DRG neurons (Figure 1c).

### 2.2 Loss of CDYL in DRG Increases Pain Threshold

Considering its expression in peripheral nociceptors, we speculated that CDYL could contribute to the pain processing. First, western blot analysis indicated that CDYL expression in ipsilateral lumbar 4–5 (L4–5) DRG was elevated on days 14 and 21 after spared nerve injury (SNI) (Figure 1d,e). The observed increment in CDYL expression on day 14 after SNI was confirmed by immunostaining (Figure 1f,g). To further determine the role of CDYL in pain etiology, we intrathecally injected CDYL‐shRNA plasmids to knockdown endogenous CDYL expression in mice DRG. The efficiency of CDYL knockdown was verified by western blotting (Figure 1h,i). The basal thermal and mechanical threshold were markedly increased on day 2 after intrathecal injection of CDYL‐shRNA in mice (Figure 1j,k). Moreover, knockdown of CDYL attenuated mechanical allodynia induced by peripheral nerve injury (Figure 1l,m). We also injected CDYL‐EGFP plasmids intrathecally to overexpress exogenous CDYL in DRG. Efficient expression of the plasmids was confirmed by western blotting (Figure S1a,b, Supporting Information). Induction of exogenous CDYL expression enhanced the responses to noxious mechanical and thermal stimuli in mice (Figure S1c,d, Supporting Information), but did not aggravate neuropathic pain (Figure S1e,f, Supporting Information). Additionally, CDYL expression did not alter tactile sensitivity (Figure 1n and Figure S1g, Supporting Information).

**Figure 2:**
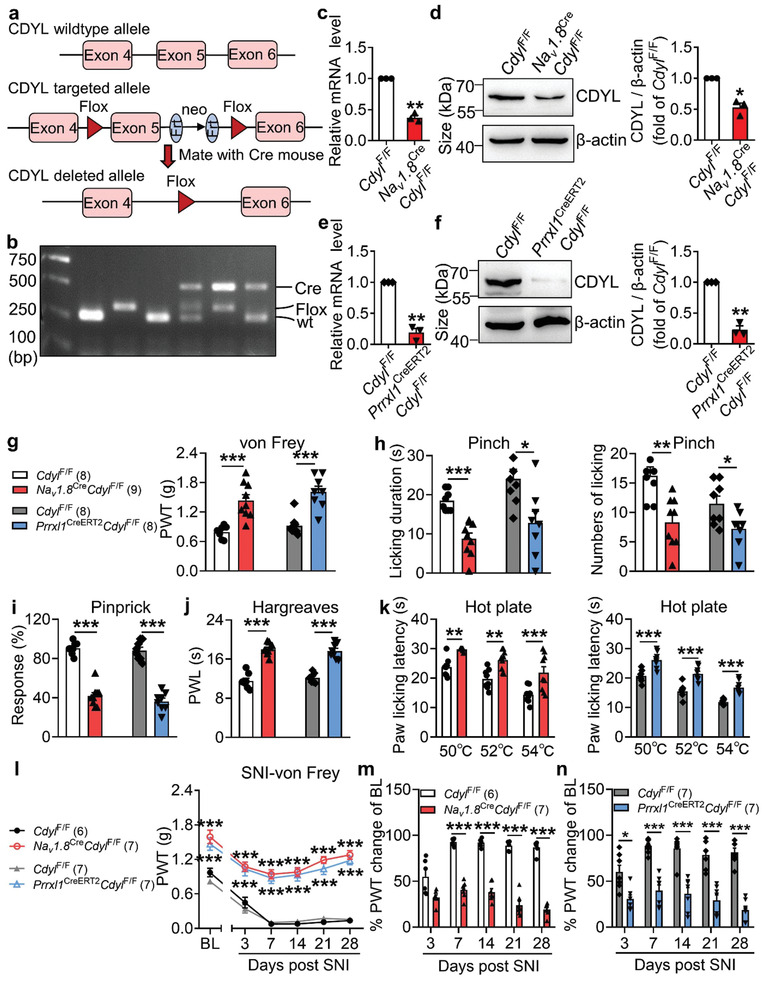
Loss of CDYL in male mice DRG decreases pain sensitivity. a) Strategy of conditional *Cdyl* knockout mice. b) PCR analysis of genomic DNA from intercrosses. c,e) The *Cdyl* mRNA level in DRG from homozygous genotypes of the *Cdyl*‐floxp mice crossed with *Na_v_1.8‐*Cre (*Na_v_1.8* ^Cre^ *Cdyl* ^F/F^) (c) or *Prrxl1‐*CreER^T2^ (*Prrxl*1^CreERT2^ *Cdyl* ^F/F^) (e) mice was examined by qRT‐PCR. *n* = 4 biological replicates. Student's paired *t* test, **p < 0.01. d,f) Representative images of CDYL expression in *Na_v_1.8* ^Cre^ *Cdyl* ^F/F^ mice (d) or *Prrxl*1^CreERT2^ *Cdyl* ^F/F^ mice (f) by western blotting (left). Quantification of CDYL expression in the left image (right). *n* = 4 biological replicates. Student's paired *t* test, *p < 0.05, **p < 0.01. g) Basal PWT was assessed by von Frey test. Student's unpaired *t* test, ***p < 0.001. h) Licking durations (left) and numbers (right) responding to pinching stimuli were measured. Student's unpaired *t* test, *p < 0.05, **p < 0.01, ***p < 0.001. i) Response to pinprick stimuli were assessed by pinprick test. Student's unpaired *t* test, ***p < 0.001. j) Basal PWL was assessed by Hargreaves's method. Student's unpaired *t* test, ***p < 0.001. k) Response to noxious heat stimuli was assessed by hot plate test. Student's unpaired *t* test, **p < 0.01, ***p < 0.001. l) Time course of PWT after SNI. Two‐way ANOVA with Sidak's post‐hoc test, ***p < 0.001. m,n) Percentages of PWT changes of baseline at different time points after SNI. Two‐way ANOVA with Sidak's post‐hoc test, *p < 0.05, ***p < 0.001. Data are the mean ± SEM.

To corroborate the contribution of CDYL to pain processing, conditional *Cdyl* knockout mice were generated using Cre‐Loxp recombination system (**Figure** **2**a). Because global deletion of *Cdyl* in mice causes embryonic lethality,^[^ ^20^ ^]^ we generated two types of DRG conditional *Cdyl* knockout (cKO) mice. First, we deleted *Cdyl* in DRG nociceptors by crossing homozygous *Cdyl*‐floxp mice (*Cdyl* ^F/F^) with a *Na_v_1.8*‐Cre mouse line (*Na_v_1.8* ^Cre^).^[^ ^21^ ^]^ Then we deleted *Cdyl* in DRG neurons with no special preference by crossing *Cdyl* ^F/F^ mice with a *Prrxl1* tamoxifen‐inducible Cre line (*Prrxl*1^CreERT2^).^[^ ^22^ ^]^ The knockout efficiency in *Na_v_1.8* ^Cre^ *Cdyl* ^F/F^ and *Prrxl*1^CreERT2^ *Cdyl* ^F/F^ mice were validated by RT‐PCR and western blotting, respectively (Figure 2b–f).

Next, a series of behavioral tests were conducted to characterize *Cdyl*‐deficient mice. No significant difference was observed in the open field test and rotarod test (Figure S2a,b, Supporting Information), suggesting that *Cdyl* deletion did not generate motor disability. Subsequently, we measured pain‐like behaviors among genotypes. The sensitivities to noxious mechanical and thermal stimuli were blunted in *Cdyl*‐deficient mice under normal condition (Figure 2g–k). Consistently, pain hypersensitivity induced by SNI was significantly mitigated by deletion of *Cdyl* (Figure 2l–n). In addition, we found that *Cdyl* deficiency in DRG had no influence on the touch and cold sensations (Figure S2c–h, Supporting Information). Similar results were obtained in female knockout mice (Figure S3, Supporting Information). These findings strongly demonstrate that CDYL is required for the nociceptive signaling pathway.

### 2.3 Loss of CDYL in DRG Inhibits Neuronal Excitability

**Figure 3:**
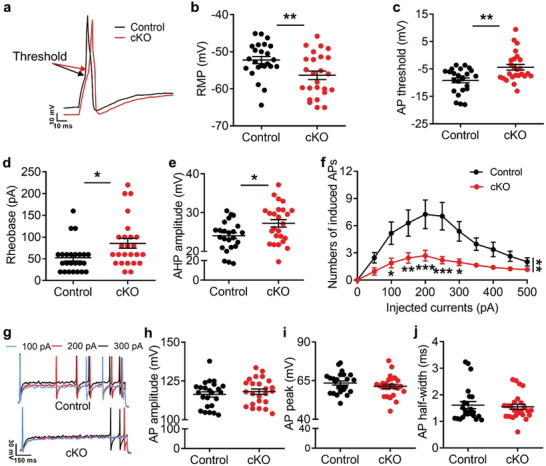
Loss of CDYL in male mice DRG inhibits neuronal excitability. a) Representative traces of evoked action potentials (APs) in DRG neurons. Scale bars, 10 ms and 10 mV. b–e) The resting membrane potential (RMP) (b), AP threshold (c), current threshold (rheobase) (d) and the amplitude of after‐hyperpolarization (AHP) (e) obtained from DRG neurons of male *Cdyl* cKO mice and their littermate controls. Student's unpaired *t* test, *p < 0.05, **p < 0.01. f,g) Numbers (f) and representative traces (g) of APs induced by indicated currents. Scale bars, 150 ms and 30 mV. Two‐way ANOVA, group effect: **p < 0.01, shown at the end of the lines; post‐test: Sidak's post‐hoc test, *p < 0.05, **p < 0.01, ***p < 0.001, shown below the lines. h–j) The amplitude (h), peak (i), and half‐width (j) of AP in DRG neurons of *Cdyl* cKO mice and controls. Student's unpaired *t* test, no statistical significance. *n*_control_ = 24; *n*_cKO_ = 25. Data are presented as mean ± SEM.

Tissue inflammation or injury leads to the sensitization or hyperexcitability of DRG and ultimately gives rise to pain.^[^ ^23^ ^]^ As previous studies reported that CDYL could inhibit excitability of hippocampal and cortical neurons,^[^ ^16^, ^17^ ^]^ we performed electrophysiological experiment to test whether *Cdyl* deficiency influences intrinsic electrical properties of primary sensory neurons as well. Surprisingly, the resting membrane potential (RMP) in male *Cdyl* cKO (*Prrxl*1^CreERT2^ *Cdyl* ^F/F^) mice was significantly decreased by 4.15 mV compared to controls (**Figure** **3**a,b). The action potential (AP) threshold, current threshold and after‐hyperpolarization (AHP) amplitude in male *Cdyl* cKO mice were increased by 52.36%, 63.46%, and 14.12% relative to controls, respectively (Figure 3c–e). The average numbers of action potentials (APs) evoked by 100–300 pA currents were apparently reduced by deletion of *Cdyl* (Figure 3f,g). Additionally, there was no statistical significance in AP peak, amplitude, and half‐width (Figure 3h–j). Similar phenomena were also observed in female knockout mice (Figure S4, Supporting Information). Collectively, these results illustrate that in contrast to the effect on the CNS, loss of CDYL decreases neuronal excitability in DRG.

### 2.4 CDYL Represses K_v_2.1 Transcription in DRG

**Figure 4:**
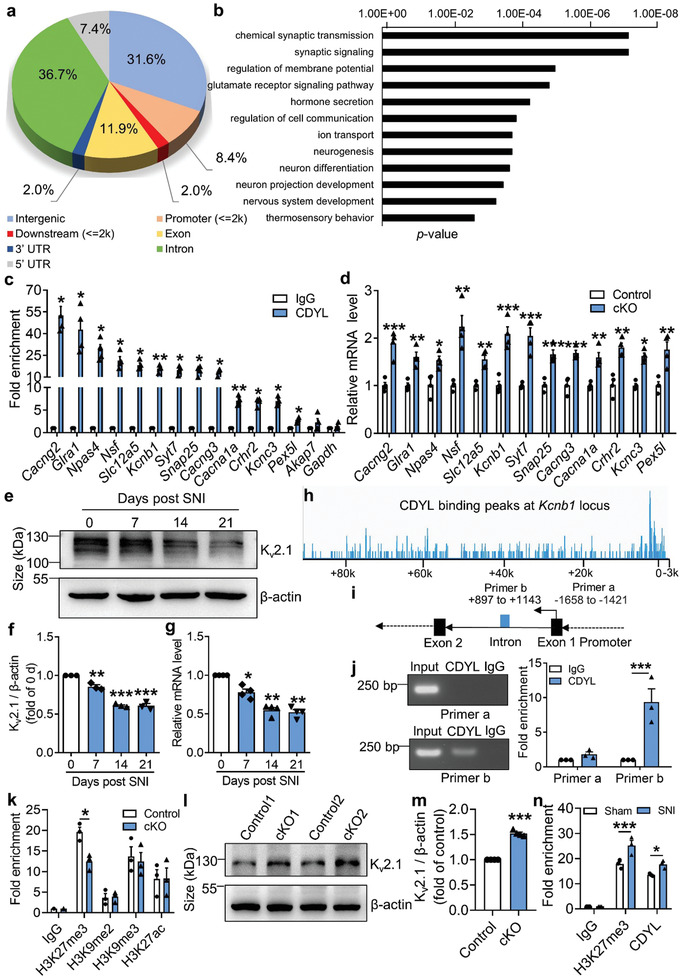
CDYL suppresses *Kcnb1* transcription by promoting H3K27me3 at the intron region. a) The genomic distribution of CDYL binding locus was determined by ChIP‐seq analysis. b) The biological process of the identified genes was classified by GO analysis. c) The ChIP‐seq results were verified in mice DRG by ChIP‐qPCR analysis. *n* = 3 biological replicates. Student's paired *t* test, *p < 0.05, **p < 0.01. d) The mRNA levels of representative genes in *Cdyl* cKO and control mice were measured by qRT‐PCR. *n* = 4 biological replicates. Student's paired *t* test, *p < 0.05, **p < 0.01, ***p < 0.001. e) Representative images of K_v_2.1 expression in the ipsilateral DRG after SNI by western blotting. f) Quantification of the K_v_2.1 protein level in (e). *n* = 3 biological replicates. One‐way ANOVA with Turkey's post‐hoc test, **p < 0.01, ***p < 0.001. g) The *Kcnb1* mRNA level after SNI was measured by qRT‐PCR. *n* = 4 biological replicates. One‐way ANOVA with Turkey's post‐hoc test, *p < 0.05, **p < 0.01. h) The genome‐wide snapshot of CDYL binding peaks at *Kcnb1* locus. i) Diagram of the designed primer pairs to indicated regions. j) The fold enrichment of CDYL at the *Kcnb1* intron and promoter region by semi‐qChIP (left) and ChIP‐qPCR (right). *n* = 3 biological replicates. Student's paired *t* test, ***p < 0.001. k) The fold enrichment of H3 modifications at the *Kcnb1* intron region in *Cdyl* cKO and control mice was measured by ChIP‐qPCR. *n* = 4 biological replicates. Student's paired *t* test, *p < 0.05. l) Representative images of K_v_2.1 expression in *Cdyl* cKO mice and controls. m) Quantification of the K_v_2.1 protein level in (l). *n* = 4 biological replicates. Student's paired *t* test, ***p < 0.001. n) The fold enrichment of H3K27me3 and CDYL at the *Kcnb1* intron region on day 14 after SNI was measured by ChIP‐qPCR. *n* = 4 biological replicates. Student's paired *t* test, * *p* < 0.05, *** *p* < 0.001. Data are the mean ± SEM.

To explore the molecular mechanism through which CDYL regulates nociception, we identified the downstream genes by chromatin immunoprecipitation (ChIP) combined with second generation DNA‐sequencing (ChIP‐seq) in mice DRG. 10895 CDYL‐specific binding peaks were mapped with a *p* value cutoff 10^−3^ through the Illumina Novaseq6000 sequencing platform. Genomic location preference analysis demonstrated that 31.6% of the binding sites were found at promoter‐distal regions, 36.7% were located at the exon regions, 11.9% were at the intron regions, and 8.4% were at the promoter regions (**Figure** **4**a). GO analysis of the CDYL genomic locations to identify biological themes among the associated genes revealed that they were mostly associated with neurological functions, including chemical synaptic transmission, regulation of membrane potential, and ion transport (Figure 4b).

Next, we performed ChIP‐qPCR analysis with DRG tissue to verify the ChIP‐seq results. We selected 14 genes related to neurological function, including *Kcnb1*, *Npas4*, and *Nsf*. The results indicated that CDYL was enriched at the corresponding binding sites of selected genes (Figure 4c). Considering CDYL is a transcriptional corepressor, the mRNA levels of selected genes in DRG of *Cdyl* cKO mice were determined. The results showed that the mRNA levels of these genes were dramatically upregulated in *Cdyl* cKO mice, further supporting the ChIP‐seq results (Figure 4d). Among these candidate genes, *Kcnb1/*K_v_2.1 mRNA and protein levels were significantly decreased on days 7, 14, and 21 after nerve injury (Figure 4e–g), indicating that K_v_2.1 channel is a potential downstream of CDYL in mediating nociception.

Previously, CDYL was found to repress transcription by facilitating H3K27me2/3 and H3K9me2/3 modifications or recruiting HDAC1/2 activities.^[^ ^10^, ^11^, ^12^, ^13^ ^]^ Therefore, we speculated that CDYL modulates K_v_2.1 expression by altering histone methylation or acetylation of chromatins. First, because the ChIP‐seq results indicated that CDYL binds to the intron region of *Kcnb1*, we confirmed the binding site (+897 to +1143 bp) by ChIP‐qPCR (Figure 4h–j). Moreover, we performed ChIP‐qPCR using antibodies against H3K27me3, H3K9me2, H3K9me3, and H3K27ac. A decrease in H3K27me3 levels was observed in the DRG of *Cdyl* cKO mice relative to controls, yet other remarks remained relatively unchanged (Figure 4k). Western blot analysis validated that *Cdyl* deficiency enhanced peripheral K_v_2.1 expression (Figure 4l,m). Furthermore, the H3K27me3 levels at the *Kcnb1* gene body were increased in the SNI model, resulting in the decline of K_v_2.1 expression under neuropathic pain (Figure 4n). Taken together, these results show that CDYL transcriptionally silences K_v_2.1 by recruiting H3K27me3 activity at its intron region.

### 2.5 Loss of CDYL in DRG Augments Total K_v_ and K_v_2.1 Currents

K_v_2.1 channel is well known to function as a suppressor of neuronal excitability especially when high‐frequency AP firing happens and exerts neuroprotection under various hyperexcitable conditions.^[^ ^24^, ^25^, ^26^ ^]^ DRG neurons from *Cdyl* cKO mice and controls were treated with GxTX‐1E, a specific inhibitor of K_v_2.1 channel,^[^ ^19^, ^27^ ^]^ to explore whether K_v_2.1 channel contributed to the regulation of CDYL on intrinsic excitability. Repression of functional K_v_2.1 channel did not influence the RMP and other AP parameters (Figure S5a–e, Supporting Information). However, both *Cdyl*‐deficient and control DRG neurons exhibited lower AHP amplitude and higher firing frequency in the presence of 100 nm GxTX‐1E (Figure S5f–h, Supporting Information).

**Figure 5:**
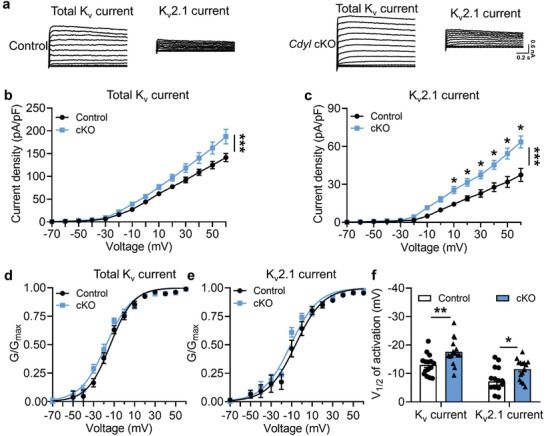
Loss of CDYL enhances total K_v_ and K_v_2.1 currents in DRG. a) Representative traces of total K_v_ (left) and K_v_2.1 current (right) in DRG neurons from control and *Cdyl* cKO mice. Scale bars, 0.2 s and 0.6 nA. b, c) Densities of total K_v_ (b) and K_v_2.1 current (c) in DRG neurons from control and *Cdyl* cKO mice. Two‐way ANOVA, group effect: ***p < 0.001, shown at the end of the lines; post‐test: Sidak's post‐hoc test, *p < 0.05, shown above the lines. d,e) The activation curves of total K_v_ (d) and K_v_2.1 current (e) in the DRG neurons from control and *Cdyl* cKO mice. f) The half‐activation values (V_1/2_) of total K_v_ and K_v_2.1 current in the DRG neurons from control and *Cdyl* cKO mice. Student's unpaired *t* test, *p < 0.05, **p < 0.01. *n*_control_ = 15, *n*_cKO_ = 15. Data are the mean ± SEM.

Next, we examined whether deletion of *Cdyl* could affect potassium currents. Whole‐cell voltage‐clamp was applied to record K_v_ currents in acutely dissociated DRG neurons. The results indicated that loss of CDYL increased total K_v_ and K_v_2.1 current densities (**Figure** **5**a–c). The activation curves (*G*–*V*) were calculated using the Boltzmann equation, showing that *Cdyl*‐deficient neurons had a more negative half‐activation voltage (Figure 5d–f). Together, these findings suggest that CDYL inhibits total K_v_ and K_v_2.1 currents, thus maintaining excitability of DRG neurons.

### 2.6 CDYL Regulates Nociception via K_v_2.1 Channel

**Figure 6:**
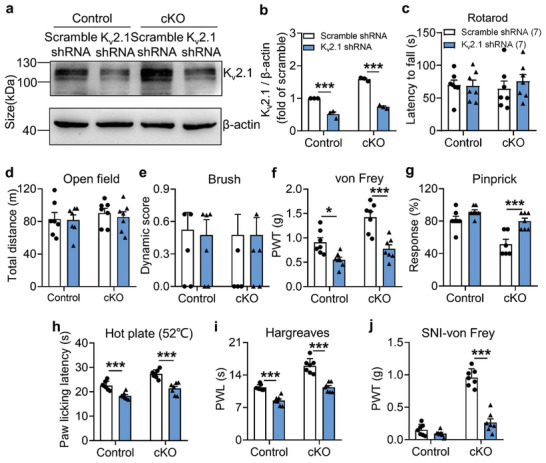
CDYL mediates nociception through K_v_2.1 channel. a) Representative images of K_v_2.1 expression in ipsilateral DRG infected with AAV‐scramble shRNA or AAV‐K_v_2.1 shRNA by western blotting. b) Quantification of the K_v_2.1 protein level in (a). *n* = 3 biological replicates. Two‐way ANOVA with Turkey's post‐hoc test, ***p < 0.001. c,d) Coordination skills and motor activities were assessed by rotarod test (c) and open field test (d), respectively. Two‐way ANOVA with Turkey's post‐hoc test, no statistical significance. e) Tactile sensitivity was assessed by brush test. Two‐way ANOVA with Turkey's post‐hoc test, no statistical significance. f) Basal PWT was assessed by von Frey test. Two‐way ANOVA with Turkey's post‐hoc test, *p < 0.05, ***p < 0.001. g) Response to mechanical stimuli was assessed by pinprick test. Two‐way ANOVA with Turkey's post‐hoc test, ***p < 0.001. h) Response to thermal stimuli was assessed by hot plate test. Two‐way ANOVA with Turkey's post‐hoc test, ***p < 0.001. i) Basal PWL was assessed by Hargreaves test. Two‐way ANOVA with Turkey's post‐hoc test, ***p < 0.001. j) Mechanical allodynia on day 14 after SNI was assessed. Two‐way ANOVA with Turkey's post‐hoc test, ***p < 0.001. Data are the mean ± SEM.

To further evaluate the pathophysiological consequences of peripheral CDYL‐K_v_2.1 signaling axis dysfunction, AAV‐K_v_2.1‐shRNA‐EGFP, and AAV‐scramble‐shRNA‐EGFP were directly microinjected into unilateral L4–5 DRG of *Cdyl* cKO mice and littermate controls. As shown by the western blot, K_v_2.1 expression was prominently downregulated in the K_v_2.1 shRNA‐infected groups 4 weeks after direct virus injection (**Figure** **6**a,b). Disruption of K_v_2.1 expression did not alter the motor function and tactile sensation (Figure 6c–e); however, K_v_2.1‐shRNA‐infected cKO mice exhibited more drastic mechanical and thermal nociceptive responses than scramble‐shRNA‐infected cKO mice (Figure 6f–i). Moreover, K_v_2.1‐shRNA‐infected cKO mice suffered from more severe hypersensitivity after SNI (Figure 6j). Similar phenomena were observed in control mice as well (Figure 6f–j). Together, these evidences emphasize the involvement of K_v_2.1 channel in antinociception caused by *Cdyl* deficiency in peripheral sensory neurons.

### 2.7 Development of a Potent CDYL Antagonist

**Figure 7:**
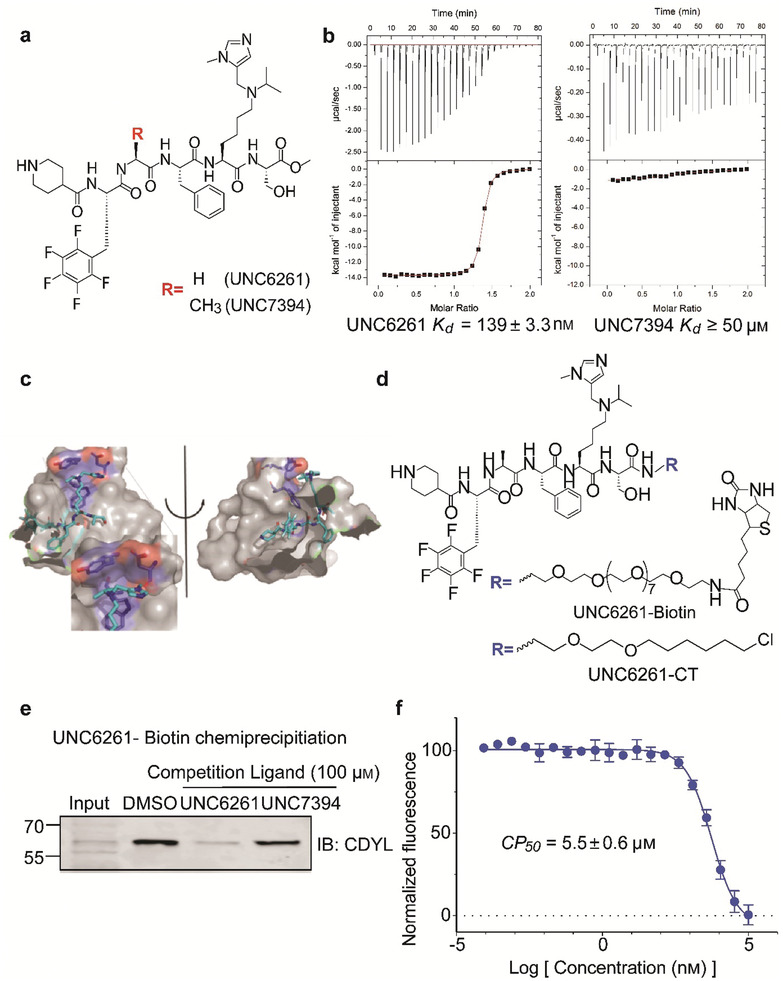
UNC6261 is a potent antagonist of the CDYL chromodomain. a) Structure of UNC6261 and negative control ligand UNC7394. b) UNC6261 potently binds the CDYL chromodomain as determined by isothermal titration calorimetry whereas UNC7394 demonstrates no measurable binding. Data are the mean ± S.D. c) X‐ray crystal structure of UNC6261 bound to CDYL highlighting the surface groove and aromatic cage (bottom left) interactions (PDB: 7N27). d) Structure of UNC6261‐Biotin and UNC6261‐CT. e) Chemiprecipitation of full‐length CDYL from MDA‐MB‐231 cell lysates via UNC6261‐Biotin in the presence of UNC6261 and UNC7394. *n* = 2 biological replicates. f) CAPA analysis of UNC6261‐CT. Data are the mean ± SEM.

As CDYL is indispensable for pain processing, pursuing chemical antagonists of CDYL may be a potential strategy for pain management. We previously reported peptidomimetic ligands with modest affinity for the CDYL chromodomain family; however, these ligands are not sufficiently potent for use in cells.^[^ ^28^, ^29^ ^]^ Through modification of these peptides at a number of positions in order to improve potency and selectivity and screening using a previously reported TR‐FRET assay for CDYL2,^[^ ^30^ ^]^ we arrived at UNC6261 which binds CDYL2 with an *IC*_50_ of 81 ± 16 nm (**Figure** **7**a and Figure S6, Supporting Information). Moreover, UNC6261 binds to the CDYL chromodomain about equipotently, yielding a *K_d_* of 139 ± 3.3 nm (Figure 7b). Encouragingly, UNC6261 is 13‐fold selective for CDYL over the closely related protein MPP8, and more than 45‐fold selective over members of the HP1 and Polycomb family of chromodomains (Figure S7, Supporting Information). A structurally similar negative control compound, UNC7394, was synthesized with no measurable binding to CDYL (Figure 7a,b).

To understand the binding mode of UNC6261, we solved the cocrystal structure of UNC6261 bound to the chromodomain of CDYL (PDB: 7N27, Table S3, Supporting Information). UNC6261 engages the chromodomain via a “surface groove” mode of binding involving the backbone amide bonds of UNC6261 (Figure 7c). Interestingly, the aromatic cage of CDYL appears to adopt a wider, less structured confirmation than other chromodomains such as MPP8, in turn accommodating the larger isopropyl/methyl‐imidazole lysine mimetic of UNC6261. The basic methyl‐imidazole substituent is also in close proximity to the aspartic acid in the aromatic cage, suggesting that potential electrostatic interactions could be contributing to the potency and selectivity of UNC6261 for CDYL.

To confirm that UNC6261 engages full‐length endogenous CDYL, cell lysates from MDA‐MB‐231 cells were incubated with DMSO, UNC6261, or UNC7394 at 100 µm overnight, followed by chemiprecipitation with a biotinylated derivative of UNC6261 (UNC6261‐Biotin, Figure 7d).^[^ ^31^ ^]^ While CDYL was effectively chemiprecipitated in the DMSO control, pretreatment with UNC6261 but not UNC7394 significantly reduced pulldown of CDYL (Figure 7e), confirming that UNC6261 engages the CDYL chromodomain in the context of full‐length protein.

A potential pitfall of peptidomimetic ligands is their poor cellular permeability. Therefore, to assess the cell permeability of UNC6261, we synthesized a chlorotagged derivative (UNC6261‐CT, Figure 7d) and performed the chloroalkane penetration assay (CAPA).^[^ ^32^, ^33^ ^]^ This revealed that UNC6261 has a *CP*_50_ of 5.5 ± 0.6 µm (Figure 7f), suggesting that concentrations above 5 µm should be used for future cellular studies to achieve a high intracellular concentration to effectively engage the target. Additionally, no toxicity was observed with UNC6261 and UNC7394 up to 100 µm using a CellTiter‐Glo assay (Figure S8, Supporting Information). Moreover, we examined the effect of UNC6261 on CDYL transcriptional activity. The mRNA levels of five CDYL target genes (*Kcnb1*, *Nsf*, *Npas4*, *Syt7*, and *Glra1*) were elevated by treatment with UNC6261 in a dose‐dependent manner (Figure S9, Supporting Information). Overall, UNC6261 is a potent CDYL antagonist that can serve as a powerful tool to assess the effects of CDYL antagonism on pain processing.

### 2.8 UNC6261 Produces Analgesia in Mice

**Figure 8:**
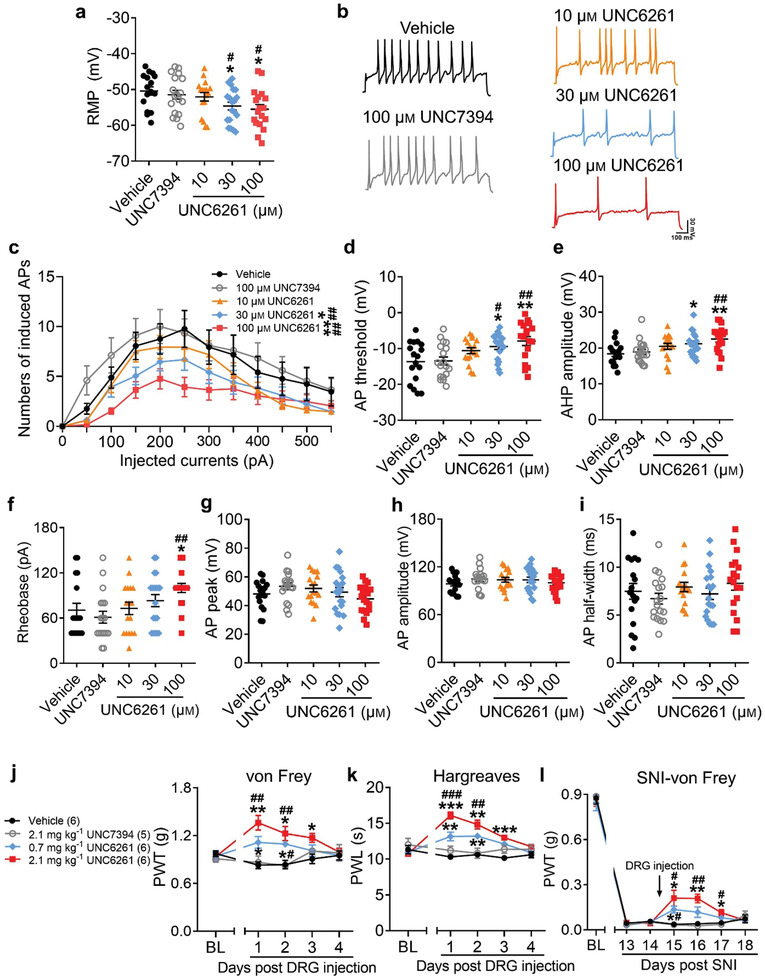
Suppressing peripheral CDYL activity by its antagonist reduces neuronal excitability and pain‐like behaviors. a) The RMP of DRG neurons treated with 10, 30, 100 µm UNC6261, 100 µm UNC7394 or vehicle for 24 h. One‐way ANOVA with Turkey's post‐hoc test, *p < 0.05 versus vehicle; ^#^ *p* < 0.05 versus UNC7394. b,c) Representative traces (b) and numbers (c) of APs induced by indicated currents. Scale bars, 100 ms and 30 mV. Two‐way ANOVA, group effect: *p < 0.05, **p < 0.01 versus vehicle; ^##^ *p* < 0.01 versus UNC7394. d–f) The AP threshold (d), AHP amplitude (e), and rheobase (f) in DRG neurons. One‐way ANOVA with Turkey's post‐hoc test, *p < 0.05, **p < 0.01 versus vehicle; ^#^ *p* < 0.05, ^##^ *p* < 0.01 versus UNC7394. g–i) The peak (g), amplitude (h), and half‐width (i) of AP in DRG neurons. One‐way ANOVA with Turkey's post‐hoc test, no statistical significance. *n*_vehicle_ = 17, *n*_UNC7394_ = 18, *n*_10 µm UNC6261_ = 17, *n*_30 µm UNC6261_ = 19, *n*_100 µm UNC6261_ = 18. j,k) Time course of PWT (j) and PWL (k) of mice after injection of 0.7 and 2.1 mg kg^−1^ UNC6261, 2.1 mg kg^−1^ UNC7394 or vehicle to DRG. Two‐way ANOVA with Sidak's post‐hoc test, *p < 0.05, **p < 0.01, ***p < 0.001 versus vehicle; ^##^ *p* < 0.01, ^###^ *p* < 0.001 versus UNC7394. l) Time course of PWT of SNI mice with injection of UNC6261, UNC7394 or vehicle. Two‐way ANOVA with Sidak's post‐hoc test, *p < 0.05, **p < 0.01 versus vehicle; ^#^ *p* < 0.05, ^##^ *p* < 0.01 versus UNC7394. Data are the mean ± SEM.

To evaluate the ability of UNC6261 to produce analgesia, DRG neurons were incubated with 10, 30, or 100 µm UNC6261, 100 µm UNC7394 or vehicle (phosphate buffered saline, PBS) for 24 h and then the whole‐cell current‐clamp was performed to test the intrinsic neuronal membrane properties. The RMP and the average numbers of evoked APs were remarkably declined in neurons treated with 30 and 100 µm UNC6261 compared to vehicle‐ or UNC7394‐treated neurons (**Figure** **8**a–c). Moreover, treatment with 30 and 100 µm UNC6261 increased the AP threshold and AHP amplitude (Figure 8d,e). In addition, 100 µm UNC6261 also increased the current threshold of DRG neurons (Figure 8f). Other AP parameters were not affected by antagonizing the CDYL chromodomain (Figure 8g–i).

Next, UNC6261 was delivered into unilateral L4–5 DRG in mice to test its effect on pain sensation. Our in vivo results showed that the basal pain threshold was increased in UNC6261‐treated mice (Figure 8j,k) while the motor function and touch sensation were unchanged (Figure S10, Supporting Information). Encouragingly, in SNI model, treatment with 0.7 or 2.1 mg kg^−1^ UNC6261 after the initiation of pain could successfully relieve mechanical hypersensitivity (Figure 8l). Collectively, peripheral treatment with a CDYL antagonist may be a promising strategy for neuropathic pain management.

## 3 Discussion

### 3.1 Peripheral CDYL is Actively Involved in Pain Modulation

The susceptibility to chronic pain is significantly different between individuals and even monozygotic twins may show inconsistent sensitivity to pain,^[^ ^34^ ^]^ indicating that both genetic and environmental factors contribute to the pain states. Increasing evidence has shown that noxious stimuli alter the expression of epigenetic regulators and relevant epigenetic modifications, resulting in the dysregulation of pain‐related genes.^[^ ^1^, ^6^, ^35^, ^36^ ^]^ For example, G9a, a histone methyltransferase, was reported to promote the downregulation of potassium channels and opioid receptors genes after nerve injury.^[^ ^37^, ^38^ ^]^ Another histone methyltransferase, EZH2, was also found to stimulate proinflammatory cytokines secretion in neuropathic pain.^[^ ^39^, ^40^ ^]^ Additionally, HDAC activity was found to decline in neuropathic pain model.^[^ ^41^ ^]^

CDYL, an epigenetic factor, has been show to recruit histone‐modifying enzymes including G9a, EZH2, and HDAC1/2 to repress gene transcription.^[^ ^10^, ^13^ ^]^ Recent studies have demonstrated that CDYL participates in the development of neuronal function and neuropsychiatric disorders through epigenetic mechanisms.^[^ ^16^, ^17^, ^42^, ^43^ ^]^ Here, we identified that CDYL expression in DRG was altered under chronic pain state, raising the possibility that peripheral CDYL may play a regulatory role in pain signaling pathways. We further found that knockdown or knockout of CDYL in DRG impaired the responses to mechanical and heat stimuli while overexpression of CDYL enhanced nociceptive responses, suggesting a pivotal role of CDYL in the transmission of nociceptive signals.

Suppression of central CDYL has been reported to induce neuronal hyperexcitability in several studies.^[^ ^16^, ^17^ ^]^ Distinct from its role in the CNS, our results showed that peripheral *Cdyl* deficiency led to the decreased excitability of sensory neurons, which was reflected in the lower RMP, higher AP and current threshold, and increased AHP amplitude. One possible explanation is that the transcriptome and epigenome profiles such as different expression pattern of ion channels between the CNS and the peripheral nerve system (PNS) are significantly different. Thus, the diversity of CDYL target genes in the CNS and PNS may complicate its regulatory role in pain processing.

### 3.2 CDYL‐K_v_2.1 Signaling Axis Plays a Key Role in Nociceptive Signaling

K_v_ channels are important regulators of neuronal excitability, required for the transduction of nociceptive signals.^[^ ^44^ ^]^ Peripheral nerve injury causes a long‐lasting reduction of K_v_ currents in sensory neurons, resulting in neuronal hyperexcitability and pain symptoms.^[^ ^38^, ^44^ ^]^ Opening of K_v_ channels or restoration of K_v_ currents leads to hyperpolarization of the cell membrane and produces antinociception.^[^ ^27^, ^44^, ^45^ ^]^

K_v_2.1 channel is a delayed rectifier potassium channel responsible for AP repolarization, hyperpolarization, and/or afterhyperpolarization.^[^ ^46^ ^]^ Previous research demonstrated that K_v_2.1 channel was decreased by traumatic nerve injury in the DRG, leading to pain hypersensitivity.^[^ ^27^, ^38^ ^]^ Restoration of K_v_2.1 current by nerve growth factor exerted neuroprotection following nerve transection.^[^ ^47^ ^]^ However, the role of K_v_2.1 channel in nociceptive processing is not fully understood. Our results show that the downregulated expression of K_v_2.1 channel is triggered by peripheral nerve injury and knocking down K_v_2.1 channel promotes nociception. Moreover, CDYL governs K_v_2.1 transcription through regulating H3K27me3 at the intron region. Our study also demonstrated that disrupting K_v_2.1 channel antagonized the impact of *Cdyl* deletion on the firing frequency and AHP amplitude of AP; however, other parameters were not altered by inhibiting the functional K_v_2.1 channel. As our ChIP‐seq data revealed that a number of CDYL target genes were associated with the regulation of membrane potential, such as *Kcnc3*, *Nsf*, *Slc12a*, and *Syt7*, they may contribute to the alteration of intrinsic membrane properties as well. Therefore, other possible mechanisms of CDYL's participation in pain cannot be ruled out and remain to be studied in the future.

### 3.3 Selective CDYL Antagonist Represents a Potential Analgesic Agent

Drugs targeting epigenetic modifications have proven to relieve pain in animal models. For instance, HDAC inhibitors such as SAHA and TSA counteracted the hypersensitivity in chronic pain;^[^ ^48^, ^49^ ^]^ DNMT inhibitors such as RG108 and zebularine also produced analgesia in animal models.^[^ ^50^, ^51^ ^]^ Given that CDYL is responsible for pain sensation as a transcriptional corepressor, targeting CDYL may be beneficial for the treatment of neuropathic pain. As a result, we developed a potent CDYL antagonist UNC6261, and peripheral utilization of UNC6261 in vivo exerted an inhibitory influence on DRG neuronal excitability and pain sensation. Notably, UNC6261 could also successfully alleviate the severity of neuropathic pain. However, further characterization of UNC6261 is still needed to assess blood‐brain barrier permeability and possible side effects. Importantly, our study proposes a peripheral restricted pharmacological approach for addressing neuropathic pain, suggesting that targeting CDYL could be a strategy for the development of new analgesics.

In summary, our findings identify a key role of peripheral CDYL‐K_v_2.1 axis in nociceptive signaling and advance our understanding of epigenetic mechanisms in neuronal function. Furthermore, our study sets the foundation for the development novel effective pain therapeutics targeting epigenetic regulators including CDYL.

## 4 Conclusion

Taken together, we prove that CDYL‐K_v_2.1 axis in peripheral sensory neurons is involved in the pain processing. CDYL downregulation in DRG acts as a brake in neuronal excitability and nociceptive signaling pathway while its overexpression facilitates pain sensation, which is associated with the regulation of CDYL on *Kcnb1* transcription through the recruitment of H3K27me3 activity. In addition, a novel CDYL antagonist UNC6261 can produce analgesic effect in neuropathic pain model. Collectively, our findings reveal a new epigenetic mechanism of peripheral CDYL in pain regulation, providing a promising avenue targeting epigenetics for clinical treatment (Figure S11, Supporting Information).

## 5 Experimental Section

### Animals

All experiments were performed according to the guidelines of the Animal Care and Use Committee of Peking University. Mice were housed with littermates (<5 in a vivarium) in temperature‐controlled rooms on a 12/12‐h light–dark cycle. Food and water were provided ad libitum. Male mice were age‐matched, and used at 8–10 weeks of age.

The conditional knockout mice lacking *Cdyl* in DRG neurons were generated. The generation of *Cdyl*‐floxp mice (termed as *Cdyl* ^F/F^ mice) had been described previously.^[^ ^16^ ^]^ The *Nav1.8‐Cre* mice and *Prrxl1‐*CreER^T2^ mice were gifts from Dr. Yong Li (Shanghai Jiao Tong University). To knock out *Cdyl* specifically in small‐sized DRG neurons (termed as *Na_v_1.8* ^Cre^ *Cdyl* ^F/F^ mice), *Cdyl* ^F/F^ mice were crossed with *Na_v_1.8‐Cre* mice.^[^ ^21^ ^]^ To conditionally knock out *Cdyl* in primary somatosensory neurons with no special preference to DRG neuronal types (termed as *Prrxl*1^CreERT2^ *Cdyl* ^F/F^mice), *Cdyl* ^F/F^ mice were crossed with *Prrxl1‐CreER* ^T2^ mice.^[^ ^22^ ^]^ Because the Cre recombinase of *Prrxl1‐*CreER^T2^ mouse line was tamoxifen‐inducible, each P30 *Prrxl*1^CreERT2^ *Cdyl* ^F/F^ mouse was administrated with 8 mg of tamoxifen per 40 g body weight for 4 consecutive days by oral gavage when needed. The mice were used for experiments 4 weeks after the drug administration. *Cdyl* conditional knockout mice were viable, fertile, and did not exhibit visible abnormalities. *Cdyl* ^F/F^ mice produced in the same litter as the *Cdyl* cKO animals were therefore included as littermate controls. C57BL/6 mice (8–10 weeks) were purchased from Charles River Laboratories (Beijing).

### Mouse Genotyping

The *Cdyl* cKO mice were identified by genotyping. DNA was prepared from 3 mm of clipped tail specimen from mice and extracted in 50 µL of DNA lysis buffer containing 0.1 mg mL^−1^ proteinase K by incubation in 55 °C for 4–6 h. Then 200 µL TE buffer (B548106, Sangon Biotech) was added to the samples and the temperature was raised to 95 °C for 10 min to inactivate proteinase K. The mouse genotype was identified by PCR on the genomic DNA with 35 cycles of 95 °C for 40 s, 58 °C for 30 s, and 72 °C for 40 s. The primers for *Cdyl^F/F^* mice were: flox forward, 5′‐ACTGATGTCTTAAGATAAGGTCCTTCTG‐3′ and flox reverse, 5′‐TGCAATGAGCTCAAACTACATGCC‐3′. The primers for *Nav1.8‐Cre* and *Prrxl1‐*CreER^T2^ mice were: Cre forward, 5′‐GCCTGGCATTACCGGTCGATGC‐3′ and Cre reverse, 5′‐TGCAATGAGCTCAAACTACATGCC‐3′.

### Plasmid Construction and Viral Production

Short hairpin RNA (shRNA) plasmids specific for mouse *Cdyl* were constructed by insertion of the following sequence into pSUPER vector: sense: 5′‐GATCCCC‐GAGATATTGTCGTCAGGAA‐TTCAAGAGATTCCTGACGACAATATCTC‐TTTTTA‐3′, antisense: 5′‐AGCTTAAAAATTCCTGACGACAATATCTC‐TCTCTTGAA‐GAGATATTGTCGTCAGGAAGGG‐3′. The sequences for scramble shRNA were constructed as follows: sense: 5′‐GATCCCC‐TTCTCCGAACGTGTCACGTTTCAAGAGA‐ACGTGACACGTTCGGAGAA‐TTTTTA‐3′, antisense: 5′‐AGCTTAAAAA‐TTCTCCGAACGTGTCACGT‐TCTCTTGAAACGTGACACGTTCGGAGAA‐GGG‐3′.^[^ ^16^ ^]^ Human CDYL was cloned into the pCAGn GS‐IRES‐EGFP vector to construct rescue plasmid.^[^ ^42^ ^]^ Adeno‐associated virus serotype 5 (AAV5) carrying shRNA‐targeting mouse K_v_2.1(EGFP‐vector) were produced by Wuhan BrainVTA. The K_v_2.1 shRNA sequence was: 5′‐GCATCGAGATGATGGACATCGTTCAAGAGACGATGTCCATCATCTCGATGCTTTTTT‐3′.

### Western Blot

Mice were deeply anesthetized by pentobarbital sodium (80 mg kg^−1^, i.p.), and DRG tissues were rapidly dissected and homogenized in ice‐cold lysis buffer (50 mm tris pH 7.4, 150 mm NaCl, 5 mm EGTA, 0.5% NP‐40, 10 mm NaF, and 1 mm PMSF). The supernatants were collected by centrifugation at 12 000*g* at 4 °C for 10 min, and protein was quantified using a BCA assay kit (Pierce). Then each sample containing 55 µg protein was denatured, subjected to SDS‐polyacrylamide gel electrophoresis and transferred onto nitrocellulose membranes. The membranes were blocked with 5% nonfat milk for 1 h at room temperature and were probed with antibodies. The antibodies against CDYL (1:1000, Proteintech, 17763‐1‐AP), K_v_2.1(1:500, Proteintech, 19963‐1‐AP), *β*‐actin (1:1000, Santa Cruz Biotechnology, sc‐47778) were used. The membranes were visualized with enhanced chemiluminescence and the intensities of specific bands were quantified and normalized to that of a loading control band.

### Immunofluorescence Staining

Mice were perfused with 4% paraformaldehyde (PFA) and L4–5 DRG neurons were fixed in 4% PFA at 4 °C for 6 h. Fixed DRG were dehydrated in 20–30% (w/v) sucrose for cryoprotection and embedded in Tissue‐Tek O.C.T. compound (#4583, SAKURA). DRG neurons were sectioned with a cryostat at 8‐µm thickness. Sections were washed with phosphate‐buffered saline (PBS, 137 mm NaCl, 2.7 mm KCl, 10 mm NaH_2_PO4, 2 mm K_2_HPO4, pH 7.4) once for 20 min and blocked for 1 h at room temperature with PBS containing 0.1% Triton X‐100 and 3% bovine serum albumin (BSA). Then sections were incubated with primary antibodies against CDYL (1:250, Sigma‐Aldrich, HPA035578), NF200 (1:200, Sigma‐Aldrich, N0142), CGRP (1:150, Abcam, ab81887) at 4 °C overnight. Next, sections were washed with PBS three times for 10 min each and incubated with appropriate secondary antibodies (Alexa Fluor 488‐conjugated donkey anti‐mouse, Invitrogen, 1: 1000, A‐21202; Alexa Fluor 594‐conjugated donkey anti‐rabbit, Invitrogen, 1: 1000, A‐2120), or IB4‐FITC (1:50, Sigma‐Aldrich, L2895) at 4 °C overnight. Finally, sections were stained with Hoechst (1:500, Solarbio, C0031) and coverslips were applied. Images were acquired using a confocal laser‐scanning microscope (Leica TCS SP8 STED). The DRG neurons with clear nuclear morphology neuronal areas were quantified using Image‐Pro Plus 6.0 software.

### ChIP and ChIP‐qPCR

ChIP was performed using SimpleChIP Plus Enzymatic Chromatin IP Kit (#9005, Cell Signaling), following the manufacturer's instructions. Briefly, the cells were collected and cross‐linked with 1.5% formaldehyde for 20 min. After cross‐linking, DRG tissues were disaggregated into a single‐cell suspension. Cells were then lysed and the chromatin was fragmented by micrococcal nuclease to obtain chromatin fragments of 150–500 bp. Then the chromatin samples were incubated with ChIP‐grade protein G magnetic beads and antibodies against CDYL (1:100, HPA035578, Sigma), H3K27me3 (1:200, A2363, Abclonal), H3K9me2(1:200, A2359, Abclonal), H3K9me3 (1:500, A2360, Abclonal), H3K27ac (1:500, A7253, Abclonal). After reversal of protein‐DNA cross‐links, the DNA was purified using DNA purification spin columns provided in the kit. The enrichment of precipitated DNA sequences was analyzed by real‐time PCR using primers listed in Table S1, Supporting Information.

### ChIP Sequencing

The chromatin DNA precipitated by specific antibody against mouse CDYL or IgG was purified. ChIP‐sequencing was performed by Berry Genomics Corporation, Beijing. Enriched DNA sequencing were performed on the Illumina Novaseq6000, mapped to the mouse genome (GRCm38, mm10) using bowtie software, and the peaks were called by MACS. Enriched binding peaks of CDYL were generated after filtering through the control IgG. The promoter region was defined as ≤+2 kb from the transcription start site.

### Quantitative Real Time Reverse Transcription (RT)‐PCR Assay

Total RNA was extracted from DRG tissues using RNA isolation kit (#RN07, Aidlab), and reverse‐transcribed to cDNA using RT Master Mix (#G490, Abmart). Real‐time PCR was performed using SYBR‐Green Master mix (#QPK‐201, Toyobo) on an Applied Biosystems 7500 apparatus. The relative quantification of target gene expression was performed with the comparative cycle threshold method with normalized to the level of a housekeeping gene, Gapdh. The sequences of the primers used are provided in Table S2, Supporting Information.

### Intrathecal Injection

The mice were anesthetized with 2% isoflurane. After shaving and sterilization, the mice were gripped and 10 µL Hamilton syringe with 27G needle was inserted into one side of the L5 spinous process so that it slipped into the groove between the spinous and transverse processes. 5 µL solution was injected slowly following a sudden tail flick.

### DRG Microinjection

Adult male mice received microinjection with AAV5 vectors expressing K_v_2.1 shRNA into unilateral L4–5 DRG. AAV5‐scramble virus served as the controls. DRG microinjection was carried out as described.^[^ ^52^ ^]^ Briefly, unilateral L4 DRG was exposed and micro syringe pump with glass electrode was adjusted at 45° inserting into DRG with depth ≈0.2–0.3 mm. 500 nL of viral solution (titer ≥ 1 × 10^12^ mL^−1^) was injected into the DRG at a rate of 50 nL min^−1^. The glass electrode was left in place for 5 min after the injection.

To assess the effect of focal application of CDYL inhibitor to DRG, 500 nL of UNC6261 solution (at 100 mm resolved in PBS) were delivered using DRG microinjection. Mice were left to recover for at least 24 h before the experiments were carried out.

### Dissociation of DRG Neurons

The dissociation of DRG neurons was performed as previously described.^[^ ^19^ ^]^ Briefly, DRG tissues were quickly dissected out and digested with 3 mg mL^−1^ collagenase type IA (Sigma‐Aldrich) for 38 min and with 0.25% trypsin (Sigma‐Aldrich) for 6.5 min at 37 °C. Enzymatic treatment was terminated by the addition of Dulbecco's modified Eagle's medium (DMEM) containing fetal bovine serum (FBS) followed by gentle trituration of the ganglia with a flame‐polished Pasteur pipette and centrifugation at 1000 rpm for 3 min. Then the cell pellet was resuspended in DMEM with 10% FBS and plated on poly‐d‐lysine‐coated (Sigma‐Aldrich) culture dishes. The cells were incubated at 37 °C with 5% CO_2_ and 95% air.

### Whole‐Cell Patch Clamp Recordings

The current clamp recording was performed to record AP 2 to 6 h after DRG neurons plating using EPC‐10 amplifier and Pulse software (HEKA Instruments). The intracellular solution contained (in mm) 140 K‐gluconate, 10 NaCl, 5 EGTA, 1 CaCl_2_, 10 HEPES, 2 Mg‐ATP, 0.2 Na‐ATP (pH 7.3 adjusted with NaOH, 300 mOsm). The extracellular solution contained (in mm) 129 NaCl, 5 KCl, 2 CaCl_2_, 1 MgCl_2_, 25 HEPES, 30 Glucose (pH 7.4 adjusted with KOH, 310 mOsm). DRG neurons were examined for evoked AP with a series of 1‐s current injections from 0 to 580 pA in 20 pA increments. The following values were measured in this study: RMP, AP threshold, rheobase current, evoked AP numbers, AP amplitude, AP peak, AP half‐width, and AHP amplitude. RMP was measured 2 min after a stable recording was obtained. The rheobase current was defined as the minimum current sufficient to evoke an AP in 20 ms. AP threshold was defined as the first point on the rising phase of an AP where depolarization was greater than 50 mV ms^−1^.^[^ ^53^ ^]^ The AHP amplitude was measured between the maximum hyperpolarization and the final plateau voltage.^[^ ^6^ ^]^ The DRG neurons that had stable membrane potentials more negative than −40 mV were included for further analysis.^[^ ^6^ ^]^ The data were analyzed by the pCLAMP 10.0 software package (Molecular Devices). All experiments were performed at room temperature.

### Potassium Channel Current Recordings

The K_v_ currents of dissociated DRG neurons from 6–8 weeks mice were recording as previously described.^[^ ^19^, ^53^ ^]^ The intracellular pipette solution contained (in mm) 140 K aspartate, 13.5 NaCl, 1.6 MgCl_2_, 0.09 EGTA, 9 HEPES, 14 creatine phosphate (tris salt), 4 Mg‐ATP, 0.3 tris‐GTP (pH 7.2 adjusted with KOH, 297 mOsm). The extracellular solution contained (in mm) 155 NaCl, 3.5 KCl, 1.5 CaCl_2_, 1 MgCl_2_, 10 HEPES, 10 glucose (pH 7.4 adjusted with NaOH, 310 mOsm). TTX (1 mm), A‐803467 (1 mm) were applied to inhibit sodium channels. The K_v_ currents were elicited by 400‐ms depolarizing pulses between −70 and +60 mV from a holding potential of −80 mV, followed by a 1‐s pulse at −40 mV. K_v_2.1 current were obtained by subtracting the K_v_ currents recorded in the presence of the specific K_v_2.1 channel blocker, 100 nm GxTX‐1E (Abcam, ab141872), from the baseline total K_v_ currents.

### Behavioral Tests—Open Field Test

The apparatus was a white plastic open field with a square floor (60 cm × 60 cm) and surrounding walls (100 cm high). The overall illumination was kept at 100–200 lux. Each mouse was gently placed in the center of the open field and videotaped for 30 min.^[^ ^16^ ^]^ The total distance mice travelled were measured using the software SMART (version 2.5.21, PanLab, Barcelona, Spain).

### Rotarod Test

To evaluate sensorimotor coordination, rotarod test was performed as described previously.^[^ ^54^ ^]^ In the training session, mice were placed on a rotarod moving at 5 rpm for 5 min. Mice were trained to stay on the rotarod for the entire 5 min. If a mouse fell, it was placed back on the rotarod and the 5 min trial began again. Training were carried out on 2 consecutive days. On the third day, mice ran their full rotarod test. The rotarod began at 5 rpm and accelerated to 40 rpm over 5 min. The latency to falling off was recorded. The examination was repeated three times with 15‐min interval.

### Hot Plate Test

To investigate thermal pain, mice were placed on the hot plate (IITC, Harvard) set at 50, 52, or 54 °C and the latency to hind paw licking was recorded. The trial was repeated three times with 15‐min interval. To avoid tissue injury, a cut‐off time was set at 30 s.

### Radiant Heat Test

Mice were put in plastic chambers and habituated for 20 min. The radiant heat intensity was adjusted to a range of 10–12 s for control mice as the baseline latency with a cut‐off time of 20 s to avoid tissue damage. To record the paw withdrawal latency, each hind paw was measured five times at 10‐min interval, and the average was calculated.

### Von Frey Test

To measure the mechanical paw withdrawal threshold, mice were placed in a plastic cage with a metal mesh floor, and stroked the plantar surface of hind paw using von Frey filaments (Stoelting) ranging from 0.023 to 2.042 g. Each filament was applied to the hind paw until it bent and was kept in this position for 4–5 s. The mechanical threshold was determined by Dixon's up‐and‐down method provided in previous studies.^[^ ^55^ ^]^

### Pinprick Test

For pinprick test, the plantar surface of the hind paw was touched with a pin glued onto an 1 g von Frey filament without skin penetration, and measured the quantity of withdrawal response per ten trials with an interval of 1 min.^[^ ^54^ ^]^

### Pinch Test

For pinch test, an alligator clip was put onto the ventral skin surface between the footpad and the heel. Then the mice were placed in a plexiglass chamber placed onto a glass, allowing video to record for 60 s to measure the duration and numbers of licking and flinching.^[^ ^54^ ^]^

### Acetone Evaporation Test

To measure evaporative cooling sensation, mice were placed in an elevated chamber with a mesh floor. Then a small drop of acetone was sprayed to the plantar hind paw using a syringe and scored the response. The trials were repeated five times and the average scores were obtained. Behaviors were scored according to the magnitude of the response along the following scales: 0 = no response; 1 = brief lift, sniff, flick, or startle; 2 = jumping, paw shaking; 3 = multiple lifts, paw lick; 4 = prolonged paw lifting, licking, shaking, or jumping; 5 = paw guarding.^[^ ^54^ ^]^

### Brush Test

To measure dynamic allodynia, a paint brush was used to stimulate the plantar hind paw lightly in the heel‐to‐toe direction and repeated three times with 1‐min interval to obtain the average score for each mouse. The score was recorded as previously described.^[^ ^54^ ^]^ For each test under basal conditions, 0 = no response; 1 = occasionally very brief paw lifting; 2 = flicking of the paw; 3 = flinching or licking of the paw. For each test in CFA model, 0 = walking away or occasionally very brief paw lifting; 1 = a sustained lifting (more than 2 s) of the stimulated paw toward the body; 2 = a strong lateral lifting above the level of the body; 3 = flinching or licking of the affected paw.

### Cotton Swab Test

To complement the light touch behavioral assay, a light punctate force assay was performed as previously described.^[^ ^56^ ^]^ Briefly, a cotton swab was puffed out such that the cotton head was three times its normal size. The hind paw of mice briefly was stroked for ten times using the cotton swab in the heel‐to‐toe direction with 1‐min interval and recorded the frequency of responses.

### Flick Hairy Test

To assess touch‐evoked scratching response, the flick hairy test was performed as previously described.^[^ ^56^ ^]^ Briefly, hair of the neck of the mice was shaved and placed into the mesh‐floor chamber. A 0.07 g von Frey filament was used to stimulate the shaved skin for ten times, and the percentage of scratching response was noted.

### Sticky Tape Test

For sticky tape test, a 1 × 0.5 cm rectangular adhesive sticky tape (Comix) was put on the hind paw plantar of mice and measured the latency of biting or licking to remove the tape.^[^ ^54^ ^]^

### Protein Expression and Purification for Biochemical Assays

The chromodomain of CDYL (residues 1–78 of NP_004815) was expressed with an N‐terminal GST‐tag in a pGEX4T expression vector. The expression construct was transformed into Rosetta2 BL21(DE3)pLysS competent cells (Novagen, EMD Chemicals, San Diego, CA). Protein expression was induced by growing cells at 37 °C with shaking until the OD_600_ reached ≈0.6–0.8 at which time the temperature was lowered to 18 °C and expression was induced by adding 0.5 mm IPTG and continuing shaking overnight. Cells were harvested by centrifugation and pellets were stored at −80 °C.

GST‐tagged CDYL was purified by resuspending thawed cell pellets in 30 mL of lysis buffer (1× PBS, 5 mm dithiothreitol (DTT), 1× EDTA free protease inhibitor cocktail (Roche Diagnostics, Indianapolis, IN) per liter of culture. Cells were lysed on ice by sonication and the clarified cell lysate was loaded onto a GSTrap FF column (GE Healthcare, Piscataway, NJ) that had been pre‐equilibrated with ten column volumes of binding buffer (1× PBS, 5 mm DTT) using a AKTA FPLC (GE Healthcare, Piscataway, NJ). The column was washed with ten column volumes of binding buffer and protein was eluted in 100% elution buffer (50 mm tris pH 7.5, 150 mm NaCl, 10 mm reduced glutathione) over ten column volumes. Peak fractions containing the desired protein were pooled and concentrated to 2 mL in Amicon Ultra‐15 concentrators, 10 000 molecular weight cut‐off (Merck Millipore, Carrigtwohill Co. Cork IRL). Concentrated protein was loaded onto a HiLoad 26/60 Superdex 200 prep grade column (GE Healthcare, Piscataway, NJ) that had been pre‐equilibrated with 1.2 column volumes of sizing buffer (25 mm tris pH 7.5, 250 mm NaCl, 2 mm DTT, 5% glycerol) using an ATKA FPLC (GE Healthcare, Piscataway, NJ). Protein was eluted isocratically in sizing buffer over 1.3 column volumes at a flow rate of 2 mL min^−1^ collecting 3 mL fractions. Peak fractions were analyzed for purity by SDS‐PAGE and those containing pure protein were pooled and concentrated using Amicon Ultra‐15 concentrators 10 000 molecular weight cut‐off (Merck Millipore, Carrigtwohill Co. Cork IRL).

The chromodomain of CBX1 (residues 20–73 of NP_006798), CBX5 (residues 18–75 of NP_036429), and MPP8 (residues 55–116 of NP_059990) were expressed with N‐terminal His‐tags in pET28 expression vectors. The chromodomain of CBX2 (residues 9–66 of NP_001580), CBX7 (residues 8–62 of NP_783640), and CDYL2 (residues 1–75 of NP_689555) were expressed with C‐terminal His‐tags in pET30 expression vectors. All expression constructs were transformed into Rosetta2 BL21(DE3)pLysS competent cells (Novagen, EMD Chemicals, San Diego, CA). Protein expression was induced by growing cells at 37 °C with shaking until the OD600 reached ≈0.6–0.8 at which time the temperature was lowered to 18 °C and expression was induced by adding 0.5 mm IPTG and continuing shaking overnight. Cells were harvested by centrifugation and pellets were stored at −80 °C.

His‐tagged proteins were purified by resuspending thawed cell pellets in 30 mL of lysis buffer (50 mm sodium phosphate pH 7.2, 50 mm NaCl, 30 mm imidazole, 1× EDTA free protease inhibitor cocktail (Roche Diagnostics, Indianapolis, IN) per liter of culture. Cells were lysed on ice by sonication with a Branson Digital 450 Sonifier (Branson Ultrasonics, Danbury, CT) at 40% amplitude for 12 cycles with each cycle consisting of a 20‐s pulse followed by a 40‐s rest. The cell lysate was clarified by centrifugation and loaded onto a HisTrap FF column (GE Healthcare, Piscataway, NJ) that had been pre‐equilibrated with 10 column volumes of binding buffer (50 mm sodium phosphate pH 7.2, 500 mm NaCl, 30 mm imidazole) using an AKTA FPLC (GE Healthcare, Piscataway, NJ). The column was washed with 15 column volumes of binding buffer and protein was eluted in a linear gradient to 100% elution buffer (50 mm sodium phosphate pH 7.2, 500 mm NaCl, 500 mm imidazole) over 20 column volumes. Peak fractions containing the desired protein were pooled and concentrated to 2 mL in Amicon Ultra‐15 concentrators 3000 molecular weight cut‐off (Merck Millipore, Carrigtwohill Co. Cork IRL). Concentrated protein was loaded onto a HiLoad 26/60 Superdex 75 prep grade column (GE Healthcare, Piscataway, NJ) that had been pre‐equilibrated with 1.2 column volumes of sizing buffer (25 mm tris pH 7.5, 250 mm NaCl, 2 mm DTT, 5% glycerol) using an ATKA Purifier (GE Healthcare, Piscataway, NJ). Protein was eluted isocratically in sizing buffer over 1.3 column volumes at a flow rate of 2 mL min^−1^ collecting 3 mL fractions. Peak fractions were analyzed for purity by SDS‐PAGE and those containing pure protein were pooled and concentrated using Amicon Ultra‐15 concentrators 3000 molecular weight cut‐off (Merck Millipore, Carrigtwohill Co. Cork IRL).

The N‐terminal GST‐tag was removed from CDYL by thrombin cleavage according to manufacturer's recommendations (Novagen, EMD Chemicals, San Diego, CA). Briefly, purified protein was incubated with biotinylated thrombin at a final concentration of 1 unit thrombin per milligram tagged protein for 16 h at 4 °C. The cleavage reaction was then passed over a GSTrap FF column, as previously described, to remove any protein that still retained the tag. The column flow through was collected and concentrated to 2 mL in Amicon Ultra‐15 concentrators 3000 molecular weight cut‐off (Merck Millipore, Carrigtwohill Co. Cork IRL). Concentrated protein was loaded onto a HiLoad 26/60 Superdex 75 prep grade column (GE Healthcare, Piscataway, NJ) that had been pre‐equilibrated with 1.2 column volumes of sizing buffer (25 mm tris pH 7.5, 250 mm NaCl, 2 mm DTT, 5% glycerol) using an ATKA Purifier (GE Healthcare, Piscataway, NJ). Protein was eluted isocratically in sizing buffer over 1.3 column volumes at a flow rate of 2 mL min^−1^ collecting 3 mL fractions. Peak fractions were analyzed for purity by SDS‐PAGE and those containing pure protein were pooled and concentrated using Amicon Ultra‐15 concentrators 3000 molecular weight cut‐off (Merck Millipore, Carrigtwohill Co. Cork IRL).

Protein was exchanged into a buffer containing 25 mm tris pH 7.5, 150 mm NaCl, 2 mm *β*‐mercaptoethanol prior to use in ITC.

### Isothermal Titration Calorimetry

ITC measurements were recorded at 25 °C using an AutoITC200 microcalorimeter (MicroCal Inc., MA). Protein was dialyzed into ITC buffer (25 mm tris‐HCl, pH 8, 150 mm NaCl, and 2 mm *β*‐mercaptoethanol) and then diluted into ITC buffer to achieve a final concentration of 100 *μ* m (325 µL). Peptides were dissolved in ITC buffer at a concentration of 10 mm and then diluted to the final concentration of 1 mm. Protein concentrations were tenfold lower than the concentration of peptide. A typical experiment included a single 0.2 µL compound injection into a 200 µL cell filled with protein, followed by 26 subsequent 1.5 µL injections of compound. Injections were performed with a spacing of 180 s and a reference power of 8 *μ*cal s^−1^. The titration data was analyzed using Origin Software (MicroCal Inc., USA) by nonlinear least‐squares, fitting the heats of binding as a function of the compound:protein ratio to a one site binding model. The first data point was deleted from all analyses. All assays were run in duplicate or triplicate. The data was fit separately for each experiment and the reported *K*_d_ was the average of all of the runs. Error was calculated as the standard deviation of the various *K*_d_ values.

### Time‐Resolved Fluorescence Energy Transfer Assay

The TR‐FRET assay was performed as previously reported.^[^ ^30^ ^]^ Briefly, the assay was completed using Kme reader buffer containing 20 mm tris pH 7.5, 150 mm NaCl, 0.05% Tween 20, and 2 mm DTT. White, low‐volume, flat‐bottom, nonbinding, 384‐well microplates (Greiner, #784 904) were used for screening with a total assay volume of 10 µL. 384‐well, V‐bottom polypropylene plates (Greiner, #781 280) were used for compound serial dilutions and for transfer of assay mixtures. For compounds stored in DMSO, serial dilutions were made using DMSO. Following addition of all assay components, plates were sealed with clear covers, gently mixed on a tabletop shaker for 1 min, centrifuged at 1000 g for 2 min, and allowed to equilibrate in a dark space for 1 h before reading. Measurements were taken on an EnVision 2103 Multilabel Plate Reader (Perkin Elmer) using an excitation filter at 320 nm and emission filters at 615 and 665 nm. 615 and 650 nm emission signals were measured simultaneously using a dual mirror at D400/D630. TR‐FRET output signal was expressed as emission ratios of acceptor/donor (665 nm/615 nm) counts. Percent inhibition was calculated on a scale of 0% (i.e., activity with DMSO vehicle only) to 100% using full column controls on each plate. The interquartile mean of control wells was used to calculate *Z*′ values. For dose‐response curves, data was fit with a four‐parameter nonlinear regression analysis using GraphPad Prism 7.0 or ScreenAble software to obtain *IC*_50_ values.

### Cell Culture and Lysis

MDA‐MB‐231 cells were obtained through the ATCC (HTB‐26). Cells were cultured in DMEM high glucose (Gibco, 11995‐065), 1% pen/strep, 1% NEAA, and 10% FBS. All cells were maintained in a humidified incubator at 37 °C, 5% CO_2_. Cells were harvested by scraping and the cell pellets washed two times with PBS. Cells (≈30 million) were suspended in 500 µL of CytoBuster protein extraction reagent (Millipore Sigma) containing 5 µL of 100× protease inhibitor cocktail and 1 µL of Benzonase (≥250 units µL^−1^, Millipore Sigma) and incubated in a water bath at 37 °C for 10 min. The samples were then rotated at room temperature for 20 min followed by centrifugation for 1 min at 14 000 rpm and the supernatant was removed. Total protein was quantified using Bio‐Rad protein assay by generating a standard curve from stock BSA solutions.

HeLa cells stably expressing the HaloTag‐GFP‐mitochondria construct were provided by the Kritzer lab. Cells were cultured in DMEM high glucose media (Sigma) supplemented with 10% FBS (VWR Life Sciences Seradigm), 1% Penicillin–Streptomycin (Sigma), and 1 µg mL^−1^ Puromycin (InvivoGen) to select for Halo‐Tag expressing populations and incubated at 37 °C with 5% CO_2_.

U2OS cells were obtained from ATCC (HTB‐96). Cells were cultured in RPMI 1640 Media (Gibco) 10% FBS (VWR Life Sciences Seradigm), 1% Penicillin–Streptomycin (Sigma) and incubated at 37 °C with 5% CO_2_. Cells were harvested using 0.25% Trypsin (Gibco) for 5 min at room temperature.

### Chemiprecipitation Experiments with UNC6261‐Biotin Followed by Western Blot

1 mg of cell lysate generated from MDA‐MB‐231 cells as described above was diluted to 500 µL in TBST in the presence or absence of UNC6261 or UNC7394 and allowed to spin at 4 °C overnight. Magnetic streptavidin M‐280 Dynabeads (30 µL beads per pulldown) were incubated with UNC6261‐Biotin (1 µL of 10 mm stock) in TBST. The beads were left to rotate for 45 min at room temperature. Next, the Dynabeads were washed with TBST to remove excess biotin ligand and then added to the lysate solution. The mixture was rotated at 4 °C overnight, and then the beads were isolated by magnetization and washed with TBST. Beads were then resuspended with 15 µL TBST and 15 µL 2× LaemmLi sample buffer and heated at 95 °C for 5 min. In parallel, input of samples 10–15 µg of cell lysate was diluted to 7.5 µL and 2× Laemmi sample buffer was added and heated at 95 °C for 5 min. Half of each pull down sample was used for analysis by SDS‐PAGE (BioRad any kD) and Western blotting. Following membrane transfer, membranes were incubated with primary antibody against CDYL (1:1000, Abcam ab5188) at 4 °C overnight, washed with TBST, and then treated with the complimentary secondary antibody‐IRDye conjugate (Li‐COR; 1:10000 TBST) for 1 h at room temperature and visualized on a Li‐COR Odyssey instrument.

### Chloroalkane Penetration Assay

CAPA was performed as previously described with some modifications.^[^ ^32^ ^]^ HaloTag‐GFP HeLa cells were seeded at 5000 cells per well on a 384‐well plate and allowed to adhere overnight. On the day of the experiment, compound was prepared in a separate 384‐well plate at a final 1× concentration and total volume of 60 µL per well. Twenty concentration points were generated by performing a threefold serial dilution of a 10 mm compound water‐based stock into HeLa media. Compound‐free control wells were also prepared to be used as no‐pulse (100% signal) and no‐pulse/no‐chase (0% signal) controls. The media of the 384‐well assay plate containing cells was then removed and 50 µL of the compound samples from the dilution plate were added to each well. The plate was incubated for 4 h at 37 °C with 5% CO_2_. The media was then removed, and the cells were washed with phenol red‐free Opti‐MEM (Gibco) and incubated for 30 min. The media was then removed, and compound and no‐pulse control wells were chased with 5 µm ct‐TAMRA—synthesized according to literature procedures^1^ and prepared in phenol red‐free Opti‐MEM, and incubated for 30 min. The no‐pulse/no‐chase control wells were washed with phenol red‐free Opti‐MEM instead. The media was then removed, and the wells were washed with phenol red‐free Opti‐MEM media supplemented with 10% FBS and 1% Penicillin–Streptomycin and incubated for 15 min. The media was then removed, and the wells were washed with PBS (Corning). The media was then removed, and the cells were trypsinized with phenol red‐free 0.5% trypsin‐EDTA (Gibco) diluted 1:1 with PBS. After a 20 min incubation, the cells were quenched and resuspended with 50% FBS in PBS and analyzed by flow cytometry (iQue Screener PLUS, Intellicyt).

Live, single cells were gated first for GFP expression, and GFP positive cells were then analyzed for mean fluorescence intensity of ct‐TAMRA dye. Mean red fluorescence was normalized to the no‐pulse/no‐chase (0%) and no‐pulse (100%) signals from control wells to generate cell penetration dose response curves.

For every independent experiment, three technical replicates were performed simultaneously for each compound. The normalized fluorescence signals (see description of normalization process above) for each compound technical replicate was plotted against the log of the dosing concentration. GraphPad Prism 8 was used to fit the dose response curves using the “log (inhibitor) versus response—variable slope (four parameters)” model. *CP*_50_ values generated from the antilog of the fitted log*IC*_50_ values were recorded for each compound for every independent experiment. The *CP*_50_ values were averaged across the independent experiments, and the standard error of the mean (SEM or $\sigma_{\bar{x}}$) was determined by computing the following equation

(1) $$\sigma_{\bar{x}}=\frac{\sigma }{\sqrt{N}}$$

where *σ* is the standard deviation and *N* is the number of replicates or independent experiments.

### CellTiter‐Glo Viability Assay

The effect of UNC6261 and UNC7394 on cell viability was determined using CellTiter‐Glo (Promega #7573). Compound stocks at 50 mm in DMSO were diluted to 500 µm (or 5×) in PBS, yielding a DMSO concentration of 1%. A threefold dilution series of ten total points was then generated in PBS + 1% DMSO. 5 µL of each 5× compound stock was plated in assay wells on a Corning 384‐well, white‐walled, clear‐bottom, cell culture treated assay plate in technical triplicate. U2OS Cells were harvested, counted, and diluted to a density of 5000 cells per 20 µL. 20 µL of cell suspension per well was added on top of preplated 5× compound stocks to generate 1× concentrations (0.2% DMSO). The assay plate was centrifuged for 30 s and then incubated for 48 h at 37 °C and 5% CO_2_. Wells without cells (media only) were included as negative controls. Following incubation at 37 °C, the assay plate was equilibrated at room temperature along with the CellTiter‐Glo reagent. 25 µL of CellTiter‐Glo reagent was added to appropriate wells and the plate was centrifuged for 30 s. The assay plate was then placed on a plate shaker at room temperature for 2 min, and then allowed to equilibrate on a bench top for an additional 10 min. Luminescence was read on a PerkinElmer EnSpire Alpha Multimode Plate Reader.

### Cloning, Protein Expression, Purification, and Crystallization

The DNA fragment CDYL (aa 62‐116) was cloned into a pGEX‐MHL vector. Recombinant protein expression was induced by 0.25 mm IPTG at 16 °C overnight. The proteins were purified by GST column, and GST‐tag was removed by TEV protease. CDYL (aa 62‐116) was further purified by HiTrap Q HP (5 mL) anion exchange chromatography and size exclusion chromatography in a final buffer: 25 mm tris‐HCl, 150 mm NaCl. The protein was concentrated to 9 mg mL^−1^ and mixed with twofold compound UNC6261. The crystallization plate was set by using sitting drop vapor diffusion method by mixing 1 µL protein‐compound mixture with 1 µL reservoir solution. The crystal of CDYL in complex with UNC6261 was obtained at 18 °C in 3.5 Na Form and 0.1 bis–tris propane, pH 7.0.

### Data Collection and Structure Determination

X‐ray diffraction data for CDYL with UNC6261 was collected on the 24ID‐E beamline of advanced photon source, Argonne National Laboratory and the data was processed using the HKL‐3000 suite.^[^ ^57^ ^]^ The structures of and CDYL with compound UNC6261 were solved by molecular replacement using PHASER with PDB entry 6V2H and as search template.^[^ ^58^ ^]^ REFMAC was used for structure refinement.^[^ ^59^ ^]^ Geometry restraints for compound refinement were prepared with by PRODRG.^[^ ^60^ ^]^ Graphics program COOT was used for both model building and visualization.^[^ ^61^ ^]^ MOLPROBITY was used for structure validation.^[^ ^62^ ^]^

### General Chemistry Procedures

**Scheme 1:**
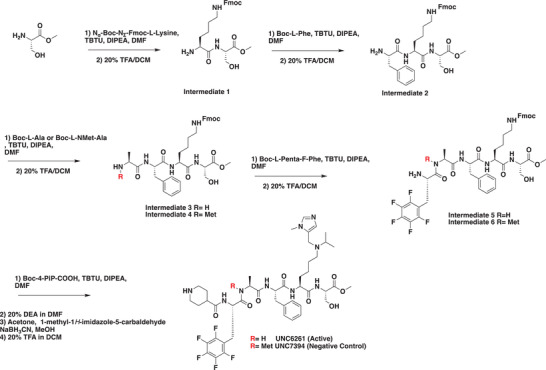
Synthesis of UNC6261 and UNC7934.

All LC‐MS were obtained on an Agilent 6110 Series LCMS with a UV detector set to 220 and 254 nm and a single quadrupole mass spectrometer. LCMS samples were run on an analytical Agilent Eclipse Plus 4.6 × 50 mm, 1.8 µm, C18 column at room temperature with mobile phases A (H_2_O + 0.1% acetic acid) and B (MeOH + 0.1% acetic acid or MeCN + 0.1% acetic acid). Mass spectra (MS) data were acquired in positive ion mode using an Agilent 6110 single quadrupole mass spectrometer with an electrospray ionization (ESI) source. Nuclear magnetic resonance (NMR) spectra were recorded on a Bruker AV at 400 MHz for proton (^1^H NMR); chemical shifts are reported in ppm (*δ*) relative to residual protons in deuterated solvent peaks. Normal phase column chromatography was performed with a Teledyne Isco CombiFlash *R*f using silica RediSep *R*f columns with the UV detector set to 220 and 254 nm. A linear mobile phase of A (DCM) and B (MeOH) up to between 15–25% B was used to purify all Boc‐protected intermediates. Reverse phase column chromatography was performed with a Teledyne Isco CombiFlash *R*f 200 using C18 RediSep *R*f Gold columns with the UV detector set to 220 and 254 nm. Mobile phases of A (H_2_O + 0.1% TFA) and B (MeOH) were used with default gradients (10–100% B). Preparative HPLC was performed using an Agilent Prep 1200 series with the UV detector set to 220 and 254 nm. Samples were injected onto a Phenomenex Luna 250 75 × 30 mm, 5 µm, C18 column at room temperature. Mobile phases of A (H_2_O + 0.1% TFA) and B (MeOH or MeCN) were used with a flow rate of 40 mL min^−1^. A general gradient of 0–15 min increasing from 10% to 100% B, followed by a 100% B flush for another 5 min. Small variations in this purification method were made as needed to achieve ideal separation for each compound. Analytical LCMS (at 220 nm) and NMR were used to establish the purity of targeted compounds. All compounds that were evaluated in biochemical and biophysical assays had >95% purity as determined by ^1^HNMR and LC‐MS (**Scheme** **1**).

### Chemistry Schemes and Experimental Procedures

Intermediate 1


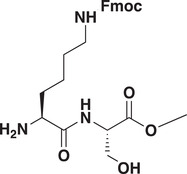


Methyl *N* ^6^‐(((9*H*‐fluoren‐9‐yl)methoxy)carbonyl)‐l‐lysyl‐l‐serinate: To a round bottom flask was added *N* ^6^‐(((9*H*‐fluoren‐9‐yl)methoxy)carbonyl)‐*N* ^2^‐(*tert*‐butoxycarbonyl)‐l‐lysine (1 g, 1.1 equiv., 2 mmol) and 2‐(1*H*‐benzo[d][1,2,3]triazol‐1‐yl)‐1,1,3,3‐tetramethyluronium tetrafluoroborate (0.7 g, 1.2 equiv., 2 mmol) followed by the addition of *N*,*N*‐dimethylformamide (20 mL) and DIPEA (0.8 g, 1.2 mL, 3 equiv., 6 mmol). The mixture stirred for 15 min followed by the addition of methyl *N* ^6^‐(((9*H*‐fluoren‐9‐yl)methoxy)carbonyl)‐*N^2^*‐(*tert*‐butoxycarbonyl)‐l‐lysyl‐l‐serinate (850 mg, 1.0 equiv., 2 mmol,). The reaction was stirred overnight followed by the addition of 100 mL of ethyl acetate and washed three times with brine and the organic phase was concentrated under reduced pressure and purified by flash chromatography (DCM/MeOH). To the obtained product was added 20% TFA in DCM and the reaction was stirred overnight at room temperature followed by purification by reverse phase flash chromatography (H_2_O + 0.1% TFA: MeOH) to yield the title compound as a TFA salt (0.96 g; 97%). ^1^H NMR (400 MHz, Methanol‐*d*_4_) *δ* 7.77 (d, *J* = 7.7 Hz, 2H), 7.62 (d, *J* = 7.5 Hz, 2H), 7.42–7.18 (m, 4H), 4.58 (t, 1H), 4.39–4.29 (m, 2H), 4.20–4.13 (m, 1H), 3.93 (dd, *J* = 11.2, 5.2 Hz, 2H), 3.83 (dd, *J* = 11.3, 3.9 Hz, 1H), 3.70 (s, 3H), 3.13 (t, *J* = 6.7 Hz, 2H), 2.02–1.71 (m, 2H), 1.64–1.30 (m, 4H). MSI (ESI): 470 [M+H]^+^. *t*_R_ = 4.14 min.

Intermediate 2


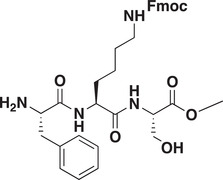


Methyl *N* ^6^‐(((9*H*‐fluoren‐9‐yl)methoxy)carbonyl)‐*N* ^2^‐(l‐phenylalanyl)‐l‐lysyl‐l‐serinate: To a round bottom flask was added (*tert*‐butoxycarbonyl)‐l‐phenylalanine (336 mg, 0.9 equiv., 1.26 mmol) and 2‐(1*H*‐benzo[d][1,2,3]triazol‐1‐yl)‐1,1,3,3‐tetramethyluronium tetrafluoroborate (496 mg, 1.1 equiv., 1.55 mmol) followed by the addition of *N*,*N*‐dimethylformamide (20 mL) and DIPEA (600 mg, 0.81 mL, 3 equiv., 4.65 mmol). The mixture stirred for 15 min followed by the addition of Intermediate 1 (820 mg, 1.0 equiv., 1.41 mmol,). The reaction was stirred overnight followed by the addition of 100 mL of ethyl acetate and washed three times with brine and the organic phase concentrated under reduced pressure and purified by flash chromatography (DCM:MeOH). To the obtained product was added 20% TFA in DCM and the reaction was stirred overnight at room temperature followed by purification by reverse phase flash chromatography (H_2_O +0.1% TFA: MeOH) to yield the title compound as a TFA salt (0.93 g; 93%). ^1^H NMR (400 MHz, Methanol‐*d*_4_) *δ* 7.79 (d, *J* = 7.5 Hz, 2H), 7.62 (d, *J* = 7.5 Hz, 2H), 7.48–7.15 (m, 9H), 4.56–4.38 (m, 2H), 4.34 (d, *J* = 6.8 Hz, 2H), 4.23–4.07 (m, 2H), 3.93 (dd, *J* = 11.3, 4.8 Hz, 1H), 3.81 (dd, *J* = 11.3, 4.0 Hz, 1H), 3.72 (s, 3H), 3.29–3.24 (m, 1H), 3.11 (t, *J* = 6.7 Hz, 2H), 3.02 (dd, *J* = 14.3, 8.5 Hz, 1H), 1.96–1.62 (m, 2H), 1.57–1.38 (m, 4H). MSI (ESI): 617.3 [M+H]^+^. *t*_R_ = 4.50 min.

Intermediate 3


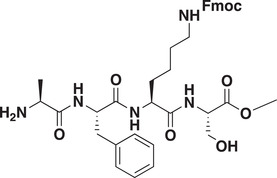


Methyl *N* ^6^‐(((9*H*‐fluoren‐9‐yl)methoxy)carbonyl)‐*N* ^2^‐l‐alanyl‐l‐phenylalanyl‐l‐lysyl‐l‐serinate: To a round bottom flask was added (*tert*‐butoxycarbonyl)‐l‐alanine (466 mg, 1.0 equiv., 2.46 mmol) and 2‐(1*H*‐benzo[d][1,2,3]triazol‐1‐yl)‐1,1,3,3‐tetramethyluronium tetrafluoroborate (1.1 g, 1.4 equiv., 3.45 mmol) followed by the addition of *N*,*N*‐dimethylformamide (25 mL) and DIPEA (892 mg, 1.2 mL, 3 equiv., 3.45 mmol). The mixture stirred for 15 min followed by the addition of Intermediate 2 (1.80 g, 1.0 equiv., 2.46 mmol,). The reaction was stirred overnight followed by the addition of 100 mL of ethyl acetate and washed three times with brine and the organic phase concentrated under reduced pressure and purified by flash chromatography (DCM:MeOH). To the obtained product was added 20% TFA in DCM and the reaction was stirred overnight at room temperature followed by purification by reverse phase flash chromatography (H_2_O +0.1% TFA: MeOH) to yield the title compound as a TFA salt (1.5 g; 89%) ^1^H NMR (400 MHz, Methanol‐*d*_4_) *δ* 7.79 (d, *J* = 7.5 Hz, 2H), 7.63 (d, *J* = 7.5 Hz, 2H), 7.38 (t, *J* = 7.4 Hz, 2H), 7.32–7.13 (m, 7H), 4.75–4.61 (m, 1H), 4.57–4.44 (m, 1H), 4.42–4.27 (m, 3H), 4.21–4.10 (m, 1H), 3.91 (dd, *J* = 11.3, 4.8 Hz, 1H), 3.79 (dd, *J* = 11.3, 4.0 Hz, 2H), 3.72 (s, 3H), 3.27–3.04 (m, 3H), 3.02–2.62 (m, 1H), 1.91–1.61 (m, 2H), 1.54–1.23 (m, 7H). MSI (ESI): 688.4 [M+H]^+^. *t*_R_ = 5.11 min.

Intermediate 4


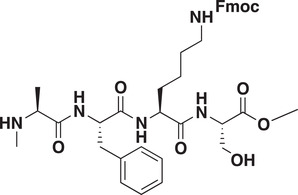


Methyl *N* ^6^‐(((9*H*‐fluoren‐9‐yl)methoxy)carbonyl)‐*N* ^2^‐methyl‐l‐alanyl‐l‐phenylalanyl‐l‐lysyl‐l‐serinate: To a round bottom flask was added (*tert*‐butoxycarbonyl)‐*N*‐methyl‐l‐alanine (145 mg, 0.9 equiv., 0.7 mmol) and 2‐(1*H*‐benzo[d][1,2,3]triazol‐1‐yl)‐1,1,3,3‐tetramethyluronium tetrafluoroborate (280 mg, 1.1 equiv., 0.9 mmol) followed by the addition of *N*,*N*‐dimethylformamide (5 mL) and DIPEA (340 mg, 0.46 mL, 3 equiv., 2.1 mmol). The mixture stirred for 15 min followed by the addition of Intermediate 2 (580 mg, 1.0 equiv., 0.8 mmol). The reaction was stirred overnight followed by the addition of 100 mL of ethyl acetate and washed three times with brine and the organic phase concentrated under reduced pressure and purified by flash chromatography (DCM:MeOH). To the obtained product was added 20% TFA in DCM and the reaction was stirred overnight at room temperature followed by purification by reverse phase flash chromatography (H_2_O +0.1% TFA: MeOH) to yield the title compound as a TFA salt (350 mg; 63%). ^1^H NMR (400 MHz, Methanol‐*d*_4_) *δ* 7.78 (d, *J* = 7.5 Hz, 2H), 7.62 (d, *J* = 7.4 Hz, 2H), 7.42–7.15 (m, 9H), 4.83–4.76 (m, 1H), 4.51 (t, *J* = 4.3 Hz, 1H), 4.44–4.38 (m, 1H), 4.34 (d, *J* = 6.9 Hz, 2H), 4.18 (t, *J* = 6.9 Hz, 1H), 3.91 (dd, *J* = 11.3, 4.7 Hz, 1H), 3.80 (dd, *J* = 11.3, 4.0 Hz, 1H), 3.72 (s, 3H), 3.68 (t, *J* = 7.0 Hz, 1H), 3.24 (dd, *J* = 14.1, 4.7 Hz, 1H), 3.17–3.07 (m, 2H), 2.91–2.83 (m, 1H), 2.30 (s, 3H), 1.86 (dq, *J* = 13.8, 7.2 Hz, 1H), 1.71 (h, *J* = 8.2 Hz, 1H), 1.56–1.48 (m, 2H), 1.47–1.37 (m, 5H). MSI (ESI): 702 [M+H]^+^. *t*_R_ = 4.57 min.

Intermediate 5


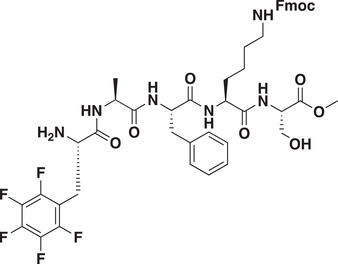


Methyl *N* ^6^‐(((9*H*‐fluoren‐9‐yl)methoxy)carbonyl)‐*N* ^2^‐((*S*)‐2‐amino‐3‐(perfluorophenyl)propanoyl)‐l‐alanyl‐l‐phenylalanyl‐l‐lysyl‐l‐serinate: To a round bottom flask was added (*tert*‐butoxycarbonyl)‐pentafluoro‐phenylalanine (19 mg, 0.9 equiv., 0.05 mmol) and 2‐(1*H*‐benzo[d][1,2,3]triazol‐1‐yl)‐1,1,3,3‐tetramethyluronium tetrafluoroborate (24 mg, 1.1 equiv., 0.08 mmol) followed by the addition of *N*, *N*‐dimethylformamide (2 mL) and DIPEA (20 mg, 0.03 mL, 3 equiv., 2 mmol). The mixture stirred for 15 min followed by the addition of Intermediate 3 (47 mg, 1.0 equiv., 0.06 mmol,). The reaction was stirred overnight followed by the addition of 100 mL of ethyl acetate and washed three times with brine and the organic phase concentrated under reduced pressure and purified by flash chromatography (DCM:MeOH). To the obtained product was added 20% TFA in DCM and the reaction was stirred overnight at room temperature followed by purification by reverse phase flash chromatography (H_2_O +0.1% TFA: MeOH) to yield the title compound as a TFA salt (43 mg; 80%) ^1^H NMR (400 MHz, Methanol‐*d*_4_) *δ* 7.76 (d, *J* = 7.6 Hz, 2H), 7.64–7.54 (m, 2H), 7.45–7.04 (m, 9H), 4.60 (t, *J* = 7.0 Hz, 1H), 4.51–4.28 (m, 5H), 4.16 (t, *J* = 6.9 Hz, 1H), 4.00 (t, *J* = 7.2 Hz, 1H), 3.88 (dd, *J* = 11.2, 4.8 Hz, 1H), 3.76 (dd, *J* = 11.3, 4.0 Hz, 1H), 3.71 (s, 3H), 3.19–3.04 (m, 4H), 2.95 (dd, *J* = 14.1, 8.3 Hz, 2H), 1.86–1.76 (m, 1H), 1.65 (dd, *J* = 14.4, 6.9 Hz, 1H), 1.47 (q, *J* = 7.1 Hz, 2H), 1.36 (d, *J* = 9.5 Hz, 2H), 1.24 (d, 7.1 Hz, 3H). MSI (ESI): 925.2 [M+H]^+^. *t*_R_ = 5.31 min.

Intermediate 6


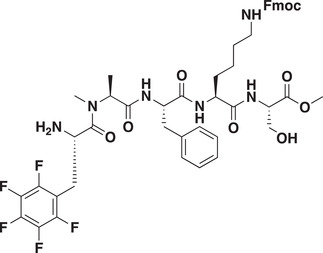


Methyl *N* ^6^‐(((9H‐fluoren‐9‐yl)methoxy)carbonyl)‐*N* ^2^‐*N*‐((*S*)‐2‐amino‐3‐(perfluorophenyl)propanoyl)‐*N*‐methyl‐l‐alanyl‐l‐phenylalanyl‐l‐lysyl‐l‐serinate: Intermediate 6 was synthesized according to the protocol for Intermediate 5 starting from Intermediate 4 (50 mg, 0.06 mmol) to yield title compound as a white powder TFA salt (35 mg, 60%). ^1^H NMR (400 MHz, Methanol‐*d*_4_) *δ* 7.74 (d, *J* = 7.5 Hz, 2H), 7.59 (d, *J* = 7.2 Hz, 2H), 7.42–7.02 (m, 9H), 4.70–4.59 (m, 1H), 4.57–4.35 (m, 3H), 4.32–4.23 (m, 2H), 4.16–4.09 (m, 1H), 3.90–3.84 (m, 1H), 3.76 (dd, *J* = 11.3, 4.1 Hz, 1H), 3.68 (s, 3H), 3.25–3.17 (m, 1H), 3.14–3.06 (m, 2H), 3.03–2.90 (m, 3H), 2.82 (s, 3H), 1.88–1.79 (m, 1H), 1.78–1.63 (m, 1H), 1.52–1.34 (m, 4H), 1.29 (d, *J* = 7.1 Hz, 3H). MSI (ESI): 939.2 [M+H]^+^. *t*_R_ = 5.32 min.

UNC6261


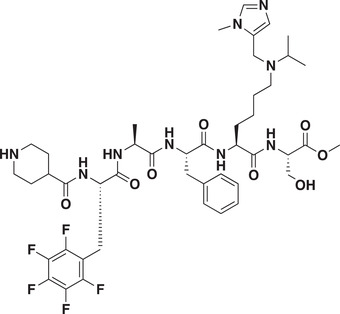


Methyl *N* ^6^‐isopropyl‐*N* ^6^‐((1‐methyl‐1*H*‐imidazol‐5‐yl)methyl)‐*N* ^2^‐((*S*)‐3‐(perfluorophenyl)‐2‐(piperidine‐4‐carboxamido)propanoyl)‐l‐alanyl‐l‐phenylalanyl‐l‐lysyl‐l‐serinate (UNC6261): To a round bottom flask was added 1‐(*tert*‐butoxycarbonyl)piperidine‐4‐carboxylic acid (466 mg, 1.0 equiv., 2.46 mmol) and 2‐(1*H*‐benzo[d][1,2,3]triazol‐1‐yl)‐1,1,3,3‐tetramethyluronium tetrafluoroborate (1.1 g, 1.4 equiv., 3.45 mmol) followed by the addition of *N*,*N*‐dimethylformamide (25 mL) and DIPEA (892 mg, 1.2 mL, 3 equiv., 3.45 mmol). The mixture stirred for 15 min followed by the addition of Intermediate 5 (1.80 g, 1.0 equiv., 2.46 mmol,). The reaction was stirred overnight and solvent removed under reduced pressure. To the crude oil was added 20% diethylamine in DMF and the reaction stirred for 4 h and the solvent was removed under reduced pressure. The crude oil was purified via reverse phase chromatography H_2_O +0.1% TFA: MeOH) to yield free lysine amine. The free amine (70 mg, 0.08 mmol) was dissolved in methanol (5 mL) followed by the addition of acetone (14 mg 18 µL 3 equiv., 0.24 mmol) and sodium caynoborohydride (21 mg, 0.3 mmol, 4 equiv.) and the reaction stirred overnight and was monitored for completion by LC‐MS upon which **1**‐methyl‐1*H*‐imidazole‐5‐carbaldehyde (15 mg 19 µL, 4 equiv., 0.32 mmol) was added along with 1 equiv. of sodium cyanoborohydride and the reaction heated to 50 °C and allowed to proceed until completion as monitored by LC‐MS. Upon completion, the reaction was concentrated under reduced pressure and the resulting oil dissolved in 20% TFA in DCM. The mixture was allowed to stir overnight followed by concentration under vacuum and purification via reverse phase flash chromatography (H_2_O +0.1% TFA: MeOH) to yield the title compound as a TFA salt (13 mg, 20% yield across four steps). ^1^H NMR (400 MHz, Methanol‐*d*_4_) *δ* 9.07–9.02 (d, *J* = 1.4 Hz, 1H), 8.00–7.94 (d, *J* = 1.4 Hz, 1H), 7.33–7.13 (m, 5H), 4.67–4.61 (dd, *J* = 9.0, 5.8 Hz, 1H), 4.59–4.51 (m, 3H), 4.50–4.46 (t, *J* = 4.5 Hz, 1H), 4.45–4.39 (ddd, *J* = 8.2, 5.7, 2.2 Hz, 1H), 4.30–4.21 (dd, *J* = 7.2, 3.8 Hz, 1H), 4.04–3.97 (s, 3H), 3.94–3.89 (dd, *J* = 11.4, 4.7 Hz, 1H), 3.88–3.83 (m, 1H), 3.82–3.77 (m, 1H), 3.76–3.70 (s, 3H), 3.43–3.32 (m, 3H), 3.26–3.14 (m, 4H), 3.08–2.95 (m, 4H), 2.65–2.53 (tt, *J* = 11.0, 3.8 Hz, 1H), 1.97–1.66 (m, 8H), 1.53–1.36 (m, 8H), 1.30–1.21 (d, *J* = 7.1 Hz, 3H). MSI (ESI): 475.8 [M+2H]^2+^/2. *t*_R_ = 2.67 min.

UNC7394


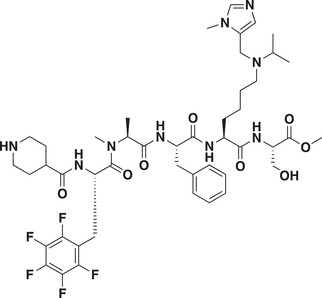


**Scheme 2:**
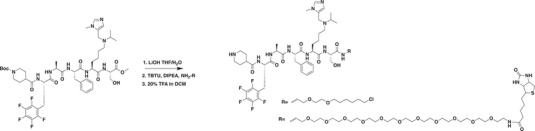
Synthesis of UNC6261‐Biotin and UNC6261‐CT.

Methyl *N* ^6^‐isopropyl‐*N* ^6^‐((1‐methyl‐1*H*‐imidazol‐5‐yl)methyl)‐*N* ^2^‐*N*‐methyl‐*N*‐((*S*)‐3‐(perfluorophenyl)‐2‐(piperidine‐4‐carboxamido)propanoyl)‐l‐alanyl‐l‐phenylalanyl‐l‐lysyl‐l‐serinate (UNC7394): UNC7394 was synthesized as using the same steps as described for UNC6261 starting from intermediate 6 (520 mg 0.74 mmol) to give title compound as a TFA salt (100 mg 16% yield across 4 steps). ^1^H NMR (400 MHz, Methanol‐*d*_4_) *δ* 9.05 (s, 1H), 7.96 (s, 1H), 7.32–7.12 (m, 5H), 5.12–5.02 (m, 1H), 4.73–4.61 (m, 1H), 4.58–4.41 (m, 5H), 4.00 (s, 3H), 3.96–3.77 (m, 3H), 3.73 (s, 3H), 3.38–3.32 (m, 2H), 3.25–3.09 (m, 3H), 3.08–2.87 (m, 5H), 2.74* (s, 2H), 2.58–2.49 (m, 1H), 2.26* (s, 1H), 2.07–1.57 (m, 8H), 1.53–1.36 (m, 8H), 1.25* (d, *J* = 7.1, 2H), 1.05* (d, *J* = 6.9, 1H). *Rotamers of *N*‐methylated alanine. MSI (ESI): 482.9 [M+2H]^2+^/2. *t*_R_ = 2.39 min (**Scheme** **2**).

UNC6261‐Biotin


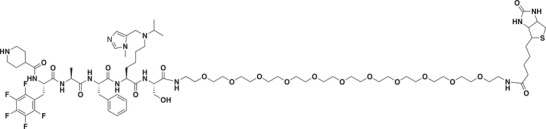


*Tert*‐butyl 4‐(((4*S*,7*S*,10*S*,13*S*,16*S*)‐10‐benzyl‐4‐(hydroxymethyl)‐7‐(4‐(isopropyl((1‐methyl‐1*H*‐imidazol‐5‐yl)methyl)amino)butyl)‐13‐methyl‐3,6,9,12,15‐pentaoxo‐17‐(perfluorophenyl)‐2‐oxa‐5,8,11,14‐tetraazaheptadecan‐16‐yl)carbamoyl)piperidine‐1‐carboxylate (30 mg, 0.03 mmol) was dissolved in THF (1 mL) and 200 µL of saturated LiOH were added and the reaction stirred overnight. The mixture was concentrated and purified via reverse phase flash chromatography (Water: 0.1% TFA: ACN) to give a free carboxylic acid intermediate (19 mg, 66%). The intermediate was dissolved in DMF (1 mL) and 2‐(1*H*‐benzo[d][1,2,3]triazol‐1‐yl)‐1,1,3,3‐tetramethyluronium tetrafluoroborate (5.8 mg, 0.018 mmol, 1.1 equiv.) was added followed by DIPEA (6.4 mg, 8.6 µL, 0.05 mmol, 3 equiv.) and the mixture stirred for 15 min after which *N*‐(35‐amino‐3,6,9,12,15,18,21,24,27,30,33‐undecaoxapentatriacontyl)‐5‐(2‐oxohexahydro‐1*H*‐thieno[3,4‐d]imidazol‐4‐yl)pentanamide (15 mg, 0.02 mmol, 1.2 equiv.) was added and the reaction stirred overnight. The crude mixture was concentrated under vacuum and dissolved in 20% TFA in DCM and stirred for 1 h. This mixture was concentrated and purified via reverse phase flash chromatography (H_2_O +0.1% TFA: MeOH) to yield the title compound as a TFA salt (6 mg, 21% over three steps). ^1^H NMR (400 MHz, Methanol‐*d*_4_) *δ* 9.11–9.01 (s, 1H), 8.04–7.92 (s, 1H), 7.38–7.09 (m, 5H), 4.70–4.60 (m, 1H), 4.60–4.45 (m, 4H), 4.42–4.20 (m, 4H), 4.05–3.92 (m, 3H), 3.91–3.73 (m, 3H), 3.67–3.57 (m, 40H), 3.57–3.49 (m, 4H), 3.47–3.33 (m, 6H), 3.25–3.15 (m, 5H), 3.08–2.85 (m, 5H), 2.75–2.65 (d, *J* = 12.7 Hz, 1H), 2.62–2.51 (m, 1H), 2.25–2.17 (t, *J* = 7.4 Hz, 2H), 1.99–1.50 (m, 12H), 1.51–1.39 (m, 10H), 1.32–1.21 (d, *J* = 7.2 Hz, 3H). MSI (ESI): 845.2 [M+2H]^2+^. *t*_R_ = 2.60 min.

UNC6261‐CT


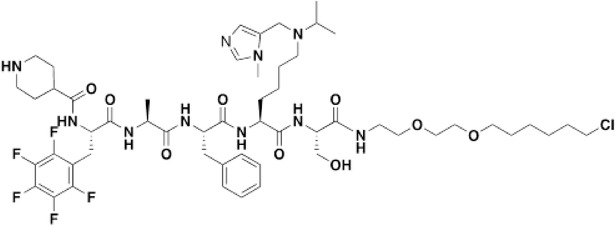


UNC6261‐CT was prepared as described above from *tert*‐butyl 4‐(((4*S*,7*S*,10*S*,13*S*,16*S*)‐10‐benzyl‐4‐(hydroxymethyl)‐7‐(4‐(isopropyl((1‐methyl‐1*H*‐imidazol‐5‐yl)methyl)amino)butyl)‐13‐methyl‐3,6,9,12,15‐pentaoxo‐17‐(perfluorophenyl)‐2‐oxa‐5,8,11,14‐tetraazaheptadecan‐16‐yl)carbamoyl)piperidine‐1‐carboxylate (28 mg, 0.03 mmol) and 2‐(2‐((6‐chlorohexyl)oxy)ethoxy)ethan‐1‐amine (9.1 mg, 0.04 mmol 1.3 equiv.) to give the title compound as a TFA salt (4 mg, 15% over three steps). ^1^H NMR (400 MHz, Methanol‐*d*_4_) *δ* 8.83–8.76 (s, 1H), 7.80–7.72 (s, 1H), 7.33–7.17 (m, 5H), 4.75–4.61 (d, *J* = 7.8 Hz, 2H), 4.61–4.21 (m, 8H), 3.99–3.90 (m, 3H), 3.82–3.67 (m, 3H), 3.63–3.51 (m, 5H), 3.50–3.35 (m, 5H), 3.24–3.13 (m, 3H), 3.10–2.91 (m, 5H), 2.61–2.48 (m, 2H), 2.01–1.53 (m, 12H), 1.51–1.28 (s, 12H), 1.30–1.23 (d, *J* = 7.2 Hz, 3H). MSI (ESI): 571 [M+2H]^2+^. *t*_R_ = 2.81 min.

### Statistics Analysis

All the data were represented as the mean ± SEM. The analysis of data was performed with GraphPad Prism (version 8.00, La Jolla, California, USA). Normality tests were formally underwent using D'Agostino–Pearson omnibus normality test and nonparametric tests were used for the data which failed the tests. Two groups were compared by a two‐tailed Student's *t* test. Comparisons among groups were performed by one‐way ANOVA followed by Tukey's multiple comparisons test. Comparisons among groups with multiple time points, voltage points or current points were performed by two‐way ANOVA followed by Bonferroni's post hoc test. A *p* value less than 0.05 was considered statistically significant. (*p < 0.05, **p < 0.01, ***p < 0.001, and n.s. no statistical significance).

### Data Availability

The ChIP‐seq raw and processed data files has been deposited in the GEO datasets and the accession number is GSE190751.

## Conflict of Interest

The authors declare no conflict of interest.

## Author Contributions

Z.‐W.S. performed and analyzed PCR, western blotting, immunochemistry, ChIP, and electrophysiological experiments. Z.‐W.S. and L.C. performed the behavioral tests. Z.‐W.S. and Y.C. performed the genotyping. J.M.W. synthesized the compounds and performed biochemical and target engagement assays. C.A.F. performed the CAPA assay. J.L.N.‐D. expressed and purified relevant proteins. S.B., A.D., and J.M. performed structural studies; protein purification, crystallization, structure solving/data refinement, and deposition of the X‐ray structure. Z.‐W.S., J.M.W., L.I.J., and Y.W. wrote the paper.

## Supporting information

Supporting Information

## Data Availability

The data that support the findings of this study are available on request from the corresponding author. The data are not publicly available due to privacy or ethical restrictions.
