# *Cdyl* Deficiency Brakes Neuronal Excitability and Nociception through Promoting *Kcnb1* Transcription in Peripheral Sensory Neurons

**DOI:** 10.1002/advs.202304061

**Published:** 2023-09-05

**Authors:** Zhao‐Wei Sun, Jarod M. Waybright, Serap Beldar, Lu Chen, Caroline A. Foley, Jacqueline L. Norris‐Drouin, Tian‐Jie Lyu, Aiping Dong, Jinrong Min, Yu‐Pu Wang, Lindsey I. James, Yun Wang

*Adv. Sci*. **2022**, *9*, 2104317

DOI: 10.1002/advs.202104317

In the original published article the structure of the chemical compound reported in Figure 7a is incorrect. Please find the correct Figure 7 below.

**Figure 7:**
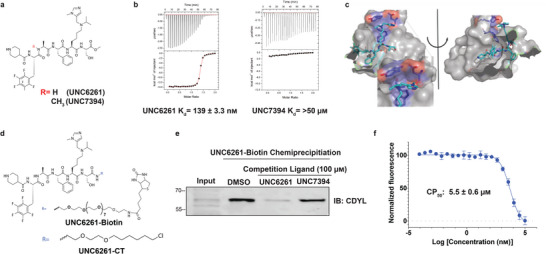
UNC6261 is a potent antagonist of the CDYL chromodomain. a) Structure of UNC6261 and negative control ligand UNC7394. b) UNC6261 potently binds the CDYL chromodomain as determined by isothermal titration calorimetry whereas UNC7394 demonstrates no measurable binding. Data are the mean ± S.D. c) X‐ray crystal structure of UNC6261 bound to CDYL highlighting the surface groove and aromatic cage (bottom left) interactions (PDB: 7N27). d) Structure of UNC6261‐Biotin and UNC6261‐CT. e) Chemiprecipitation of full‐length CDYL from MDA‐MB‐231 cell lysates via UNC6261‐Biotin in the presence of UNC6261 and UNC7394. n = 2 biological replicates. f) CAPA analysis of UNC6261‐CT. Data are the mean ± SEM.

