# Supplementary material for: Cdyl Deficiency Brakes Neuronal Excitability and Nociception through Promoting Kcnb1 Transcription in Peripheral Sensory Neurons
Source: Adv Sci (Weinh). 2022 Feb 4;9(10):2104317. doi: 10.1002/advs.202104317 (PMC8981457; doi:10.1002/advs.202104317)

ADVANCED SCIENCE
Open Access

Supporting Information

for *Adv. Sci.*, DOI 10.1002/advs.202104317

Cdyl Deficiency Brakes Neuronal Excitability and Nociception through Promoting *Kcnb1* Transcription in Peripheral Sensory Neurons

Zhao-Wei Sun, Jarod M. Waybright, Serap Beldar, Lu Chen, Caroline A. Foley, Jacqueline L. Norris-Drouin, Tian-Jie Lyu, Aiping Dong, Jinrong Min, Yu-Pu Wang, Lindsey I. James and Yun Wang**

ADVANCED SCIENCE
Open Access

Supporting Information

for *Adv. Sci.*, DOI: 10.1002/advs.202104317

Cdyl Deficiency Brakes Neuronal Excitability and Nociception through Promoting *Kcnb1* Transcription in Peripheral Sensory Neurons

Zhao-Wei Sun, Jarod M.Waybright, Serap Beldar, Lu Chen, Caroline A. Foley, Jacqueline L. Norris-Drouin, Tian-Jie Lyu, Aiping Dong, Jinrong Min, Yu-Pu Wang, Lindsey I. James and Yun Wang**

Supporting Information

Cdyl Deficiency Brakes Neuronal Excitability and Nociception through Promoting *Kcnb1* Transcription in Peripheral Sensory Neurons

Zhao-Wei Sun, Jarod M.Waybright, Serap Beldar, Lu Chen, Caroline A. Foley, Jacqueline L. Norris-Drouin, Tian-Jie Lyu, Aiping Dong, Jinrong Min, Yu-Pu Wang, Lindsey I. James* and Yun Wang*

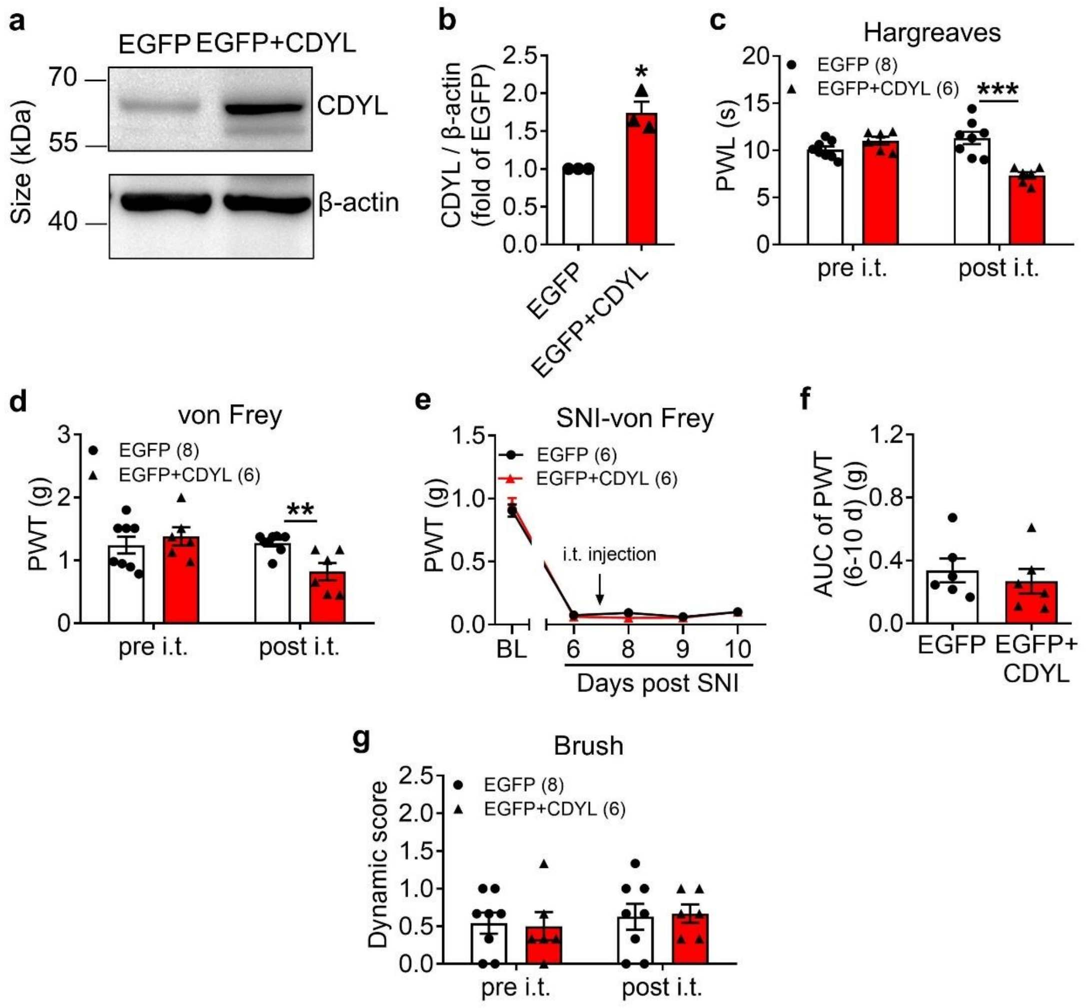

Figure S1. Intrathecal delivery of exogenous CDYL plasmids increases pain sensitivity. a) Representative image of CDYL expression in DRG after transfection with the indicated plasmids. B) Quantification of CDYL protein levels in (a). n = 3 biological replicates. Student's paired t test, $^{*}p < 0.05$. c, d) Basal PWL (c) and PWT (d) before and after injection with EGFP or EGFP+CDYL plasmid. Two-way ANOVA with Sidak's post hoc test, $^{**}p < 0.01$, $^{***}p < 0.001$. e) Time course of PWT of the ipsilateral hind paws after SNI in EGFP or EGFP+CDYL plasmid-injected mice. Two-way ANOVA with Sidak's post hoc test, no statistical significance. f) AUC from 6 to 10 d after SNI was compared. Student's unpaired t test, no statistical

significance. g) Tactile sensitivity before and after injection with EGFP or EGFP+CDYL plasmid. Two-way ANOVA with Sidak's post hoc test, no statistical significance. Data are the mean ± SEM.

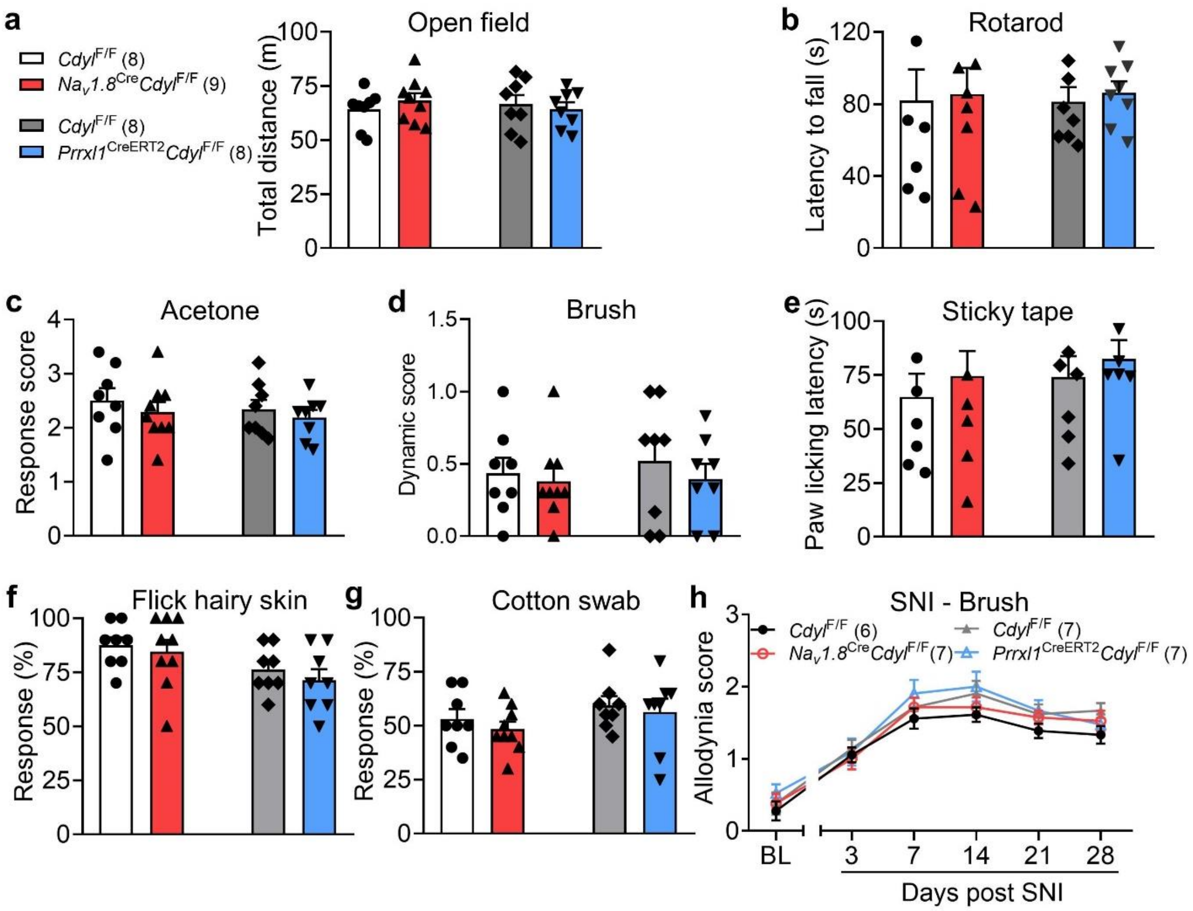

Figure S2. *Cdyl* deficiency in male mouse DRGs does not alter motor function or cold and touch sensations. a, b) Locomotor activities and motor coordination skills of *Cdyl* cKO mice and their littermate controls were assessed by open field test (a) and rotarod test (b). Student's unpaired *t* test, no statistical significance. c) Response to acetone-evoked cooling was tested by acetone test. Student's unpaired *t* test, no statistical significance. d-g) Touch sensation was measured by dynamic brush stimuli (d), sticky tape stimuli (e), flicky hairy skin stimuli (f) and cotton swab stimuli (g). Student's unpaired *t* test, no statistical significance. h) Time course of dynamic allodynia of the ipsilateral hind paw after SNI. Two-way ANOVA with Sidak's post hoc test, no statistical significance.

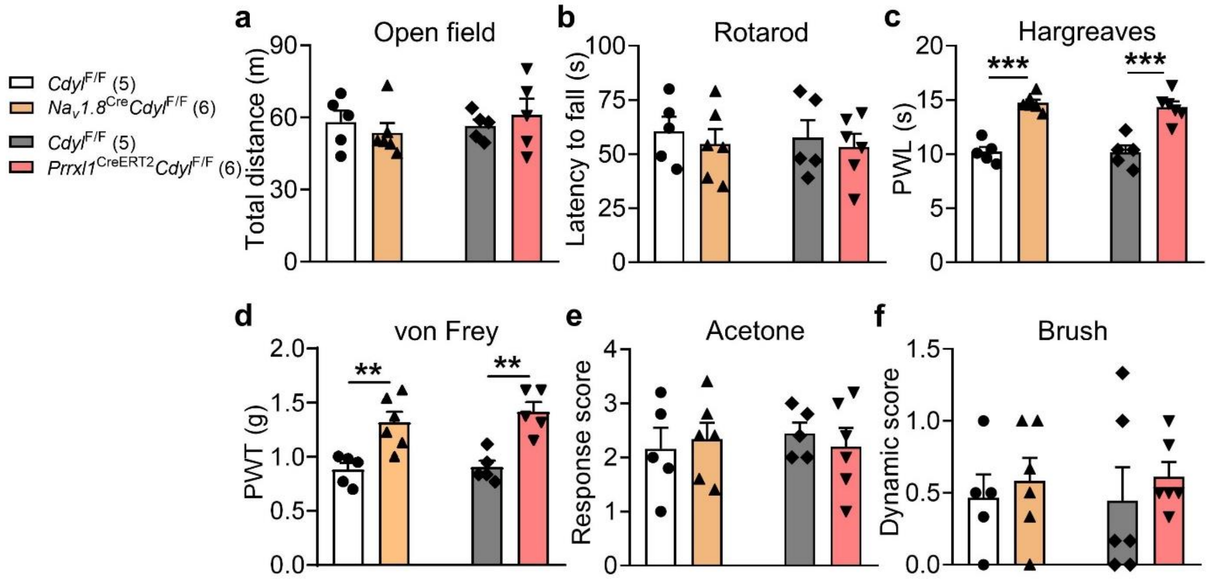

Figure S3. *Cdyl* deficiency in female mouse DRGs decreases pain sensitivity. a, b) Locomotor activities and coordination skills of female mice were tested by open field test (a) and rotarod test (b). Student's unpaired *t* test, no statistical significance. c, d) Basal PWT and PWL were tested by Hargreaves's method (c) and von Frey assay (d), respectively. Student's unpaired *t* test, $^{**}p < 0.01$, $^{***}p < 0.001$. e, f) Cold and touch sensation were tested by the acetone drop test (e) and brush test (f), respectively. Student's unpaired *t* test, no statistical significance. Data are the mean ± SEM.

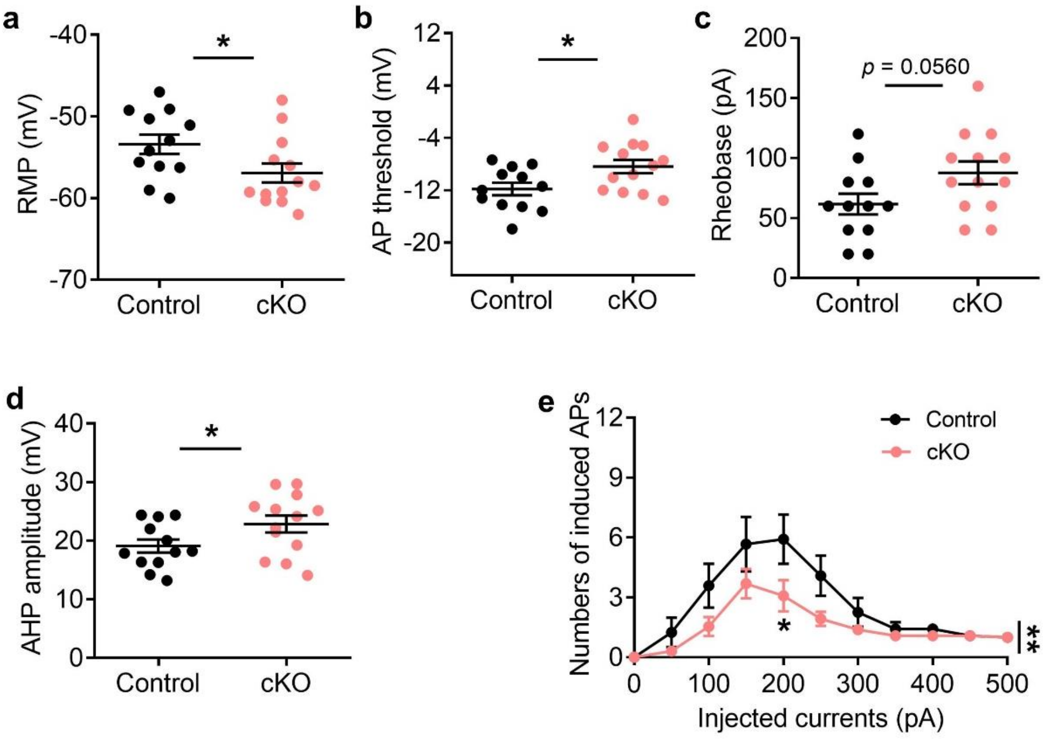

Figure S4. *Cdyl* deficiency in female mouse DRG decreases neuronal excitability. a-d) The RMP (a), AP threshold (b), current threshold (c) and AHP amplitude (d) obtained from DRG neurons of female *Cdyl* cKO mice and their littermate controls. Student's unpaired *t* test, $^{*}p < 0.05$. e) Numbers of APs induced by indicated currents. Two-way ANOVA, group effect: $^{**}p < 0.01$, shown at the end of the lines; post-test: Sidak's post-hoc test, $^{*}p < 0.05$, shown below the lines. $n_{control} = 12$; $n_{cKO} = 13$. Data are presented as mean ± SEM.

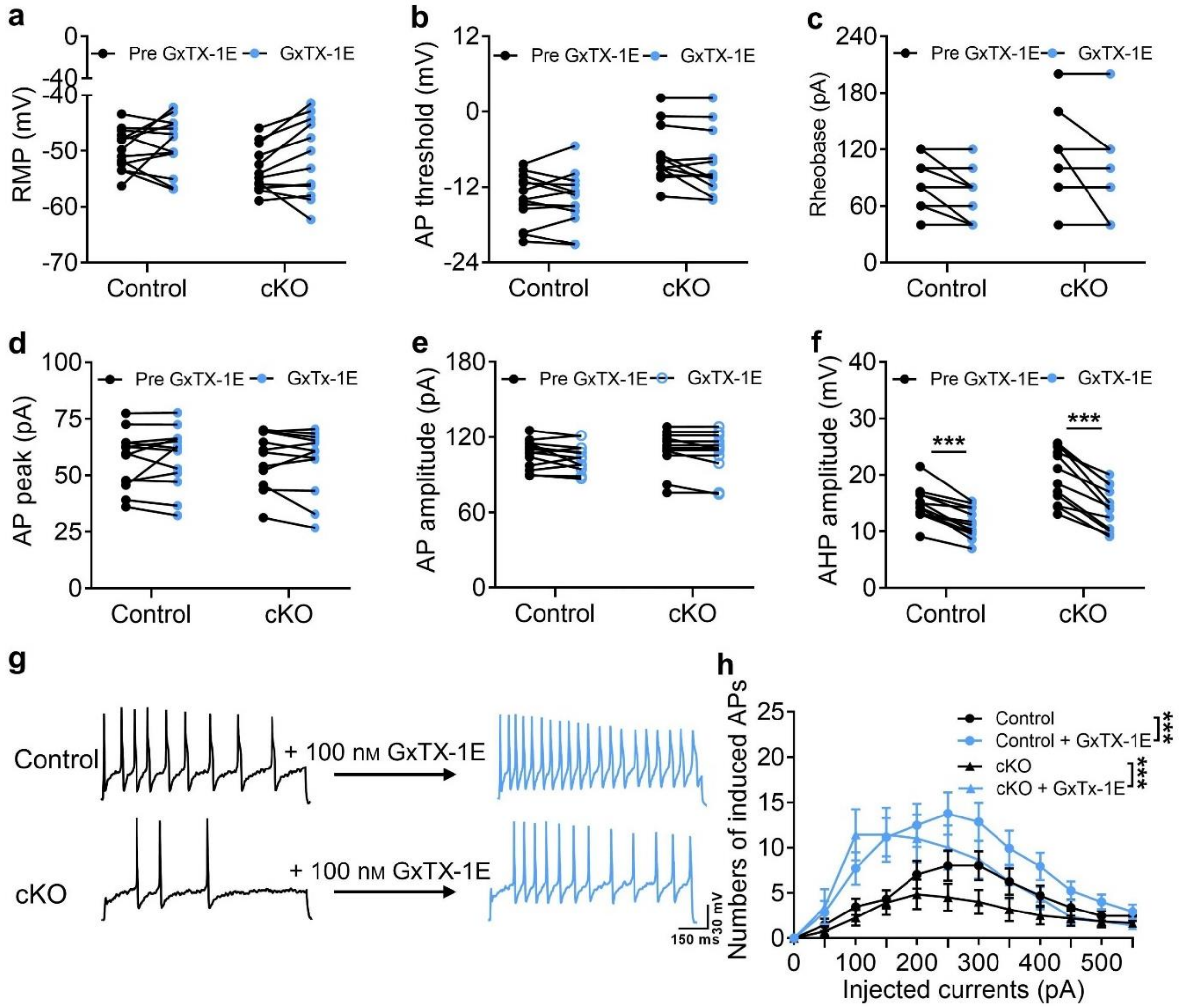

Figure S5. CDYL modulates neuronal excitability through Kv2.1 channel in the DRG. a-e) The RMP (a), AP threshold (b), rheobase (c), AP peak (d) and AP amplitude (e) obtained from DRG neurons of *Cdyl* cKO mice and their controls in the presence or absence of 100 nM GxTX-1E. Student's paired *t* test, no statistical significance. f) AHP amplitude in DRG neurons of *Cdyl* cKO and control mice in the presence or absence of 100 nM GxTX-1E. Two-way ANOVA with Sidak's post hoc test, $^{***}p < 0.001$. g, h) Representative traces (g) and numbers (h) of evoked APs in DRG neurons in the presence or absence of 100 nM GxTX-1E. Scale bars, 150 ms and 30 mV.

Two-way ANOVA, group effect: $^{***}p < 0.001$. $n_{control} = 15$, $n_{cKO} = 14$. Data are the mean ± SEM.

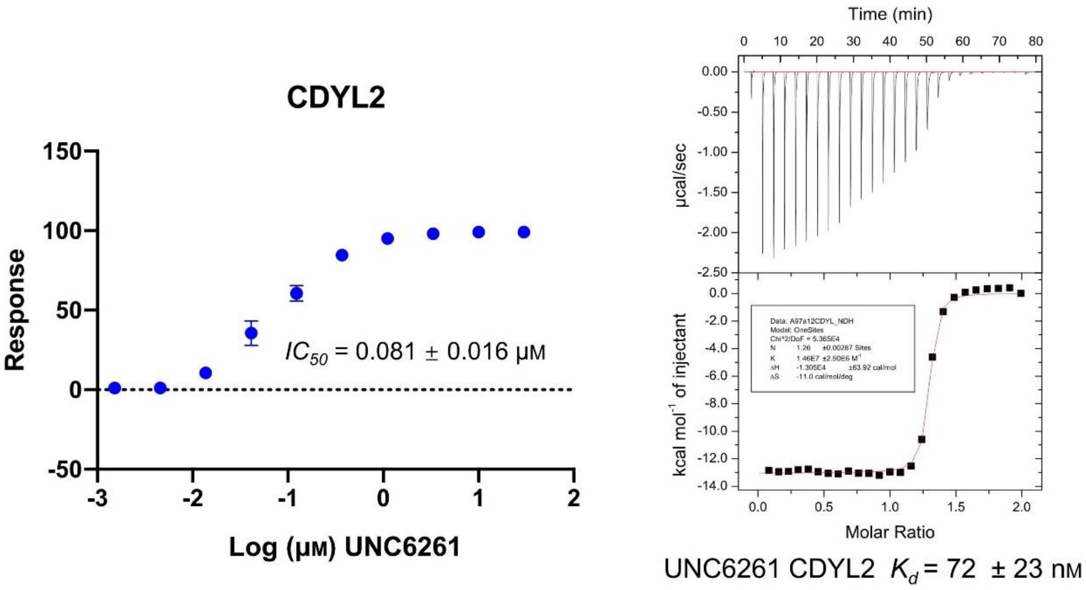

Figure S6. UNC6261 potently binds the chromodomain of CDYL2 as determined by TR-FRET (left) and ITC (right).

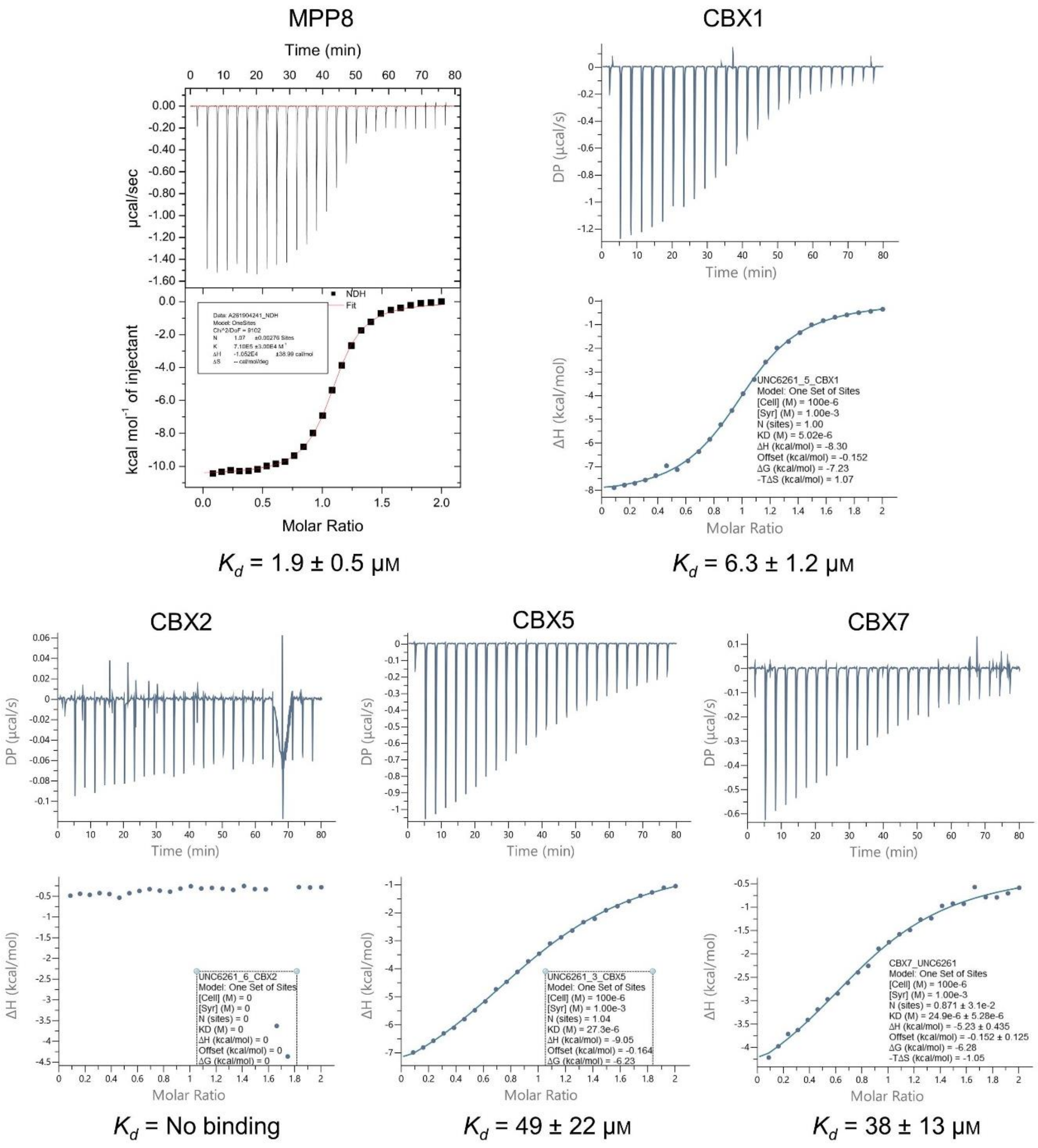

Figure S7. ITC Analysis of UNC6261 binding to a panel of chromodomains.

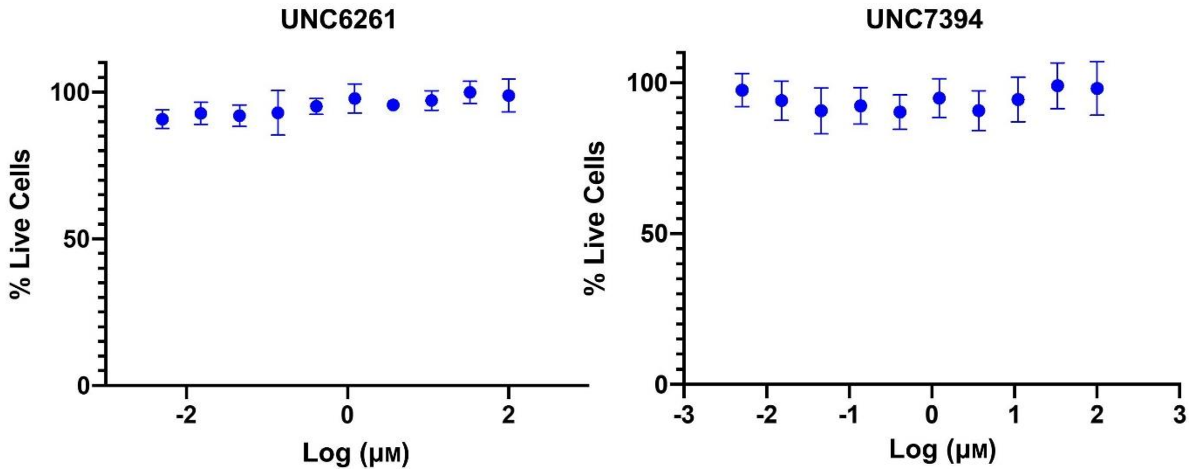

Figure S8. UNC6261 and UNC7394 demonstrate no measurable toxicity in U2OS cells using a CellTiter-Glo™ viability assay.

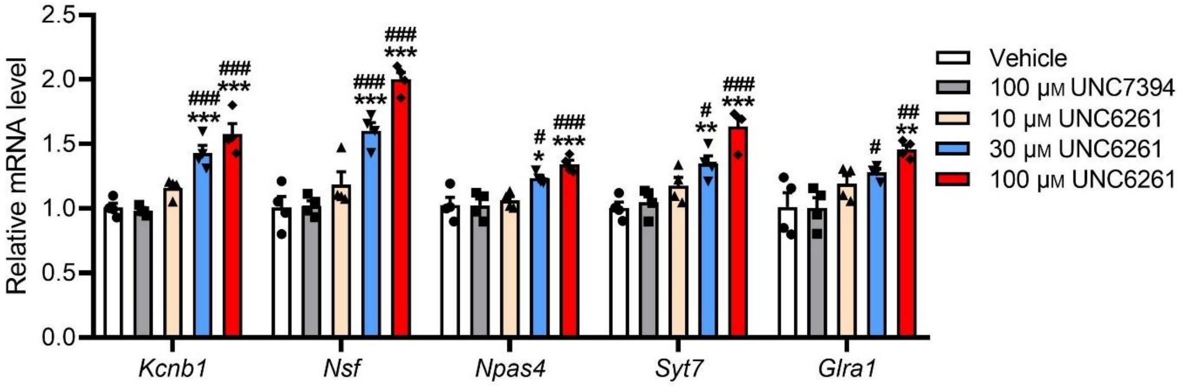

Figure S9. CDYL antagonist UNC6261 promotes the transcription of CDYL target genes. The mRNA levels of representative CDYL downstresm genes in DRG neurons treated with vehicle, 100 μM UNC7394 or 10, 30, 100 μM UNC6261 for 24 h were assessed by qRT-PCR. One-way ANOVA with Turkey's post-hoc test, $^{*}p < 0.05$, $^{**}p < 0.01$, $^{***}p < 0.001$ versus vehicle; $^{\#}p < 0.05$, $^{\#\#}p < 0.01$, $^{\#\#\#}p < 0.001$ versus UNC7394.

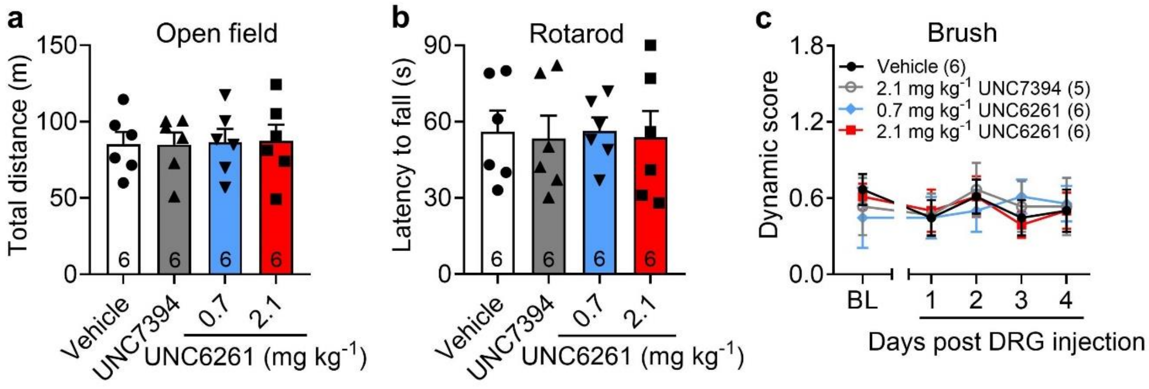

Figure S10. Focal DRG application of CDYL antagonist does not influence motor function or tactile sensation. a-b) Motor activity and function of mice 24 h after injection of UNC6261, UNC7394 or vehicle were assessed by the open field test (a) and rotarod test (b), respectively. Student's unpaired *t* test, no statistical significance. c) Time course of touch sensitivity after DRG injection was assessed by brush test. Two-way ANOVA with Sidak's post hoc test, no statistical significance. Data are the mean ± SEM.

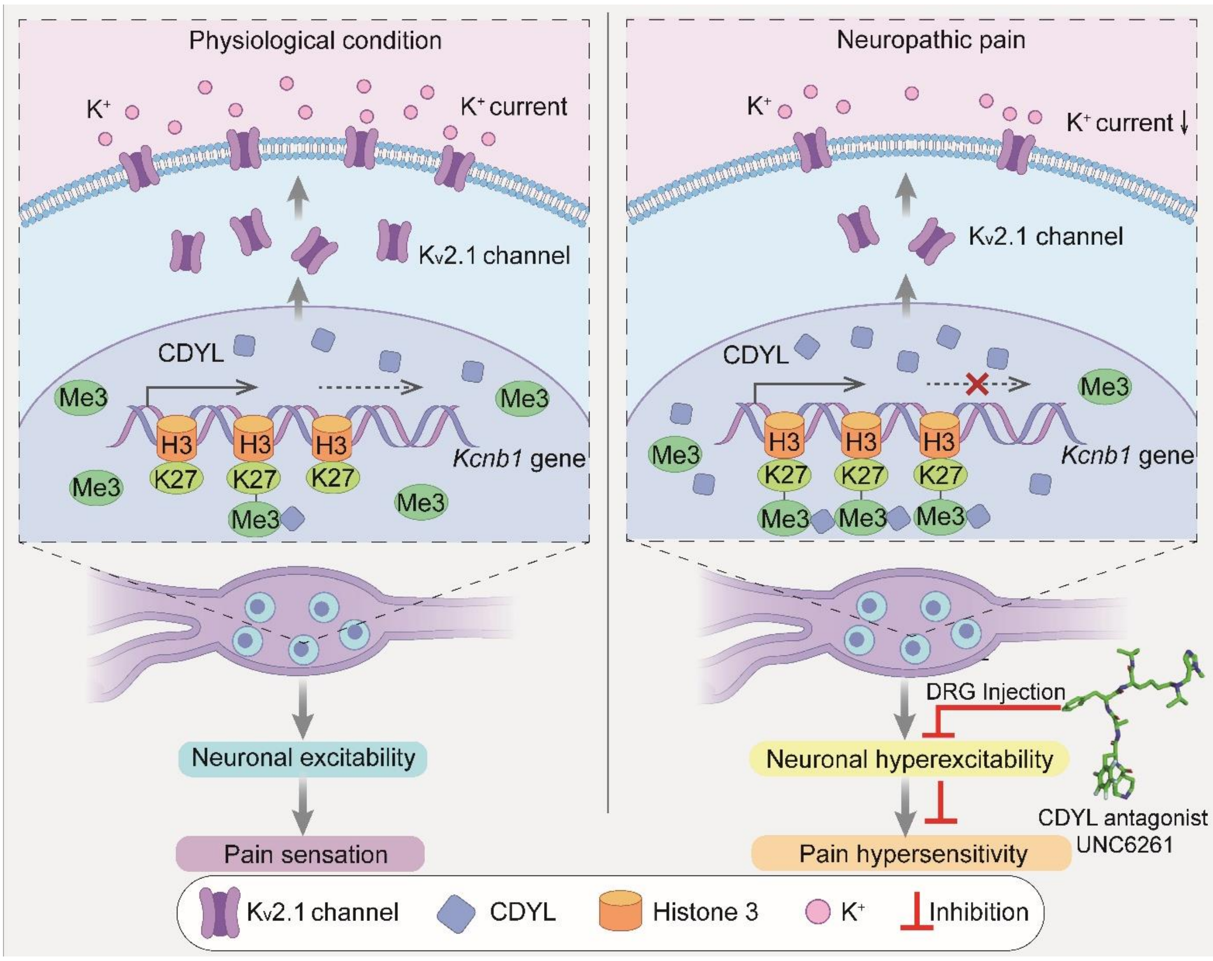

Figure S11. Schematic diagram of the regulatory effect of peripheral CDYL and its antagonist on neuronal excitability and pain sensation. Under physiological conditions, CDYL maintains neuronal excitability and pain sensation by recruiting H3K27me3 activity at the Kcnb1 intron element. Under a neuropathic pain state, upregulated CDYL expression promotes H3K27me3 modification to repress *Kcnb1* transcription. Additionally, the novel CDYL antagonist UNC6261 successfully inhibited DRG neuronal excitability and pain processing.

Table S1. qRT - PCR primers

Gene	Strand	Primer
Npas4	Forward	GTCCTAATCTACCTGGGCTTTG
	Reverse	GTCACTGATAGGGTAGTTATTGGC
Glra1	Forward	TTGCCTGCTCTTCGTGTTCT
	Reverse	GCATGGGGCTCTTGTGATGT
Snap25	Forward	TCATCTGGTGGCTCTAATTCCTAA
	Reverse	AACAGCACATTGAGCATTCCTAA
Nsf	Forward	CATTTGCTTCTCGGGTGTTC
	Reverse	GCCTCTGATTCTCCCACATAC
Crhr2	Forward	GGCAAGGAAGCTGGTGATTTG
	Reverse	GGCGTGGTGGTCCTGCCAGCG
Syt7	Forward	CTACAACCCCTCTGCCAAC
	Reverse	ATCAGCCACACCTTCACATAG
Cacng3	Forward	CCCTCATCCCCCAGACCTTA
	Reverse	CCGATGCTATGACTGCACCT
Slc12a5	Forward	AAGGGCAGAGAGTACGATGG
	Reverse	CCTGGGGTAGGTTGGTGTAG
Kcnb1	Forward	GGAGTACCAGCCCCAAGTTC
	Reverse	CGGGACCTTTCCCAGGTAAC

Pex5l	Forward	TCGGCCAGTGAGAAGGGATA
	Reverse	GAACAGGATGGTGACAGGCA
Cacng2	Forward	TTCGCTGCCTTCAGCTTGAT
	Reverse	TTGAAGTTCCCTTCGAGGCAG
Cdyl	Forward	GACGCTATCAGAAACTTCGTG
	Reverse	ACAGCATTTCATTCGCAGA
Gapdh	Forward	GGTGCTGAGTATGTCGTGGA
	Reverse	CCTTCCACAATGCCAAAGTT

Table S2. ChIP - qPCR primers

Gene	Strand	Primer
Npas4	Forward	AATCCAGGTAGTGCTGCCAC
	Reverse	TCTCACCCTACAGGCACTCC
Glra1	Forward	AGTAGAGGGACGAGGTGTCA
	Reverse	TCCAGCGAGTAGAGCTTCAG
Snap25	Forward	ATGTGCTGCAAAGTGGGAGA
	Reverse	CGGTGCTGAAAGGTCAGGG
Nsf	Forward	AGCTACAATGTCCGAGACTGC
	Reverse	TTGTGTGGAACCTTCAGCCA
Crhr2	Forward	AAAGCCTATCGCCATCTCCG
	Reverse	ACTGCGAGAGGAACTGAACTG
Syt7	Forward	ATCAGAGGGGCGGAGTAGAG
	Reverse	GAAACAGATGTGGCCCGGAT
Cacng3	Forward	CTAGGACCACGAGGAACGGT
	Reverse	CCAGCCTCCCACTTCTCAAG
Slc12a5	Forward	CAGACACGGGAGGCTTACTG
	Reverse	TGGGCTTTTCCCCTTCTGTG
Kcnb1	Forward	GTCCCGTCGAGTGCTGAAA
	Reverse	CCTCTCCCTTGCCTCCCAAT

Kcnb1 primer a	Forward	CACGCACATAGGGACGCAAG
	Reverse	ATGAGGGACAGCCATACGCC
Kcnb1 primer b	Forward	TTCTCCCTGCCTCTCCCTTG
	Reverse	GATGGCTGCTGTTCCTCCC
Pex5l	Forward	AGGATGGATTGTGAAGTGACTAGG
	Reverse	CGCTCAGTGCTGGTTTCTGA
Cacng2	Forward	CTCGTCCACTTACTGCATCG
	Reverse	GTGGGGGAGTTGTCCAGTTC
Cacna1a	Forward	TGCCTAACCATTCACAGGACT
	Reverse	AACCCCACAGCCAGGATTAG
Kcnc3	Forward	GCCTGGAAGGAGTTAAGGCA
	Reverse	CCAGAACCATGGACAGCTCC
Akap7	Forward	CTGTCCCTTGAGAACCCGTG
	Reverse	TTGCTCAGCCCGCCTATTTT
Gapdh	Forward	GTGCCTCATCCTTGCACAACTG
	Reverse	CCCCACAAGGCAAACAGAAATAAG

Table S3. Data collection and refinement statistics for CDYL1 with UNC6261

	CDYL1/UNC6261
**PDB code**	7N27
**Data collection**	
Space group	$P2_12_12_1$
Cell dimensions	
a, b, c (Å)	62.97, 76.39 80.63
α, β, γ (°)	90.00, 90.00, 90.00
Resolution(Å)	50.00-1.84 (1.87-1.84)*
R_{svm}	0.083 (0.752)*
$I/\sigma I$	30.1 (1.84)*
Completeness (%)	99.3 (98.8)*
Redundancy	5.9 (4.2)*
**Refinement**	
Resolution(Å)	48.64-1.85
No. of reflections	31770 (1542)
R_{work}/R_{free}	0.226/0.263
No. atoms/average B-factor[Å2]	
Protein	2832/35.3
Compound	108/45.3
Water	69/32.9

R.m.s deviations	
Bond lengths (Å)	0.013
Bond angles (°)	1.511
Ramachandran plot	
Most favoured (%)	96.8
Additional allowed (%)	3.2
Outliers (%)	0

*The values in parentheses refer to statistics in the highest resolution bin.

^{1}H NMR and LC-MS Spectra of Chemical Compounds

^{1}H NMR and LC-MS Spectra at 220 and 254 nm of Intermediate 1

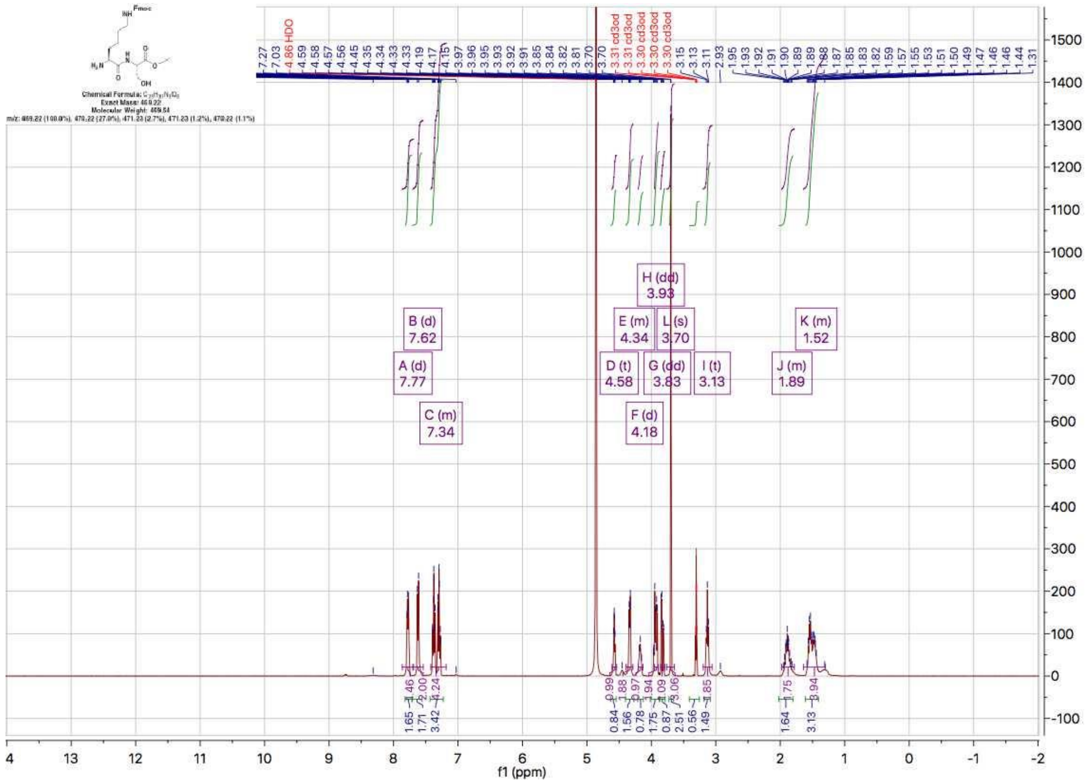

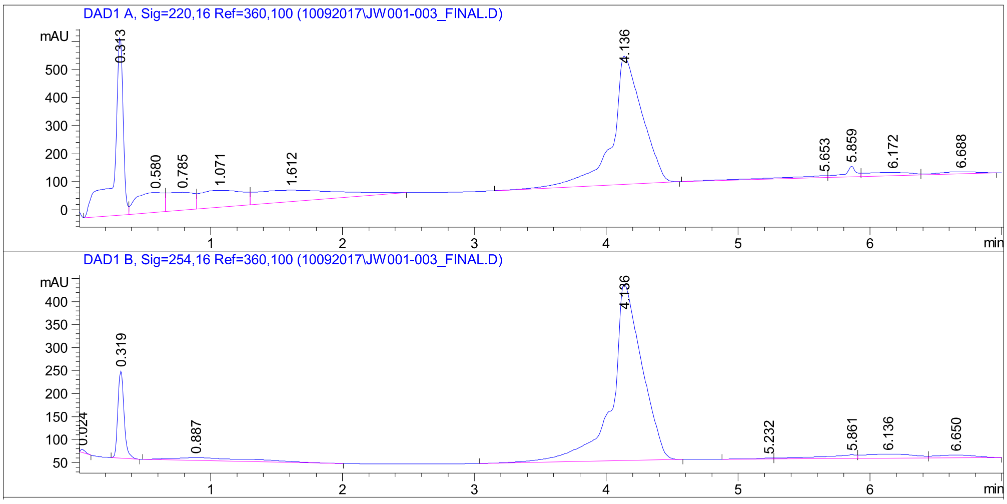

^{1}H NMR and LC-MS Spectra at 220 and 254 nm of Intermediate 2

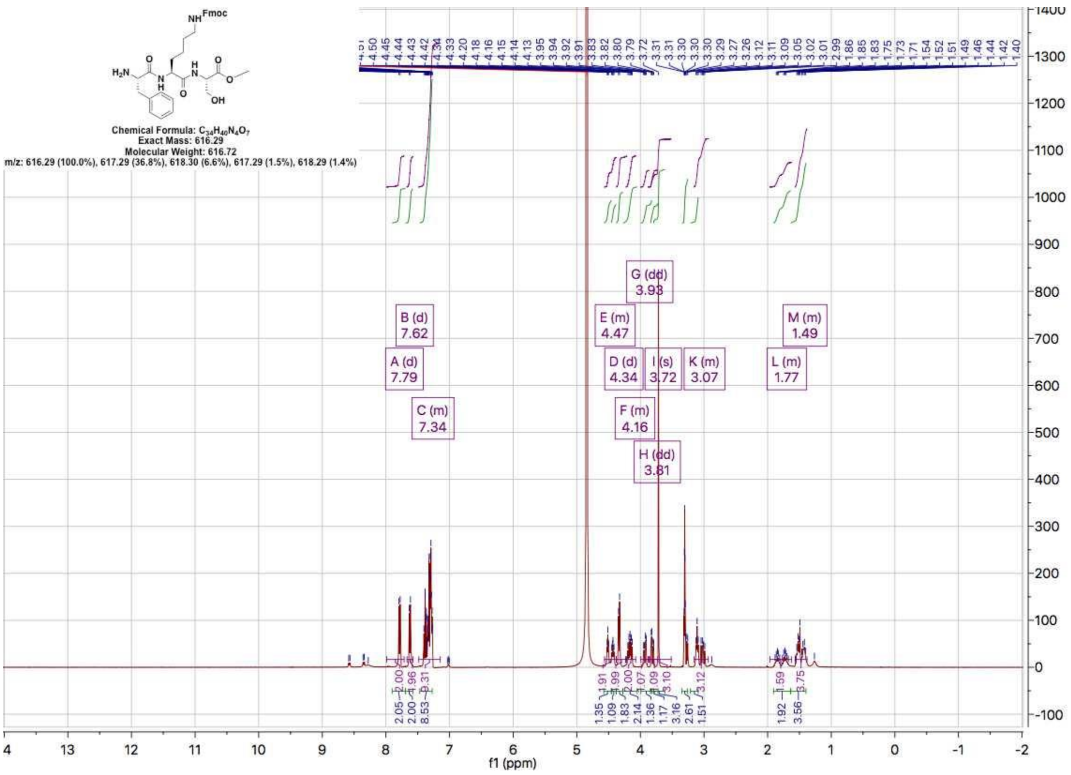

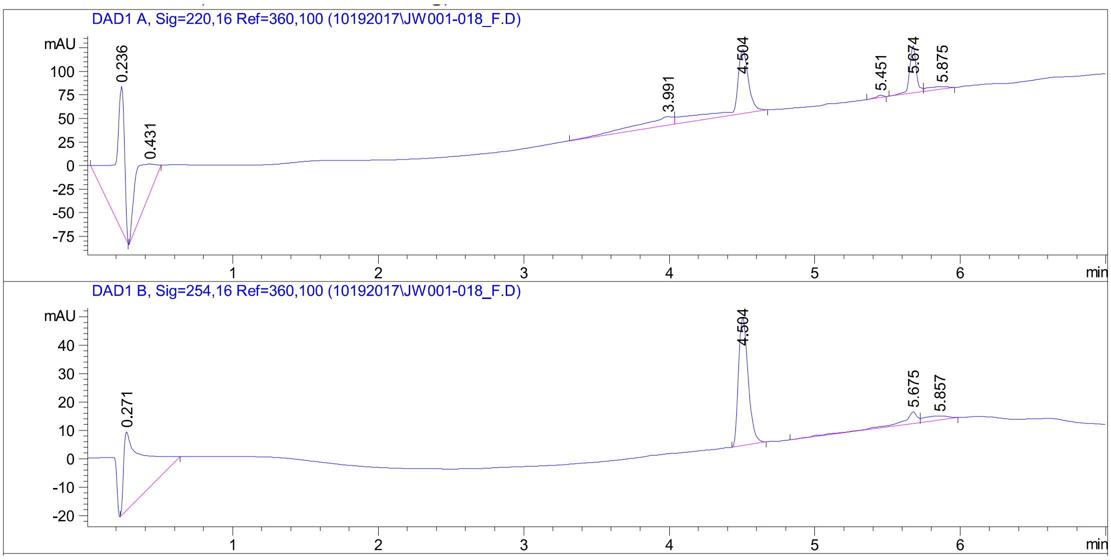

^{1}H NMR and LC-MS Spectra at 220 and 254 nm of Intermediate 3

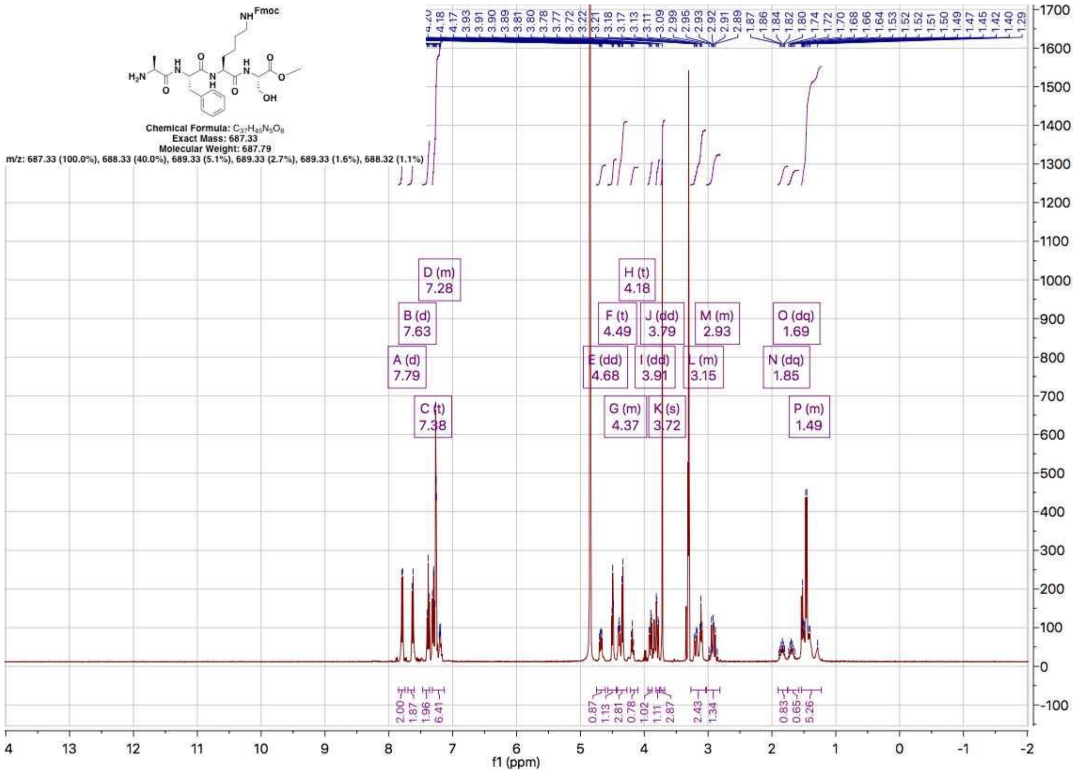

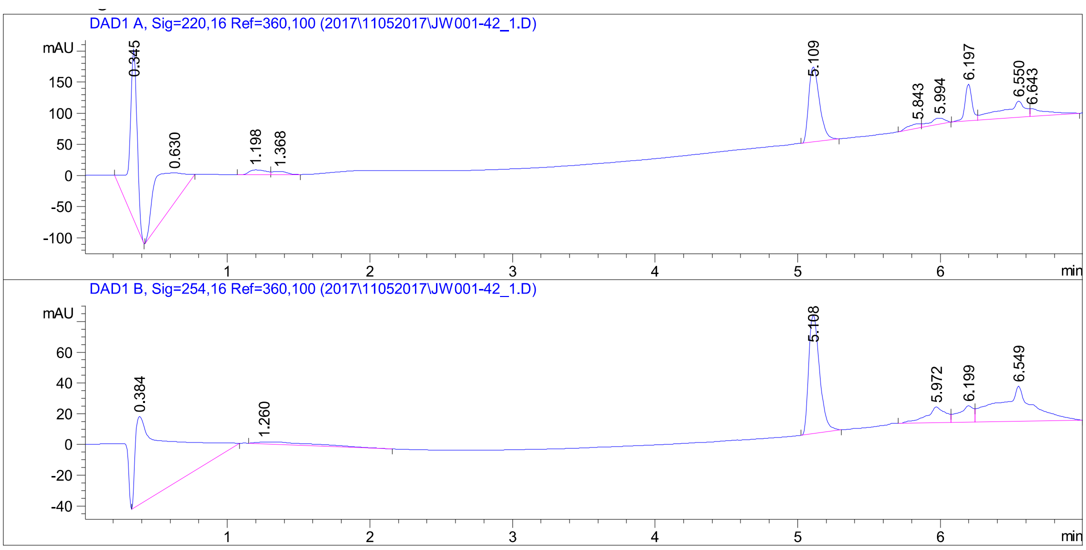

^{1}H NMR and LC-MS Spectra at 220 and 254 nm of Intermediate 4

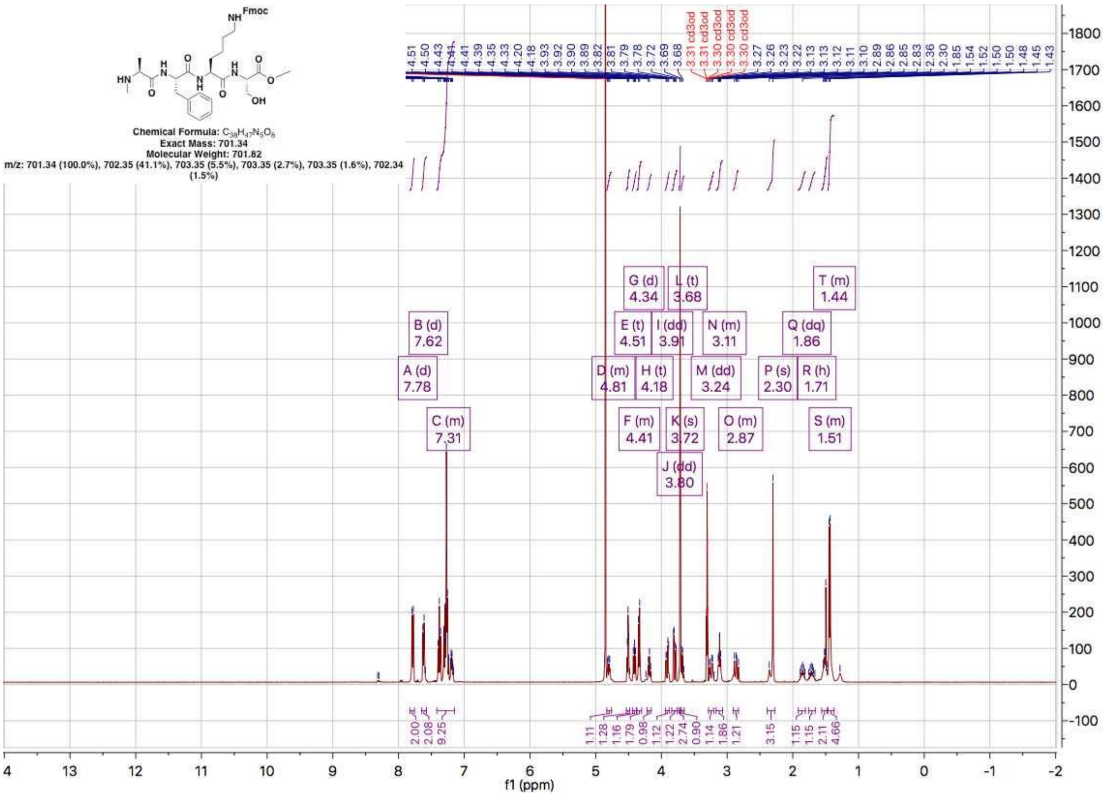

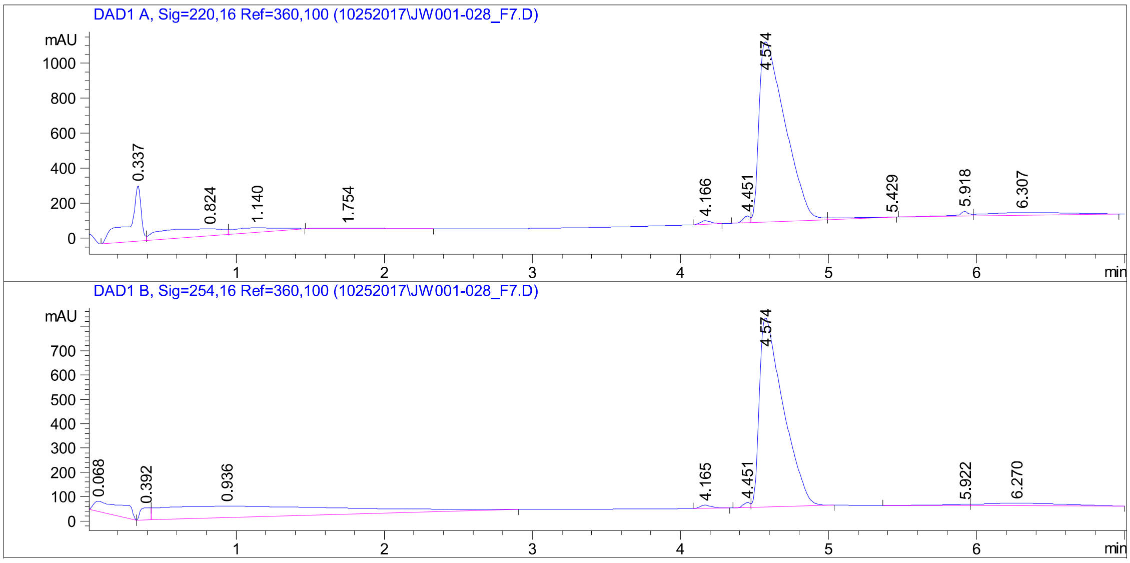

^{1}H NMR and LC-MS Spectra at 220 and 254 nm of intermediate 5

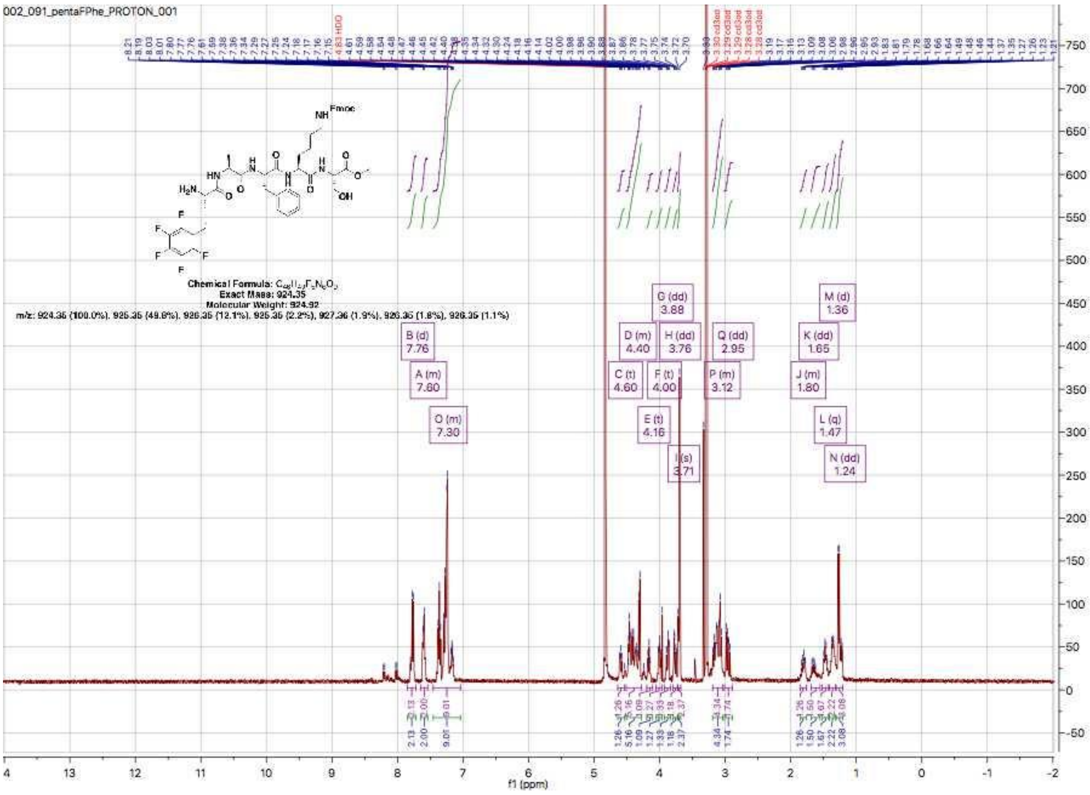

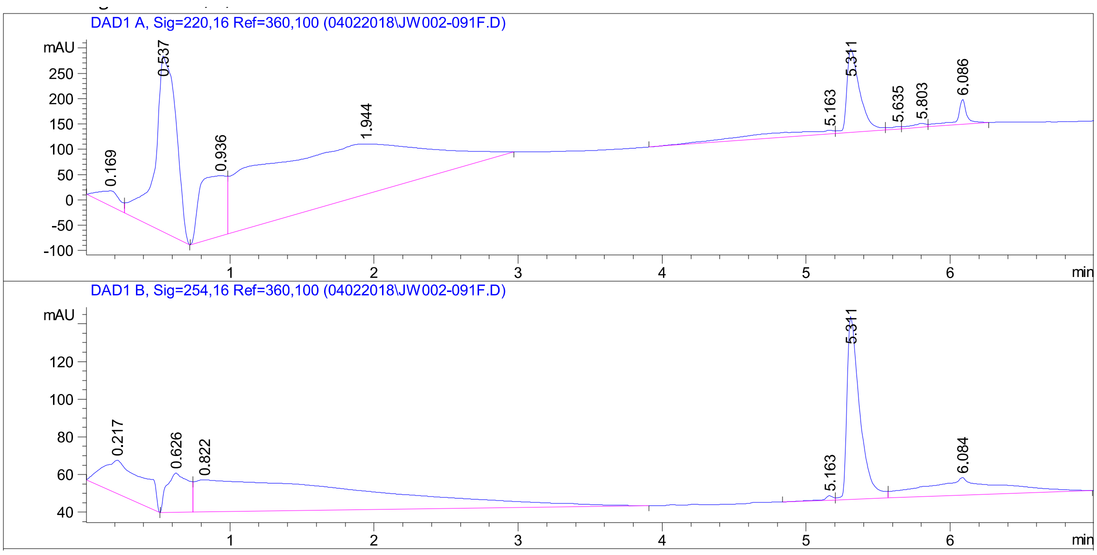

^{1}H NMR and LC-MS Spectra at 220 and 254 nm of intermediate 6

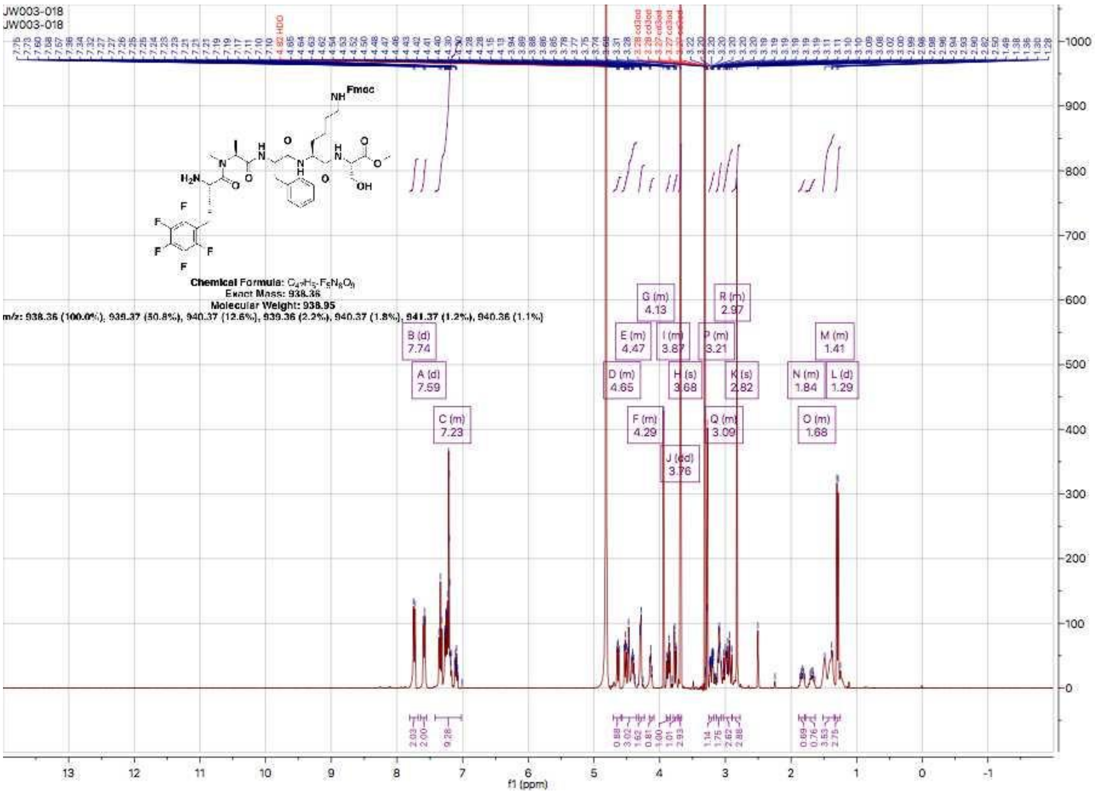

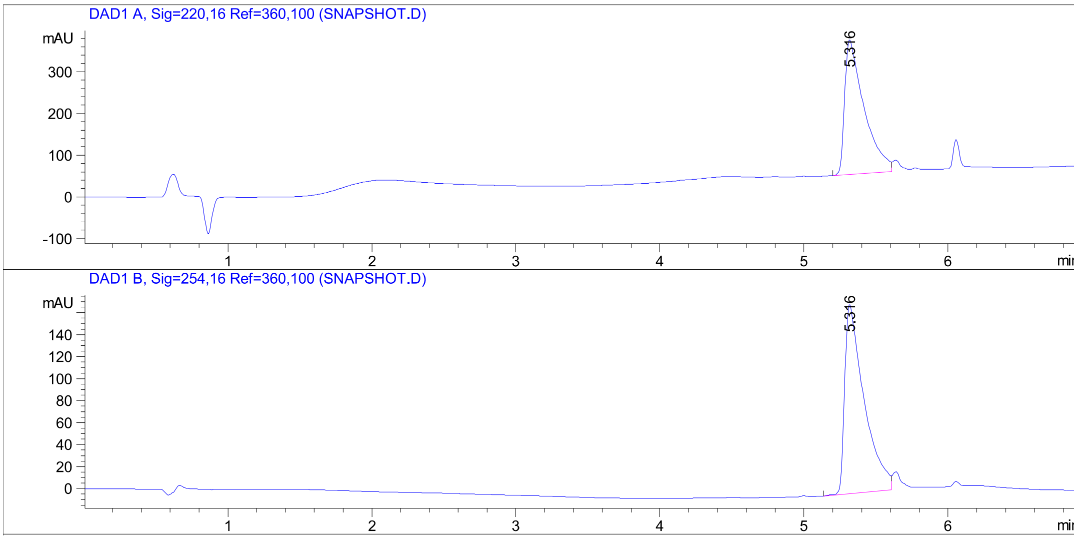

^{1}H NMR and LC-MS Spectra at 220, 254 nm and TIC of UNC6261

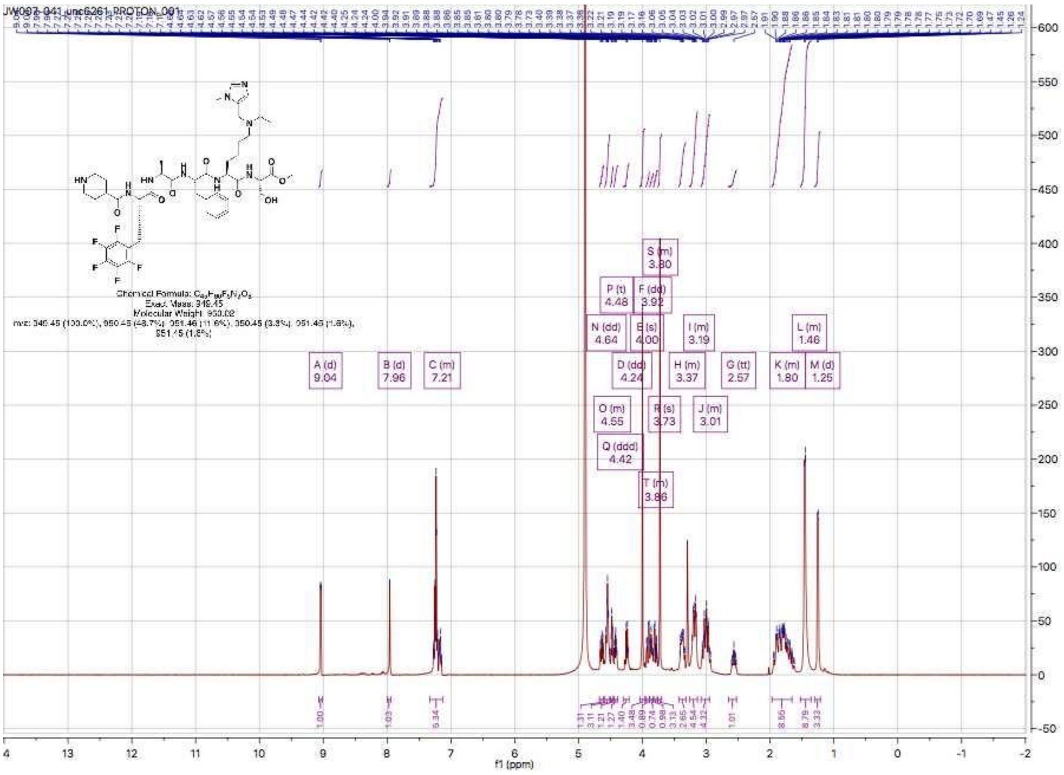

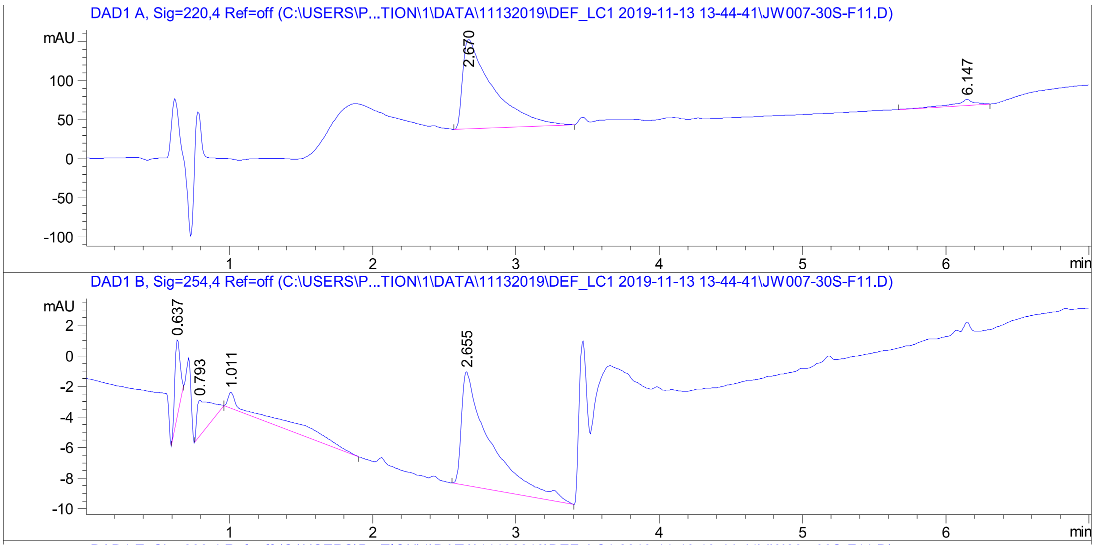

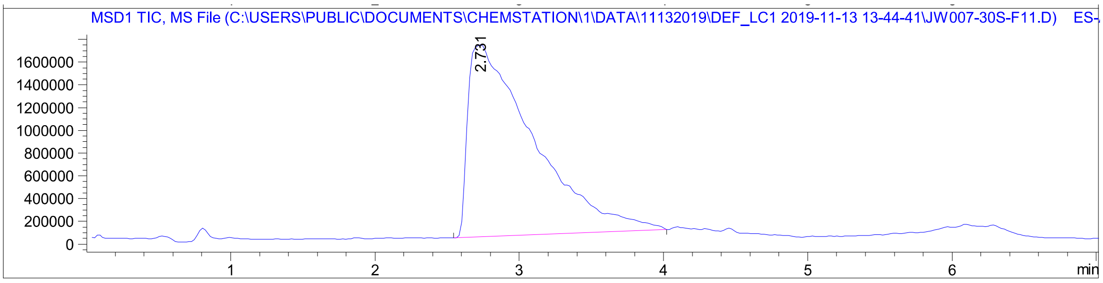

^{1}H NMR and LC-MS Spectra at 220 and 254 nm of UNC7394

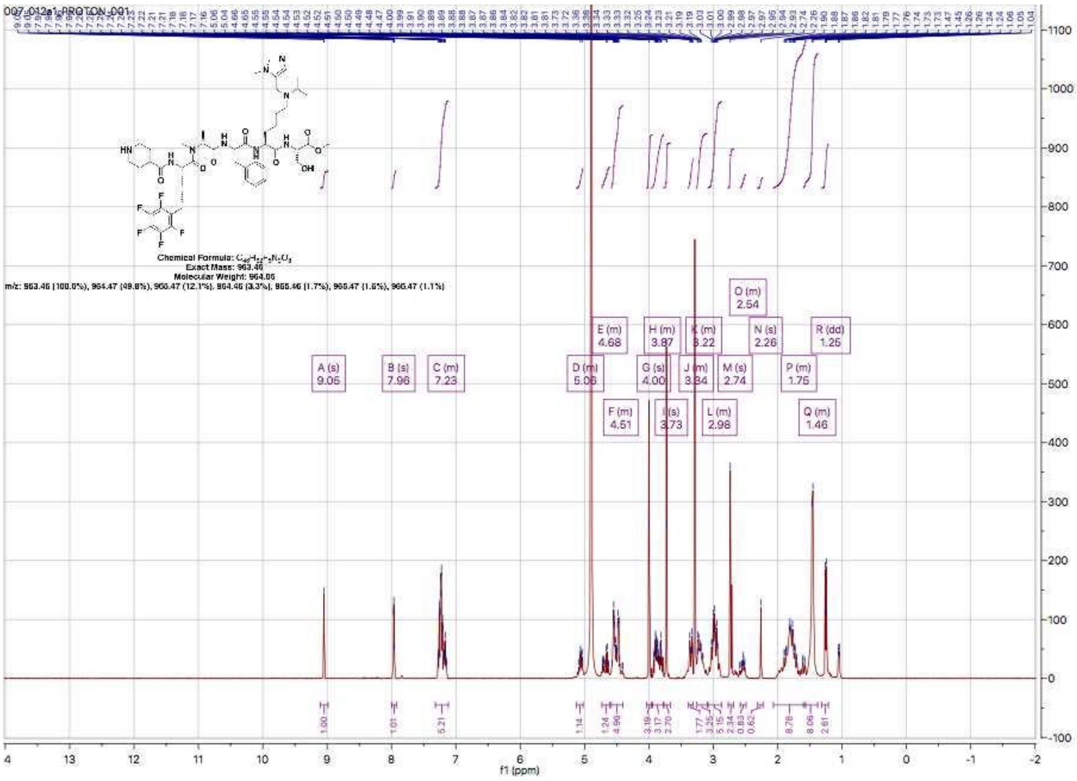

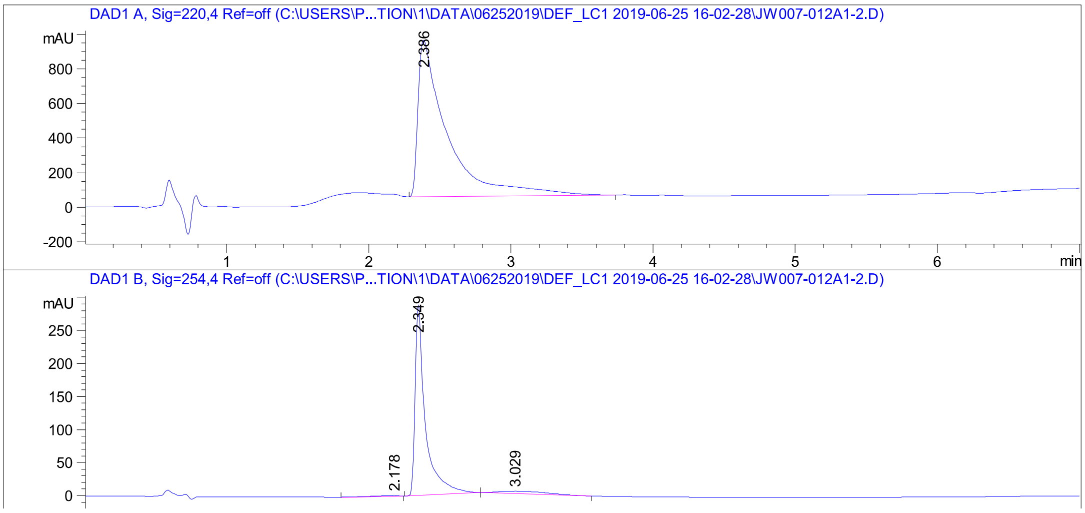

^{1}H NMR and LC-MS Spectra at 220 and 254 nm of UNC6261-Biotin

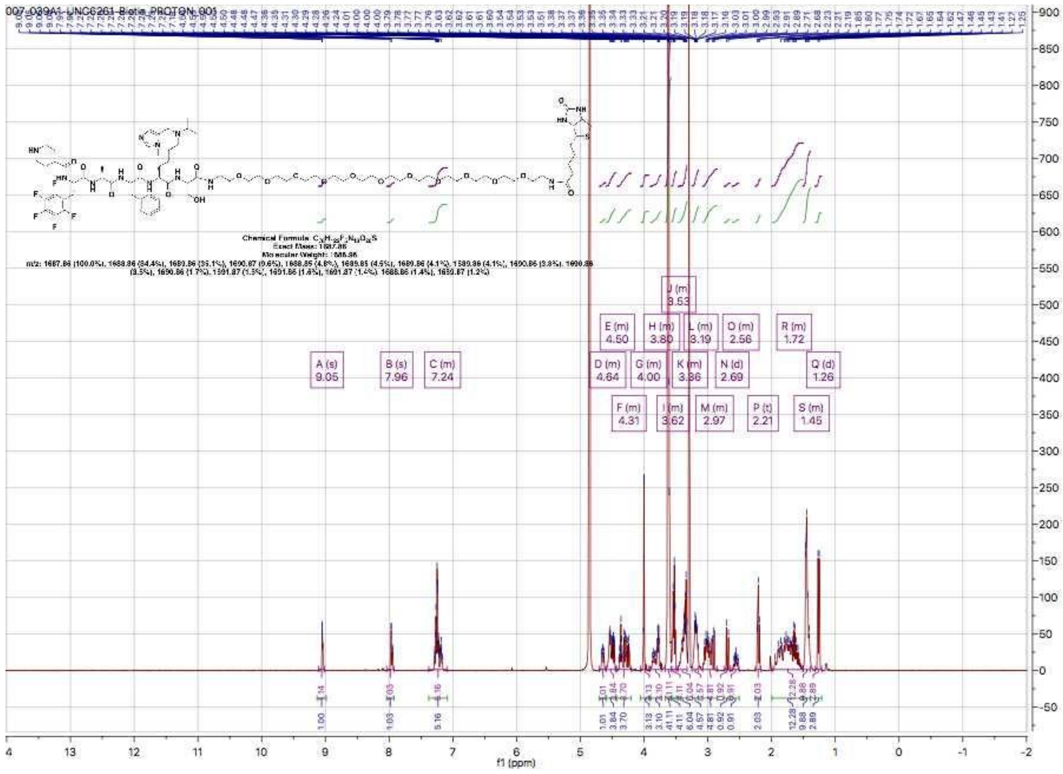

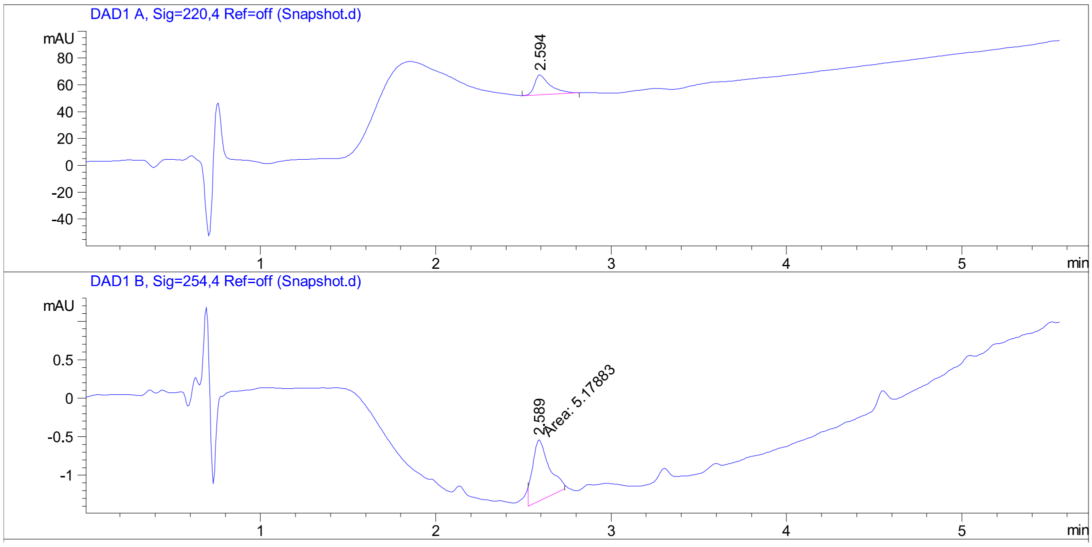

^{1}H NMR and LC-MS Spectra at 220 and 254 nm of UNC6261-CT

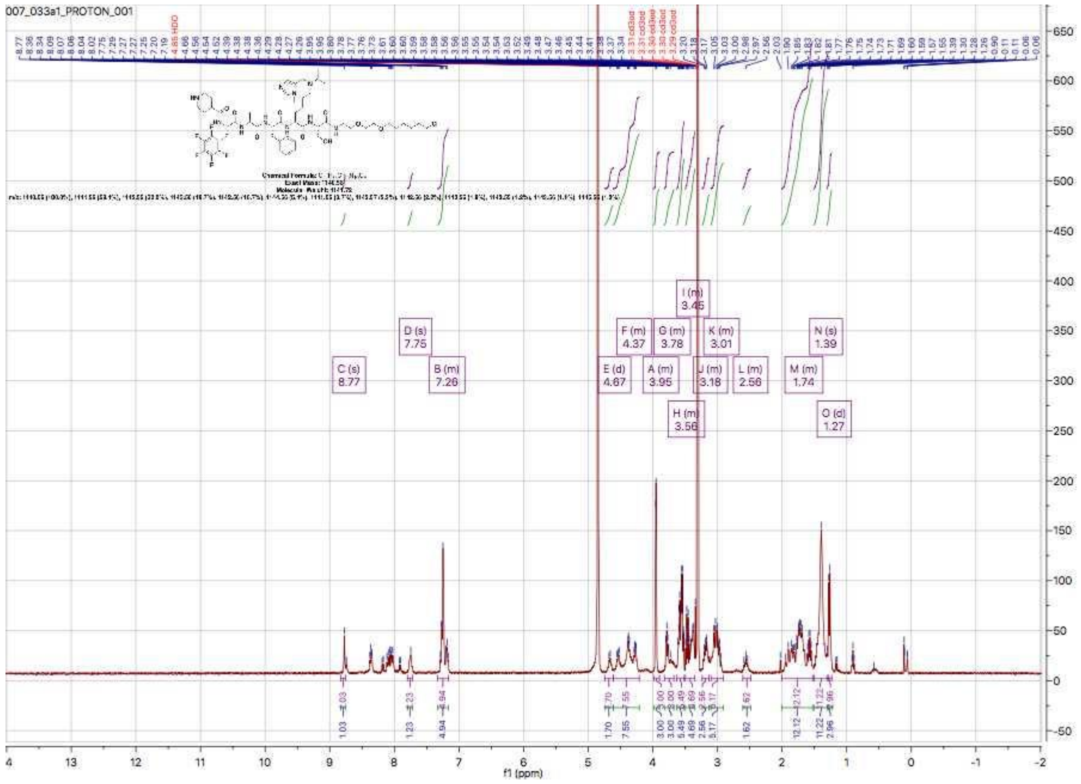

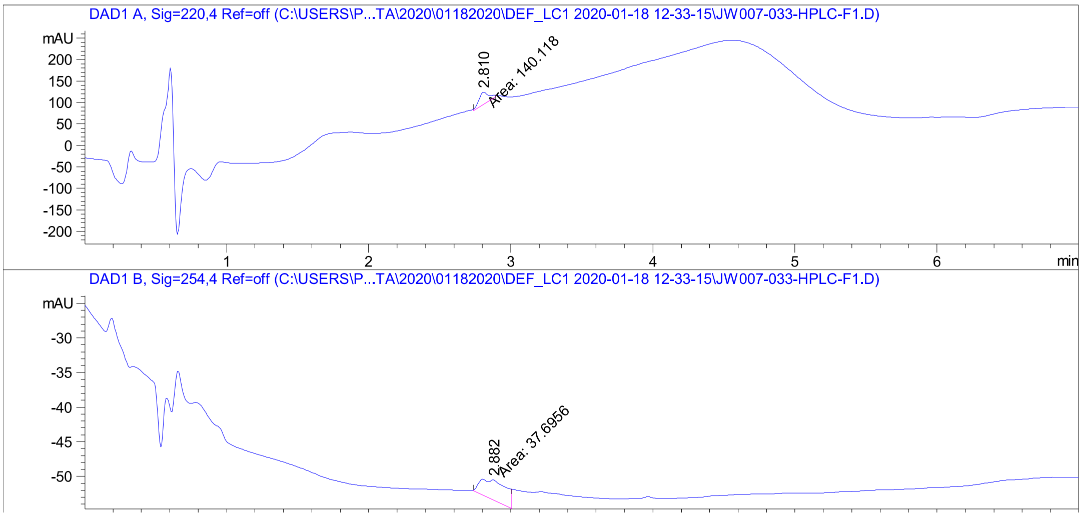

Uncropped blots

Uncropped blots related to Figure 1d.

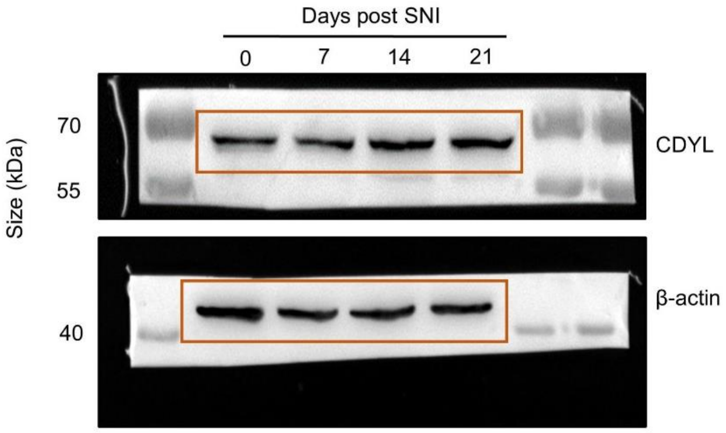

Uncropped blots related to Figure 1h.

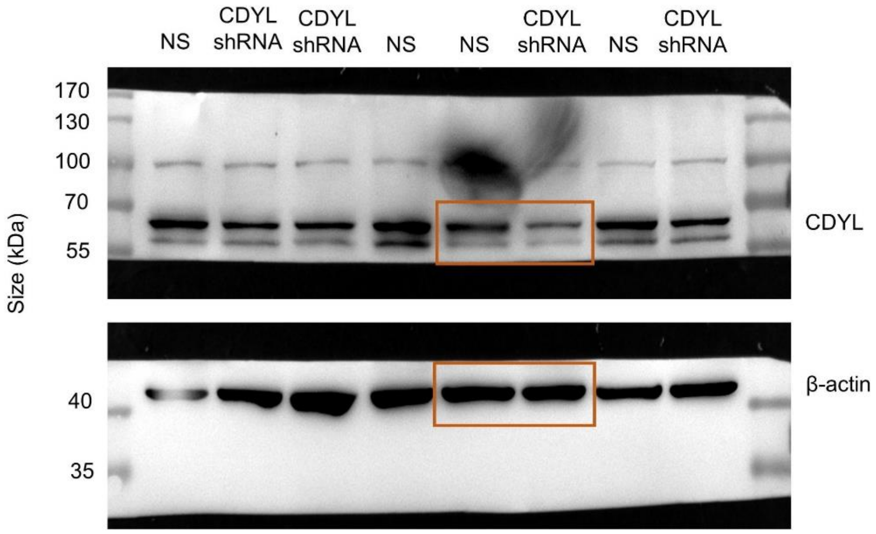

Uncropped blots related to Figure 2d.

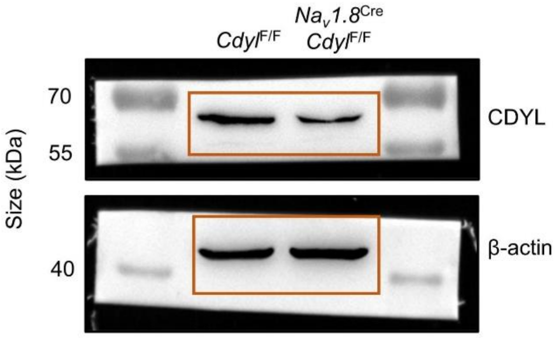

Uncropped blots related to Figure 2f.

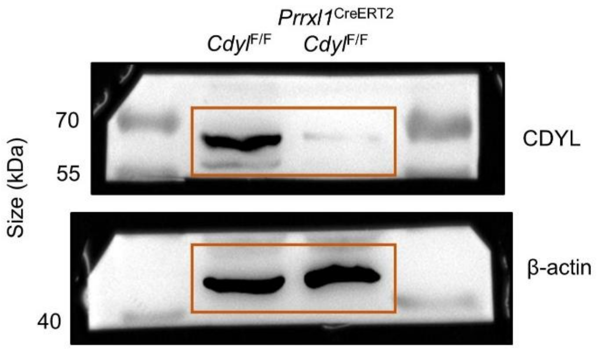

Uncropped blots related to Figure 4e.

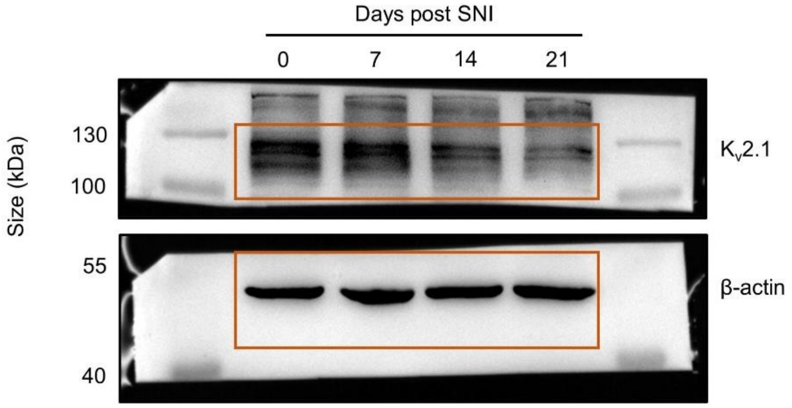

Uncropped blots related to Figure 4l.

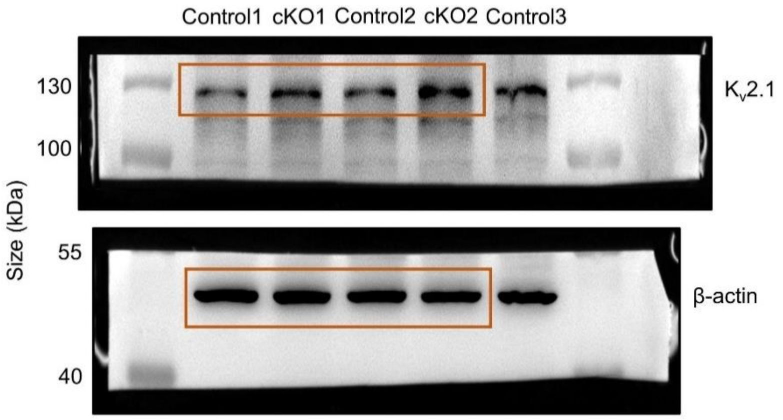

Uncropped blots related to Figure 6a.

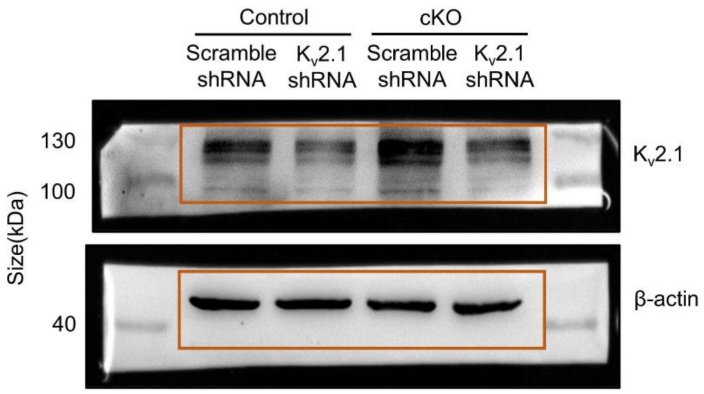

Uncropped blots related to Figure 7e.

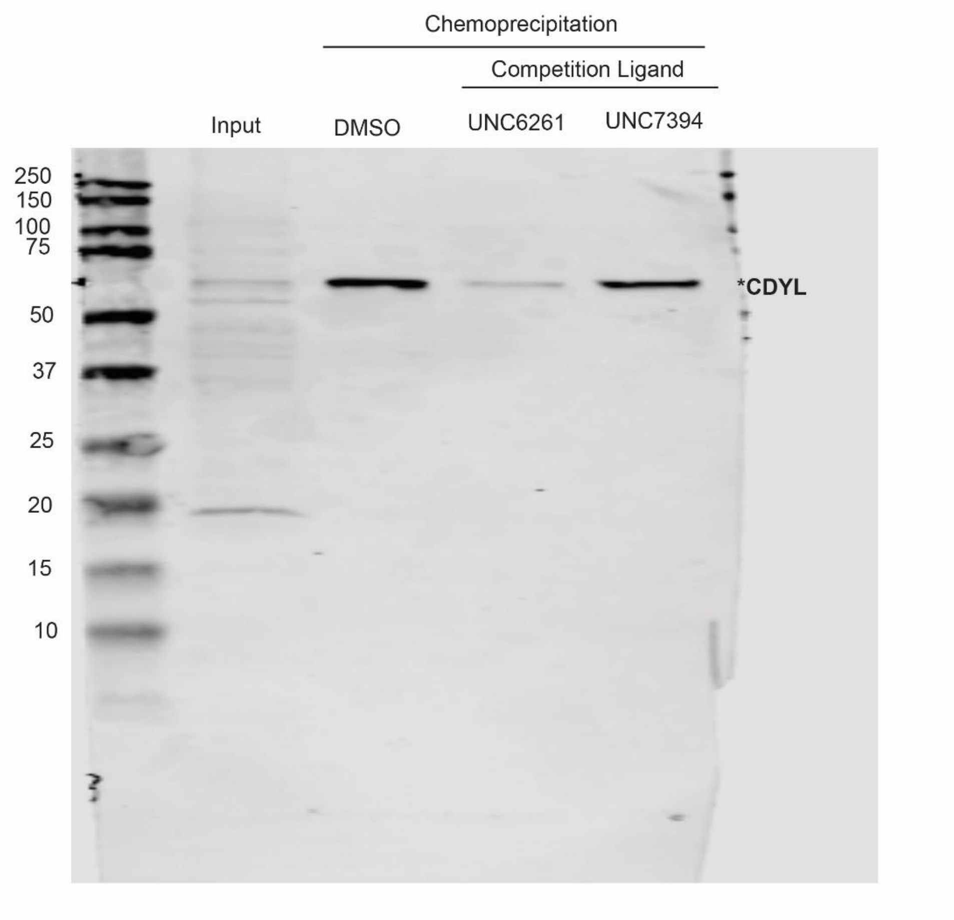

Uncropped blots related to Figure S1a.

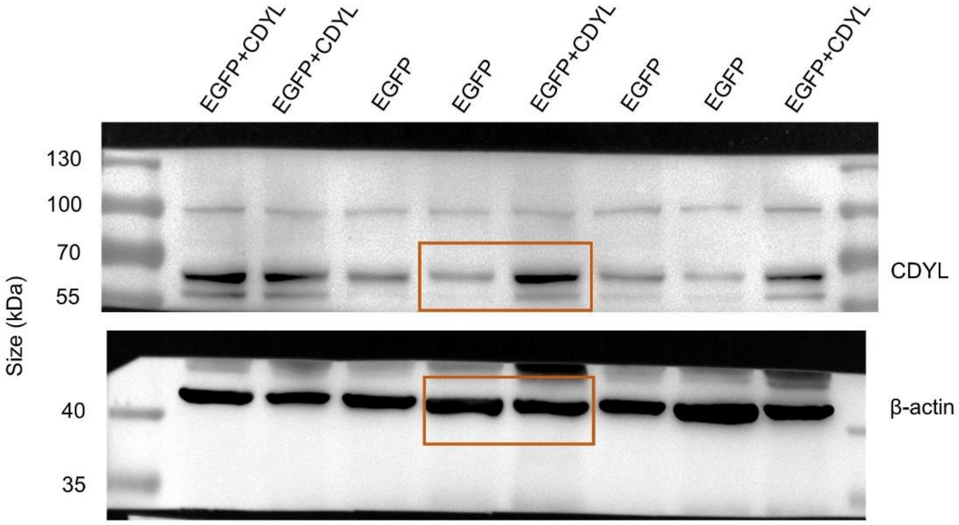

Supplement: Supplementary file 1 — Supporting Information [file ADVS-9-2104317-s001.pdf]
